# Macropolyhedral *syn*-B_18_H_22_, the “Forgotten” Isomer

**DOI:** 10.1021/jacs.3c05530

**Published:** 2023-08-02

**Authors:** Deepak Kumar Patel, B. S. Sooraj, Kaplan Kirakci, Jan Macháček, Monika Kučeráková, Jonathan Bould, Michal Dušek, Martha Frey, Christof Neumann, Sundargopal Ghosh, Andrey Turchanin, Thalappil Pradeep, Tomas Base

**Affiliations:** † DST Unit of Nanoscience (DST UNS) and Thematic Unit of Excellence (TUE), Department of Chemistry, Indian Institute of Technology, Madras, Chennai 600036, India; ‡ Institute of Inorganic Chemistry, The Czech Academy of Science, 25068 Rez, Czech Republic; § Institute of Physics, The Czech Academy of Science, 182 21 Prague 8, Czech Republic; ∥ Institute of Physical Chemistry Friedrich Schiller University Jena, 07743 Jena, Germany

## Abstract

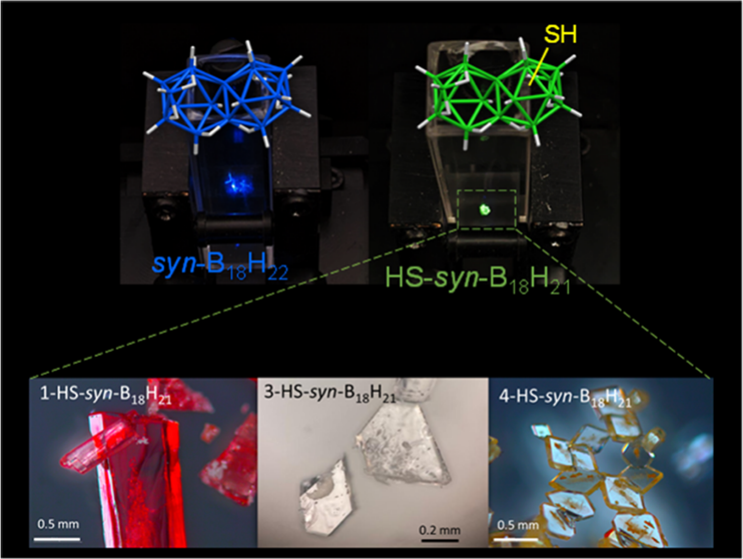

The chemistry and physics of macropolyhedral B_18_H_22_ clusters have attracted significant attention due to the interesting photophysical properties of *anti*-B_18_H_22_ (blue emission, laser properties) and related potential applications. We have focused our attention on the “forgotten” *syn*-B_18_H_22_ isomer, which has received very little attention since its discovery compared to its *anti*-B_18_H_22_ isomer, presumably because numerous studies have reported this isomer as nonluminescent. In our study, we show that in crystalline form, *syn*-B_18_H_22_ exhibits blue fluorescence and becomes phosphorescent when substituted at various positions on the cluster, associated with peculiar microstructural-dependent effects. This work is a combined theoretical and experimental investigation that includes the synthesis, separation, structural characterization, and first elucidation of the photophysical properties of three different monothiol-substituted cluster isomers, [1-HS-*syn*-B_18_H_21_] **1**, [3-HS-*syn*-B_18_H_21_] **3**, and [4-HS-*syn*-B_18_H_21_] **4**, of which isomers **1** and **4** have been proved to exist in two different polymorphic forms. All of these newly substituted macropolyhedral cluster derivatives (**1**, **3**, and **4**) have been fully characterized by NMR spectroscopy, mass spectrometry, single-crystal X-ray diffraction, IR spectroscopy, and luminescence spectroscopy. This study also presents the first report on the mechanochromic shift in the luminescence of a borane cluster and generally enriches the area of rather rare boron-based luminescent materials. In addition, we present the first results proving that they are useful constituents of carbon-free self-assembled monolayers.

## Introduction

**Figure 1:**
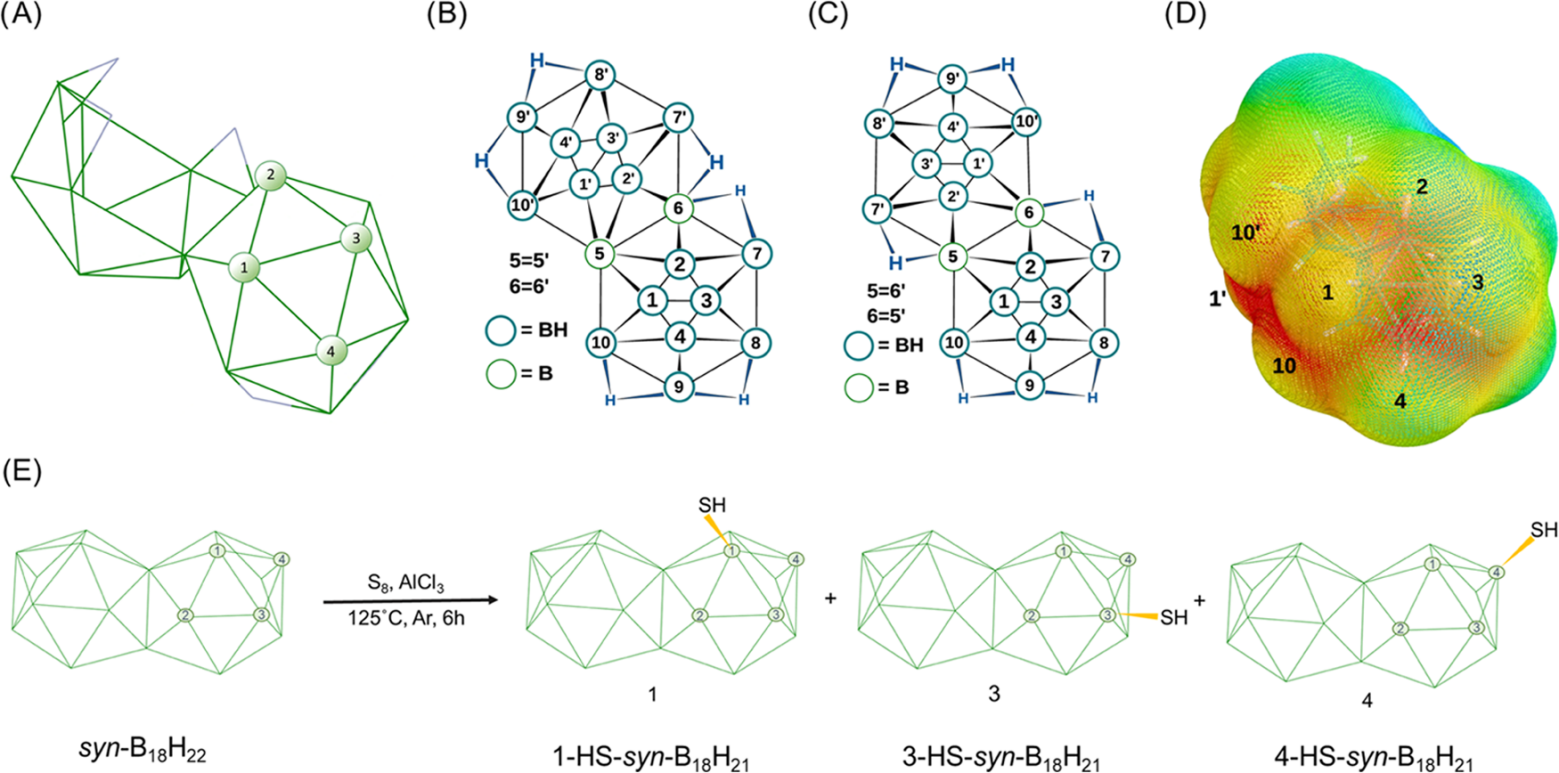
(A) Schematic of *syn*-B_18_H_22_ with partial numbering on one of the two subclusters. (B, C) Complete numbering systems of *syn*-B_18_H_22_ and *anti*-B_18_H_22_, respectively, in their net-like representation. (D) Electrostatic potential map of *syn*-B_18_H_22_ with selected BH vertices numbered. On the color scale, red shows areas with the highest negative potential, localized mainly in between vertices 1, 3, and 4. The red part in between the vertices 1′, 10, and 10′ represents the negative pole of the molecule. (E) Schematic of the synthesis of the three HS-*syn*-B_18_H_21_ isomers of *syn*-B_18_H_22_.

As the search for new and unusual molecules and materials in various fields intensifies, boron hydrides emerge as fascinating candidates with properties substantially different from those of organic molecules.^1−7^ Among the boron hydrides, the structural motif of the deltahedron is the most common. A prominent position belongs to the icosahedron due to the exceptional stability and geometry of the [B_12_H_12_]^2–^ dianion and its heteroatomic analogues, *e.g.*, the 12-vertex carbaborane [C_2_B_10_H_12_].^8,9^ Most boron hydride molecules with a number of boron atoms fewer than 12 take the form of a simple convex deltahedron or its open fragment, and they can thus be viewed as analogues of cyclic hydrocarbons.^10^ For boron hydrides with more than 12 skeletal atoms, the structures of the so-called macropolyhedral boranes are formed by the fusion of two or more polyhedra or polyhedral fragments, analogous to polycyclic hydrocarbons.^11^ Two of the largest known macropolyhedral boranes are the isomeric docosahydrooctadecaboranes B_18_H_22_.^12,13^ They exhibit a unique molecular structure with two open faces and six acidic bridging hydrogen atoms (μ*H*-BB).^14−16^ Their structural and chemical properties, together with their interaction with light, make B_18_H_22_ a promising candidate for a wide range of applications, from energy storage,^1,3^ semiconductor doping^4,17−20^ to nano- and optoelectronic devices.^21−23^ The molecular structure of B_18_H_22_ can be viewed as two decaborane molecules condensed together, with each subcluster sharing atoms B(5) and B(6) in the decaborane numbering system, in common (Figure 1B,C). The isomer *syn*-B_18_H_22_ on which this study focuses is a much less-studied (“forgotten”) system compared to its *anti*-B_18_H_22_ isomer, and it has a 2-fold symmetry axis due to the fusion of two {B10} units sharing the B(5)–B(6) edge so that B(5)≡B(5′) and B(6)≡B(6′) (Figure 1B); in the *anti*-B_18_H_22_ isomer, B(5)≡B(6′) and B(6)≡B(5′), which results in the inversion symmetry (Figure 1C).^15,16,24^ What has stimulated most of the recent interest in *anti*-B_18_H_22_ and its substituted derivatives has mainly been their luminescence properties.^25−38^ Our interest in the “forgotten”, nonluminescent isomer, *syn*-B_18_H_22_, has been stimulated mainly by its unique geometry and size with respect to its use as constituents of purely borane, carbon-free self-assembled monolayers and its further use toward 2-dimensional membranes^39^ with thickness below 1 nm and with a 3D-aromatic character, as well as capping ligands of atomically precise metal nanoclusters, a newly emerging class of materials with adjustable geometry, size, and properties.^40,41^

Previously, we reported the thiol derivatives of decaborane (*nido*-B_10_H_14_), specifically [1-HS-*nido*-B_10_H_13_], [2-HS-*nido*-B_10_H_13_], and [1,2-(HS)_2_-*nido*-B_10_H_12_] as reactive building blocks for self-assembled monolayers (SAMs).^42,43^ Although the macropolyhedral cluster *anti*-B_18_H_22_ has been extensively explored over the last decade, for various applications including lasers^7^ and semiconductor dopants,^4,18,19^ the *syn*-B_18_H_22_ isomer has remained relatively unexplored, perhaps due to its supposed lack of luminescence. For numerous reasons mentioned foregoing, we have synthesized three new monothiol-substituted derivatives: [1-HS-*syn*-B_18_H_21_] **1**, [3-HS-*syn*-B_18_H_21_] **3**, and [4-HS-*syn*-B_18_H_21_] **4**. We have found interesting interactions in the supramolecular structures, observed the changes in the molecular structure caused by substitution at different vertices, and, surprisingly and interestingly, observed luminescence not only in the novel thiol derivatives but also in the parent *syn*-B_18_H_22_ molecule, which, until now, has been presented in various reports^7,26^ as the nonluminescent isomer of *anti*-B_18_H_22_. Also, we have observed that substitution affects its photophysical properties extensively. The s*yn*-B_18_H_22_ cluster functionalization thus promises new directions for novel materials with a range of properties and uses.

Boron hydrides as functional molecules, going beyond alkanethiols in their structural diversity for self-assembled monolayers (SAMs), are potentially very interesting and stable 2D materials for filtration technology due to their symmetry, limited conformational flexibility, well-defined, and different to-carbon-chemistries and other unique aspects such as thickness, and porosity adjusted in the subnanometer range or extraordinary thermal and chemical stability.^42−48^ These engineered cage molecules may interact in different ways to provide control over surface interactions.^44,49^ The advantage of boron hydrides as building blocks of SAMs over many linear chain systems is that simple cage molecules do not exhibit domain boundaries caused by the orientation of molecular tilt, with respect to the surface normal.^42,45,47^ The first boron cage, Cs_2_[B_12_H_11_SH], was employed to study SAMs on gold surfaces in 1998.^47^ Mercapto-functionalized cage molecules provided great control over surface responses, a higher order of supramolecular assembly, and eventually a precise three-dimensional assembly over a surface.^42,45,47^ However, the SAMs of boron hydrides still remain much less explored than those of alkanethiol derivatives, and macropolyhedral borane derivatives assembled on metal or other substrate surfaces have not been investigated yet. Keeping all of this in mind, we delved into the world of macropolyhedral boron hydrides, explored the HS functionalization of *syn*-octadecaborane, and investigated the impact of these modifications on its physical and chemical properties, together with the first preliminary view of these molecules as building blocks of carbon-free SAMs on a flat metal surface.

## Results and Discussion

**Figure 2:**
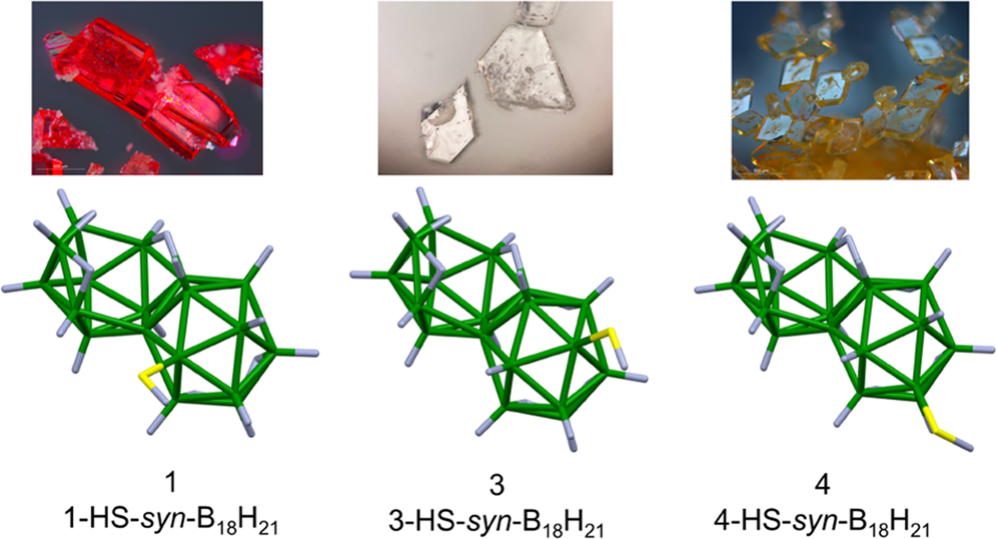
X-ray determined structures of three synthesized thiol isomers of *syn*-B_18_H_22_.

Three thiol isomers of the octadecaborane cluster [1-HS-*syn*-B_18_H_21_] **1** (two polymorphic structures labeled as PM1a and PM1b), [3-HS-*syn*-B_18_H_21_] **3**, and [4-HS-*syn*-B_18_H_21_] **4** (two polymorphs labeled PM4a and PM4b) were synthesized directly from *syn*-B_18_H_22_ by heating it with sulfur in the presence of anhydrous aluminum trichloride for 6 h at 125 °C under an Ar atmosphere. The crude product contained a mixture of monothiolated isomers and unreacted *syn*-B_18_H_22_, and these were all separated using standard chromatography on a silica gel column with diethyl ether as the eluent. The separated products were crystalline, with many single crystals suitable for X-ray diffraction. Three isomers were structurally resolved by X-ray diffraction (Figure 2), with two of the isomers found to be in two polymorphic structures. All of these results gave us a very good possibility to look at the effect of substitution on the parent *syn*-B_18_H_22_ molecular structure as well as to investigate the molecular packing of these isomers in order to set a basis for further investigation toward the origin of their luminescent properties.

### Molecular Structure, the Effect of a Substituent

**Figure 3:**
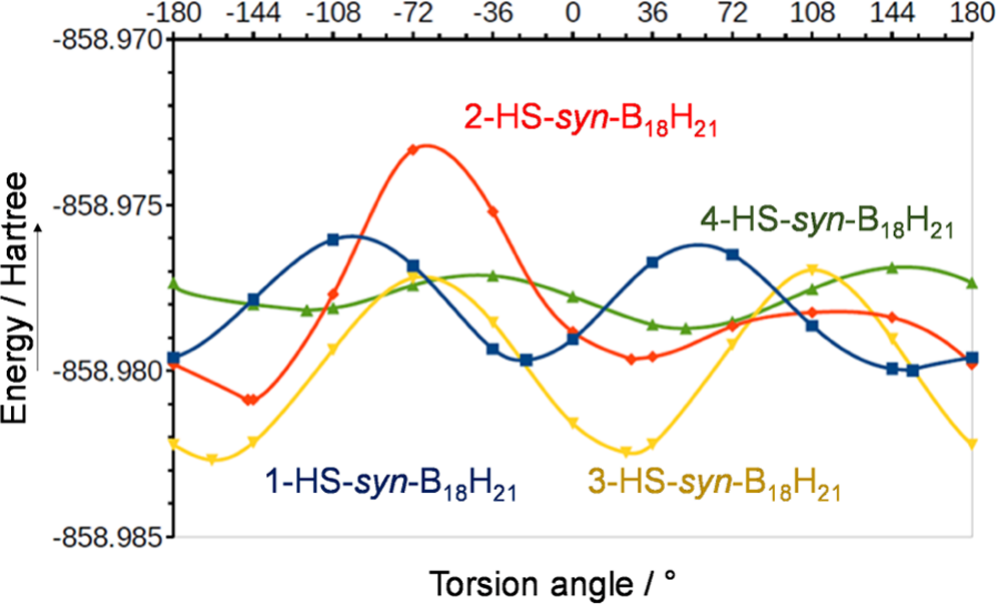
Plot of energies of various HS-rotamers for all four computationally analyzed HS-*syn*-B_18_H_21_ isomers. Torsion angles were defined as positive in the B(1)-B(2)-B(3)-B(4) direction, between atoms B(2)-B(1)-S(1)-H(S) for isomer **1**, B(6)-B(2)-S(2)-H(S) for the experimentally unobtained isomer **2**, B(2)-B(3)-S(3)-H(S) for **3**, and B(1)-B(4)-S(4)-H(S) for **4**.

All of the three isomers **1, 3**, and **4** were prepared in quantities ranging from a few tens to hundreds of milligrams. Isomer **4** was dominating the crude product, but chromatography and crystallization from diethyl ether yielded many well-developed single crystals of all three and two of them in two polymorphic forms. Crystallization experiments from diethyl ether yielded all three isomers pure, and they were further characterized using ^1^H and ^11^B NMR, MS, IR, and X-ray studies. Computational analysis of the electrostatic potential map suggests that the thiol (HS-) group might substitute positions B1, B2, B3, and B4 of the *syn*-B_18_H_22_ cluster due to the relatively high negative charge localization in that part of the molecule (Figure 1D). Conformational analysis and mutual comparison of the respective energies of all four isomers and their most stable conformers also do not show significant differences, and isomer 3-HS-*syn*-B_18_H_21_ seems to be even more stable than isomer 4-HS-*syn*-B_18_H_21_ (Figure 3). Experimentally, we have found that thiol substitution follows the order B4 > B1 ≅ B3, and, interestingly, we have not observed the 2-HS-*syn*-B_18_H_21_ isomer.

**Figure 4:**
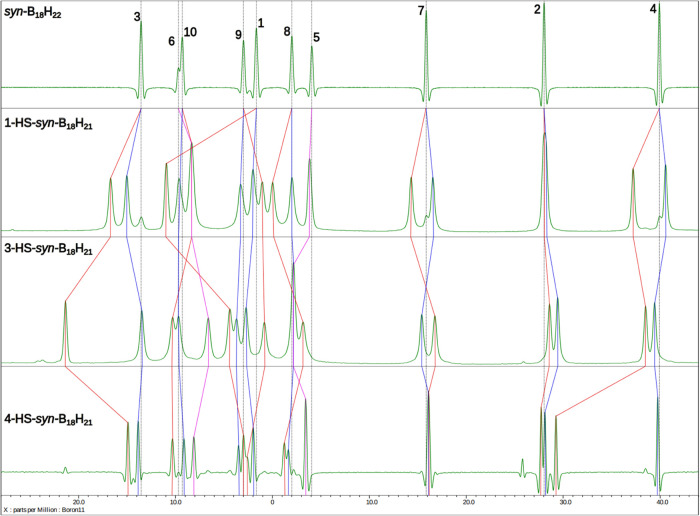
Experimentally decoupled ^11^B{^1^H} NMR spectra of *syn*-B_18_H_22_ and HS-*syn*-B_18_H_21_ isomers.

The molecular composition of the thiol derivatives was confirmed by the positive ion mode ESI-MS analysis. The monocationic species with the mass value centered at *m*/*z* 248.3152 was identified as HS-B_18_H_21_, while the *m*/*z* value at 216.3422 corresponds to B_18_H_21_, which can be attributed to the loss of an HS fragment (Figure S28). The introduction of a thiol (HS-) group to B_18_H_22_ breaks the twofold symmetry as manifested in its ^11^B{^1^H} NMR spectra by the splitting of the peaks that had intensity 2 in the parent compound. The resonance of the substituted boron atom is easily identifiable in comparison to the decoupled ^11^B{^1^H} spectrum^50^ with the simple coupled ^11^B one as a third singlet in addition to the peaks of boron vertices 5 and 6. The thiol group causes a deshielding of the substituted atom and moves its peak around 10 ppm downfield; the effect of the substitution on other boron atoms is weaker and more complex (Figure 4). Unsurprisingly, the NMR signals of the atoms of the unsubstituted subunit (numbers with prime) are, in general, less affected than those of the substituted subunit (labeled by simple numbers), often observed at positions almost unchanged from the parent borane. One remarkable exception is position 2, which is very little affected by the substitution in all our compounds, where in **3** the peak of 2′ is shifted upfield more than that of position 2. The calculation of the NMR spectra at the DFT level (Table S2) reproduces well only the most prominent features of the spectra, often failing to capture the more sophisticated ones, and several times even the relative positions of the peak of an atom of the substituted subunit (n) with respect to its symmetry counterpart from the unsubstituted subunit (n′) differ between the calculation and the experiment (Figures S11, S17, and S24). Additionally, FT-IR spectroscopy corroborates the structure of isomers **1**, **3**, and **4** (Figure S29). A strong band at ∼2560 cm^–1^ can be attributed to the terminal B–H and SH stretching vibrations. Another strong band at about 1490 cm^–1^ is characteristic of the bridging μ-B*H*B vibrations.

**Figure 5:**
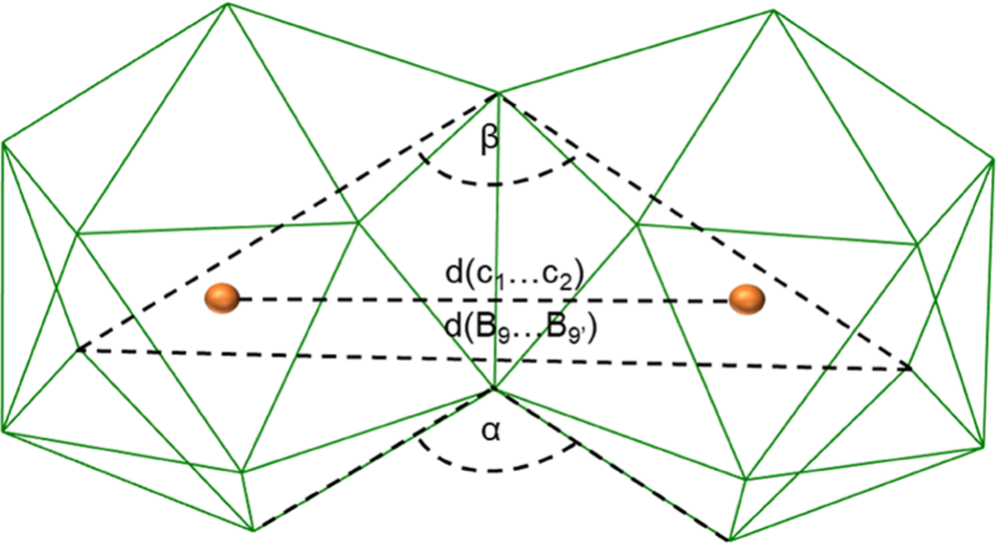
Schematic illustration of selected distances and bond angles of *syn*-B_18_H_22_ and its thiol derivatives.

Single-crystal X-ray diffraction (SC-XRD) investigation of all five thiol isomers/polymorphs of HS-*syn*-B_18_H_21_, structures **1** (PM1a and PM1b), **3**, and **4** (PM4a and PM4b), reveal the positions of all of the heavier elements (B and S) in the compound. After refining the heavier atoms anisotropically, the remaining electron-density map reveals all of the hydrogen atoms in the cluster. After isotropic hydrogen atom refinement, the maximum electron-density peak for the thiol hydrogen atoms was near the sulfur atom; however, the final refinement was done with a riding model. Both the [1-HS-*syn*-B_18_H_21_] **1** and [4-HS-*syn*-B_18_H_21_] **4** isomers were found to exist in two polymorphic structures PM1a, PM1b and PM4a, PM4b, respectively. Although the 18-vertex cage structure of the parent *syn*-B_18_H_22_ remains intact after thiolation, a more detailed comparative analysis of the interatomic distances and angles of all six single-crystal structures, **1** (PM1a, PM1b)**, 3**, and **4** (PM4a, PM4b), and the parent *syn*-B_18_H_22_ demonstrates that the presence of the thiol substituent produces an obvious effect on the clusters′ geometry (see Table 1). This effect is difficult to spot if we look at a local vertex to adjacent vertex distances in close proximity to the substitution. However, as shown in Figure 5, we here selected geometrical parameters that cover the accumulation of small changes across the whole molecules, such as the *d*(B_9_-B_9′_) distance, or related angles that reflect the changes in mutual orientation of the two 10-vertex subclusters, and we can thus demonstrate and evaluate the substituents′ effect on the molecular structure. The substitution at position 3 of *syn*-B_18_H_22_ has a significant effect on isomer 3-HS-*syn*-B_18_H_21_**3.** The distances between the respective centroids, c_1_–c_2_, as well as between the vertices from opposite ends of the molecule, B(9)–B(9′), increased compared to the parent *syn*-B_18_H_22_. Intramolecular angles α and β, specified in Figure 5, also showed expansion as the direct consequence of the substitution (for detail, see Figure S35), causing a change in the cage geometry, which may be disadvantageous for the stability of the cluster and responsible for the lower yields of isomers **1** and **3** compared to that of isomer **4** (Table 1). All of the above changes in distance between centroid c_1_–c_2_, intracage B(9)–B(9′), and intracluster cage angle (α and β) are due to the accumulation of small changes in the B–B bond length of the cage (for more detail, see Table S5).

**Table 1:** Selected Centroid Distance [Å] (c_1_: Centroid of 10 B Atoms Representing One Subcluster of the *syn*-B_18_H_22_ Cage, c_2_: Centroid of 10 B Atoms of Another Fused Borane Cage), Intracage d(B_9_···B_9′_) Bond Distance [Å], and Bond Angle for [1-HS-*syn*-B_18_H_21_] 1 (PM1a and PM1b), [3-HS-*syn*-B_18_H_21_] 3, and [4-HS-*syn*-B_18_H_21_] 4 (PM4a and PM4b) with Comparison Data of *syn*-B_18_H_22_

	d(c_1_···c_2_)		d(B_9_···B_9′_)		α(B_10_B_5_B_10′_)		β(B_9_B_6_B_9′_)	
isomers/PMs	expt.	cal.	expt.	cal.	expt.	cal.	expt.	cal.
*syn*-B_18_H_22_	3.332	3.312	6.343	6.298	126.02	127.50	125.97	127.07
1-HS-*syn*-B_18_H_21_ (PM1a)	3.342	3.318	6.370	6.299	129.53	128.50	128.52	127.64
1-HS-*syn*-B_18_H_21_ (PM1b)	3.334	3.318	6.348	6.299	127.85	128.50	126.78	127.64
3-HS-*syn*-B_18_H_21_	3.452	3.314	6.383	6.301	130.18	127.73	128.96	127.18
4-HS-*syn*-B_18_H_21_ (PM4a)	3.330	3.314	6.345	6.300	128.44	127.40	128.20	127.14
4-HS-s*yn*-B_18_H_21_ (PM4b)	3.330	3.314	6.356	6.300	127.94	127.40	127.57	127.14

### Supramolecular Structure

**Figure 6:**
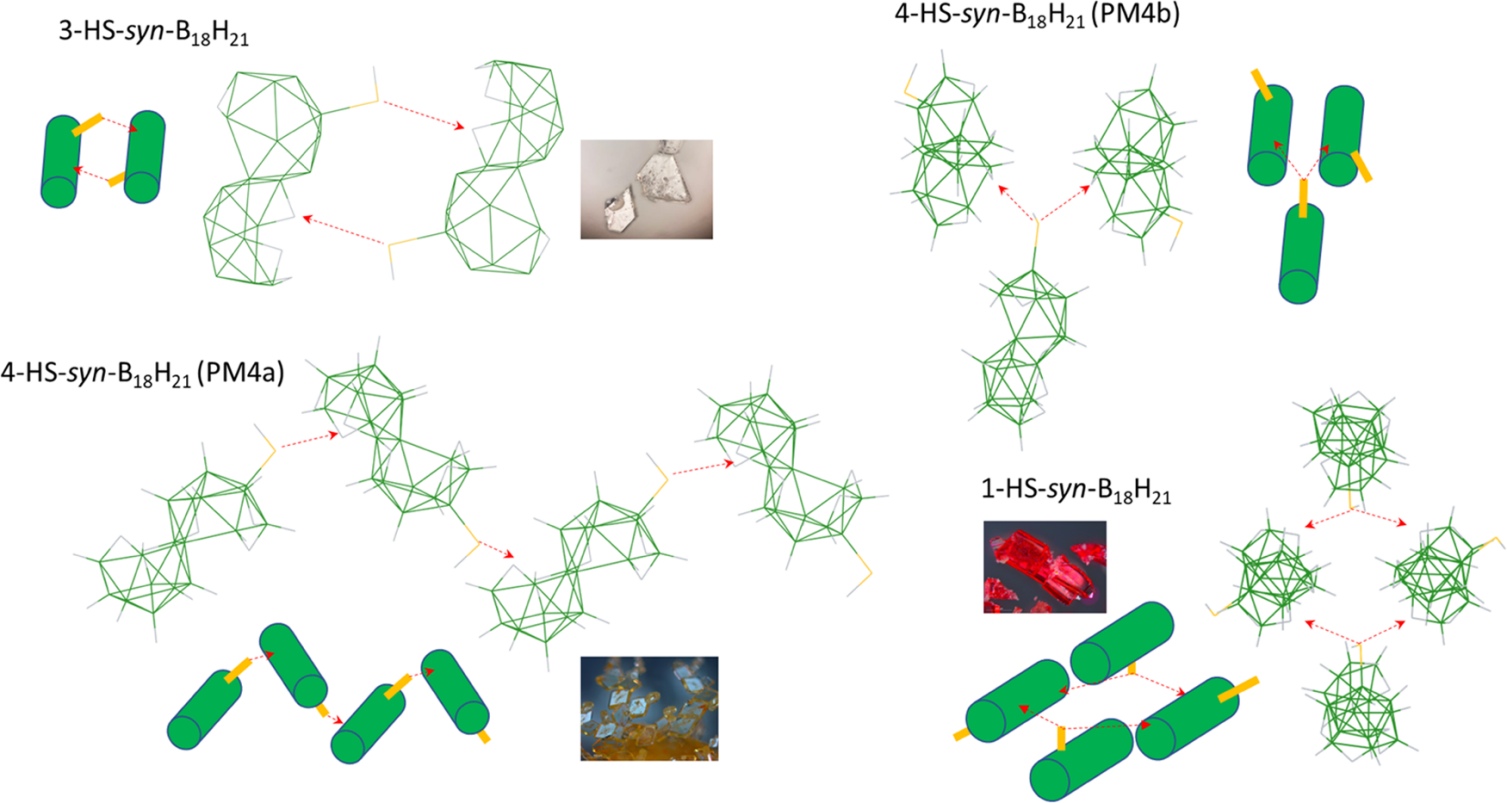
–*S*(H)···μ*H*-BHB interaction observed in the supramolecular structures of HS-*syn*-B_18_H_21_ isomers/polymorphs.

The successful X-ray diffraction analysis of five different single crystals provided sufficient data for the investigation of intermolecular interactions. In all of the synthesized thiol isomers, the respective sulfur atoms bear high electron densities and show interactions with the acidic bridging hydrogen atoms, μ-B*H*B, which manifest their presence by strong bands at about 1490 cm^–1^ in the IR spectra. In addition to their acidic nature, *i.e.*, the bridging hydrogen atom bearing a relatively high positive charge, it was interesting to see the supramolecular structures and how this interaction influences the orientation of the clusters. Figure 6 shows four out of five of the analyzed structures, and in all of them, the -*S*(H)···μ-B*H*B- interaction dominates the packing forces. In the single-crystal supramolecular structure of isomer 3-HS-*syn*-B_18_H_21_**3**, this interaction leads to a pair of molecules directly connected by two such interactions. The sulfur atom of the substituted subcluster shows an interaction with the bridging hydrogen atoms of the unsubstituted subcluster of another molecule. This isomer was found only in one polymorphic form. In comparison, the most preferred isomer 4-HS-*syn*-B_18_H_21_**4** exhibits a similar interaction in which the sulfur atom of one molecule interacts with the bridging hydrogen atoms of another molecule to form a zigzag chain or, in a second polymorph of this isomer, the sulfur atom shows an interaction with the bridging hydrogen atoms of two other molecules, leading to a fork-like arrangement. Both supramolecular structures are easy to distinguish. The last isomer, 1-HS-*syn*-B_18_H_21_**1**, shows two very similar polymorphic structures in which the sulfur atom also interacts with the bridging hydrogen atoms of two other molecules in such a way that it resembles wooden logs. The existence of different polymorphic structures can therefore be rationalized as a result of this specific interaction, -*S*(H)···μ-B*H*B-, between sulfur and some of the six similar bridging hydrogen atoms per molecule of each isomer. In addition to influencing the packing of molecules in their respective SC supramolecular structures, this interaction also explains the difference between the parent *syn*-B_18_H_22_ and all three thiolated isomers in the differential thermal analysis (Figure S30). While the parent *syn*-B_18_H_22_ shows complete loss of mass due to sublimation, all three thiol isomers sublime off at slightly higher temperature and only partly, 20–40%. The remaining 60–80% of the starting material turns into highly involatile blackish material. The lower volatility of the thiolated isomers compared to *syn*-B_18_H_22_ can be attributed particularly to this -*S*(H)···μ-B*H*B interaction. One related issue is the *HS-* hydrogen atom and its orientation, as the hydrogen is not directly involved in this structure-determining interaction between the sulfur of the HS group and the bridging hydrogen, μ-B*H*B, and is left to either exhibit a weak interaction with terminal BH vertices or be left to free conformation, which corresponds to computationally optimized minima.

### Luminescence Properties

**Figure 7:**
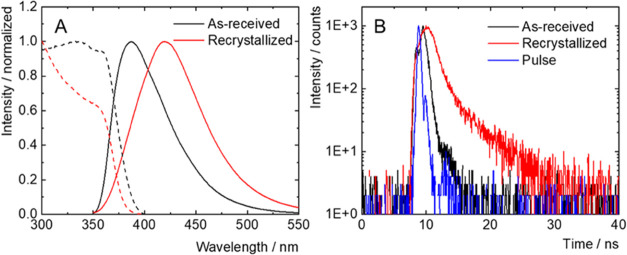
Normalized emission spectra (plain lines) of as-received *syn*-B_18_H_22_ (black) and recrystallized *syn*-B_18_H_22_ (red) excited at 380 nm in the air atmosphere; normalized excitation spectra (dashed lines) recorded at the maximum of emission (A). Fluorescence decay kinetics of *syn*-B_18_H_22_ in the air atmosphere, excited at 402 nm, recorded at the maximum of emission (B).

Borane compounds possess fascinating photophysical properties such as stimulated emission, thermochromism, or singlet oxygen photosensitization.^7,25,28,33,34,36,51^ We were therefore tempted to investigate the photophysical properties of the prepared borane compounds. In contrast to the remarkable fluorescence efficiency of *anti*-B_18_H_22_ in hexane (ϕ_L_ ∼ 1), its *syn*-B_18_H_22_ isomer has been reported to be nonemissive in solution.^29^ However, our measurement of the as-received powder of *syn*-B_18_H_22_, precursor to the thiolated boranes, showed an intense fluorescence in the solid state with a maximum at 385 nm, a quantum yield of 0.17, and an amplitude average lifetime of 0.4 ns (Figure 7 and Table 2). Interestingly, the recrystallization of *syn*-B_18_H_22_ from a diethyl ether/hexane mixture provided homostructural single crystals that displayed fluorescence with a maximum at 420 nm, a quantum yield of 0.20, and an amplitude average lifetime of 1.6 ns (Figure 7 and Table 2). For both samples, the excitation spectra were characterized by broad absorption bands in the UV-A region (Figure 7A). Thus, *syn*-B_18_H_22_ displays aggregation-induced fluorescence, and its photophysical parameters are microstructure-dependent. For comparison, solid *anti*-B_18_H_22_, recrystallized from a diethyl ether/hexane mixture, displayed a fluorescence band with maximum at 420 nm, an emission quantum yield of 0.78, and a lifetime of 6.5 ns, significantly higher than for *syn*-B_18_H_22_ (Figure S33 and Table 2).

**Table 2:** Photophysical Properties of *syn*-B_18_H_22_ and *anti*-B_18_H_22_ in the Solid State at Room Temperature^a^

sample	λ_L_ (nm)	ϕ_L_	τ_L_ (ns)
*syn*-B_18_H_22_^b^	385	0.17	0.4
*syn*-B_18_H_22_^c^	420	0.20	1.6
*anti*-B_18_H_22_^c^	420	0.78	6.5

a λ_*L*_, luminescence maximum (λ_exc_ = 340 nm); τ_L_, amplitude average lifetimes (λ_exc_ = 402 nm) measured at 420 nm; Φ_L_, luminescence quantum yields (λ_exc_ = 340 nm, experimental error of Φ_L_ is ±0.01).

b As-received.

c Recrystallized diethyl ether/hexane mixture.

**Figure 8:**
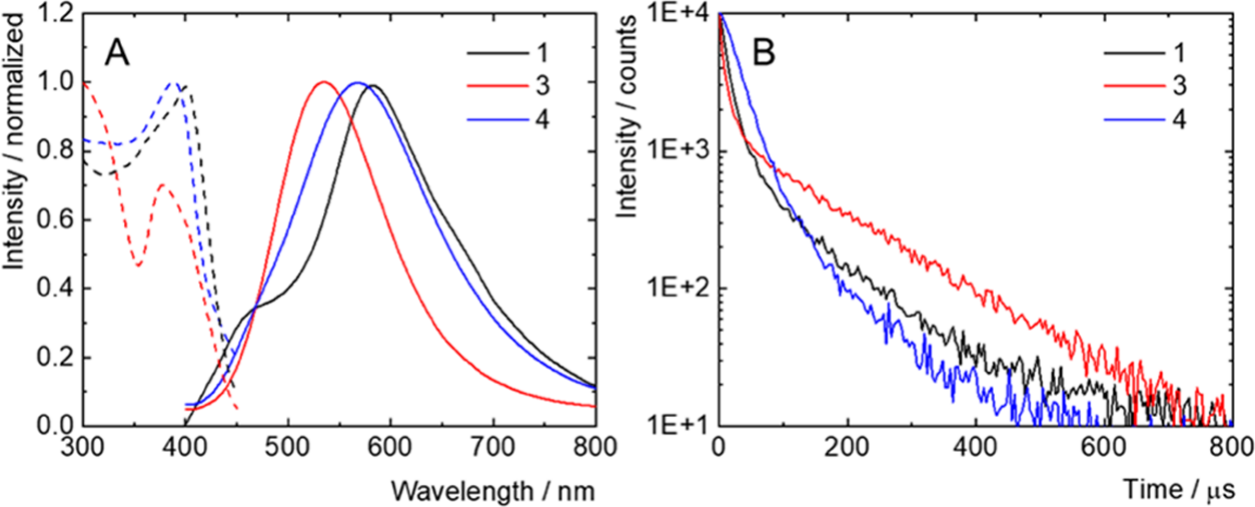
Normalized emission spectra (plain lines) of single crystals of **1**, **3**, and **4** excited at 380 nm in the air atmosphere; normalized excitation spectra (dashed lines) recorded at the maximum of emission (A). Phosphorescence decay kinetics of single crystals of **1**, **3**, and **4** in the air atmosphere, excited at 380 nm, recorded at the maximum of emission (B).

Single crystals of the thiolated boranes were measured on a single-crystal X-ray diffractometer to ensure their crystallographic purity, and they were then used for photophysical characterizations, the results of which are summarized in Table 3. Upon excitation at 380 nm, single crystals of **1**, **3**, and **4** displayed broad luminescence bands in the green/yellow region, with the respective maxima at 582, 535, and 570 nm (Figure 8). Interestingly, isomer **1** possessed a shoulder near its main emission band located in the blue region, which might be caused by additional emissive excited states. The excitation spectra recorded at the maximum of emission revealed broad absorption bands in the UV-A region (Figure 8A). Analysis of the luminescence decay kinetics recorded at the emission maximum evidenced amplitude average lifetimes of 14, 8.8, and 40 μs for isomers **1**, **3**, and **4**, respectively (Figure 8B). Thus, the thiolation of *syn*-B_18_H_22_ causes a bathochromic shift of the emission maximum and a decrease in luminescence efficiency. In addition, it leads to a switch from fluorescence to phosphorescence due to an increased intersystem crossing from singlet excited to triplet excited states, as already reported for iodinated or thiolated *anti*-B_18_H_22_ (Figure 8).^37,52^

**Table 3:** Photophysical Properties of Crystals of the Thiolated Boranes at Room Temperature^a^

sample	λL (nm)	ϕL	τL (μs)
1-HS-*syn*-B_18_H_21_	582, 465^b^		14, 5.2^b^
3-HS-*syn*-B_18_H_21_	535		8.8
4-HS-*syn*-B_18_H_21_	570	0.06	40
4-HS-*syn*-B_18_H_21_^c^	490	0.02	2.2

a λ_L_, luminescence maximum (λ_exc_ = 380 nm); τ_L_, amplitude average lifetimes (λ_exc_ = 380 nm) measured at the maximum of emission; Φ_L_, luminescence quantum yields (λ_exc_ = 380 nm, experimental error of Φ_L_ is ± 0.01).

b Shoulder.

c After grinding.

**Figure 9:**
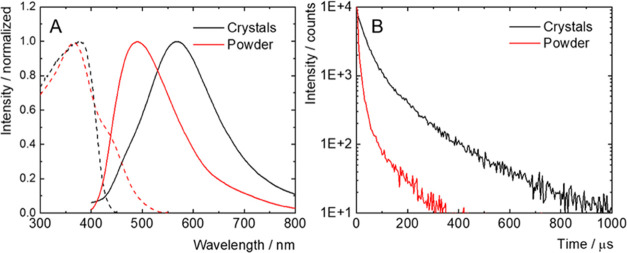
Normalized emission spectra (plain lines) of single crystals (black) and powder (red) of **4**, excited at 380 nm in an air atmosphere; normalized excitation spectra (dashed lines) recorded at the maximum of emission (A). Phosphorescence decay kinetics of single crystals (black) and powder (red) of **4** in an air atmosphere, excited at 380 nm, recorded at the maximum of emission (B).

Isomer **4** was obtained in sufficient quantity and crystallographic purity (Figure S38) to allow for a complete study of the luminescent properties as single crystals and the powdered sample resulting after grinding. Upon the grinding of the single crystals, a hypsochromic shift of the emission band was observed, with a shift of the luminescence maximum from 570 to 490 nm (Figure 9A). The corresponding emission quantum yield decreased from 0.06 to 0.02, and the emission amplitude average lifetime decreased from 40 to 2.2 μs (Figure 9B). Passing a flow of argon or oxygen through the powdered sample did not affect the emission lifetime, indicating the absence of quenching of the emissive triplet states by oxygen, a process often observed for phosphorescent dyes (Figure S31). It should be noted that this is the first report of a mechanochromic shift of the luminescence band of a borane cluster. Such a phenomenon has already been observed for phosphorescent copper-iodide complexes of the cubane-type, and it was attributed to a relaxation of the intramolecular distances upon grinding, thereby affecting the luminescent properties.^52^ In our case, the mechanochromic shift probably has a similar origin, as it is well known that the luminescent properties of borane compounds are dependent on their structure, which is affected by their environment.^52^ Finally, the luminescent properties of *syn*-B_18_H_22_ and the thiolated boranes were studied in hexane, where they showed very weak fluorescence (ϕ_L_ < 0.01), with maxima spanning from UV-A up to the green region of the spectrum and with a lifetime in the low nanosecond range (Table S4 and Figure S32). Overall, the strong dependency of the luminescent properties of these boranes on their environment allows for peculiar behaviors, which may further deepen the interest in this class of photoactive compounds.

### Self-Assembled Monolayers on Silver Surfaces, XPS Analysis

**Figure 10:**
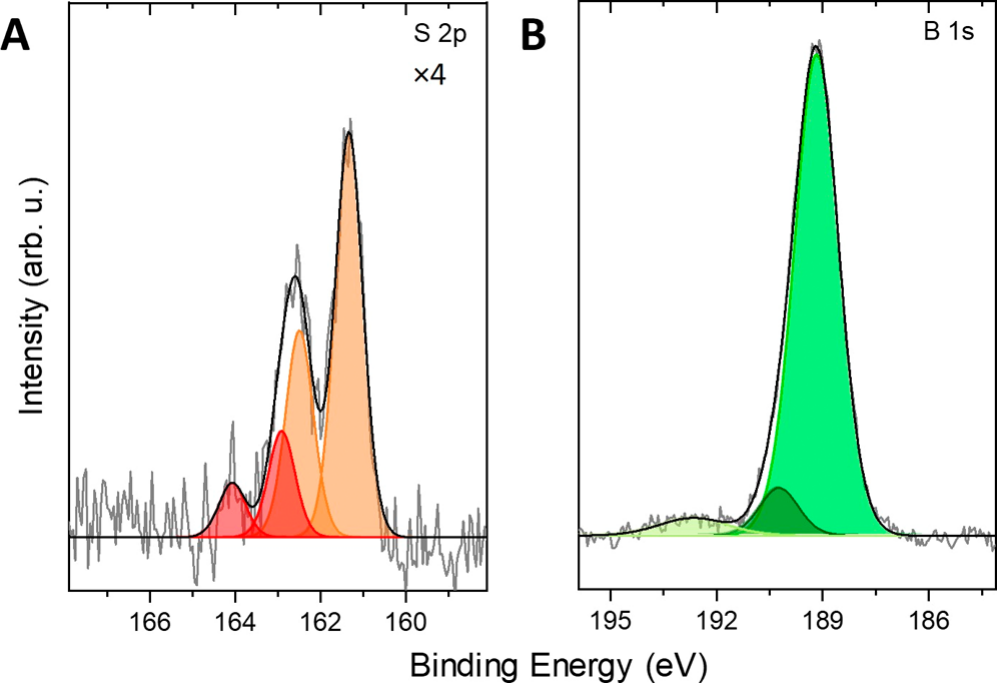
High-resolution S 2p (A) and B 1s (B) X-ray photoelectron spectra of the SAM formed by vacuum vapor deposition of isomer [4-HS-*syn*-B_18_H_21_] **4** on a Ag substrate. For better visualization, the S 2p spectrum is multiplied by a factor of 4.

Self-assembled monolayers (SAMs) of the isomer [4-HS-*syn*-B_18_H_21_] **4** on silver substrates were prepared by vapor deposition in ultra-high vacuum (UHV). The successful formation of these carbon-free SAMs was investigated using X-ray photoelectron spectroscopy (XPS). Figure 10A shows the high-resolution S 2p spectrum consisting of a main doublet (orange) with the binding energy (BE) of the S 2p_3/2_ and S 2p_1/2_ components at 161.3 and 162.5 eV, respectively, due to the formation of thiolates. The signal is accompanied by another doublet at the BE values of S 2p_3/2_ and S 2p_1/2_ at 162.9 and 164.1 eV, respectively (red). The formation of this additional sulfur signal can be attributed to the partial formation of disulfides during the self-assembly or to the presence of some physisorbed thiol molecules integrated into the monolayer *via* hydrogen bridge bonds. Both signals are shifted by ∼0.5 eV to lower BE values in comparison to the published values for boron-bound thiolates (typical values of S 2p_3/2_ at about 161.7 eV) and disulfides, or thiols, with the typical values of S 2p_3/2_ electrons at 163.0 eV.^53,54^ This indicates higher electron density on sulfur in [4-HS-*syn*-B_18_H_21_] **4**, as also evidenced by the value of the *HS*^1^H NMR chemical shift at about 1.4 ppm and indirectly also from the above-reported short-contact interactions with the acidic bridging hydrogen atoms.

**Figure 11:**
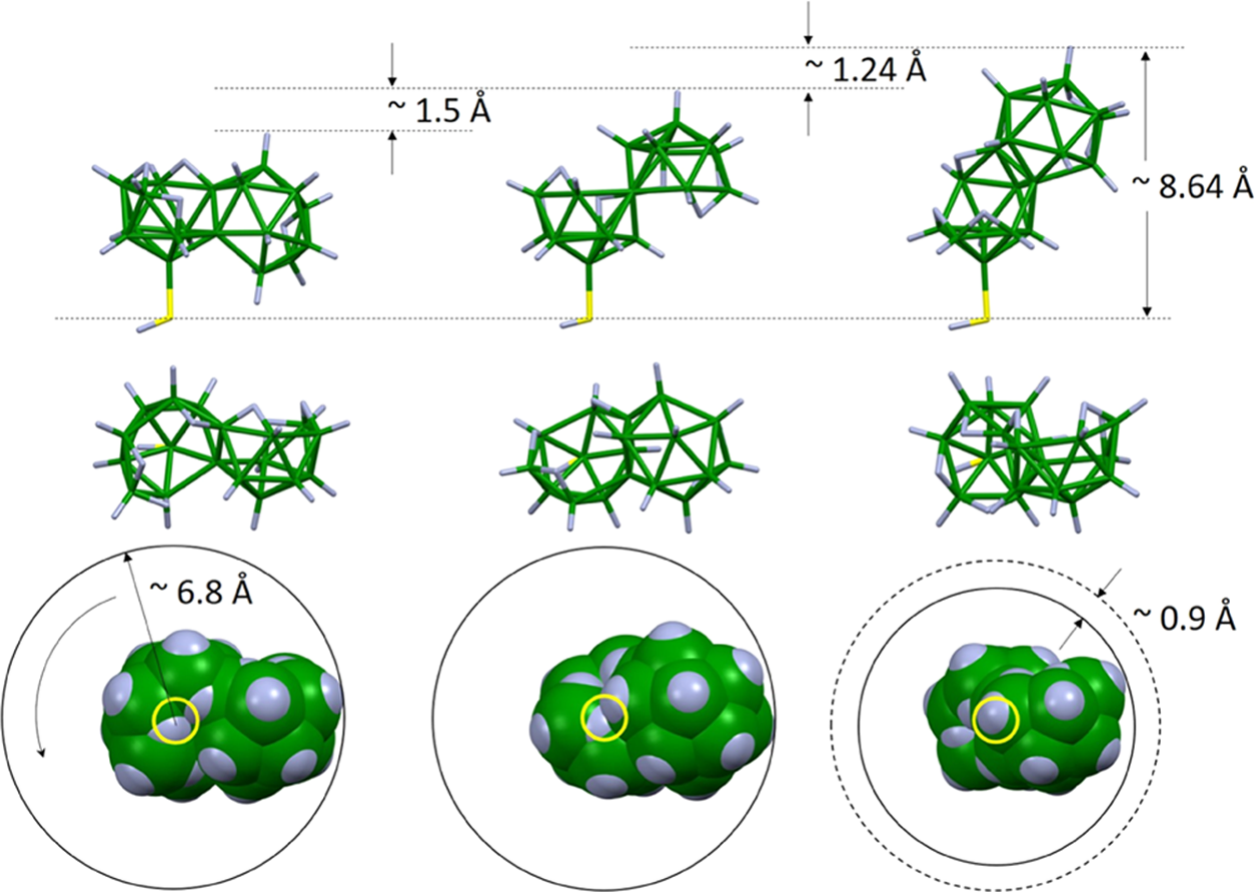
All three isomers are oriented with the HS group downward (top row), the respective top view (middle), and the space-filling projections (also top view). The yellow circle represents the position of the sulfur atom. Circles around the space-filling models show the lateral space requirement of the molecules rotating along their B–S axis.

In the B 1s spectrum, the main component (green) is found at a BE value of 189.1 eV with the full width at half-maximum of 1.5 eV, which corresponds to B–B bonds in the cluster (Figure 10B). This peak is accompanied by a shoulder at a BE of 190.2 eV (dark green), which we assign to the boron atom attached directly to sulfur in the B–S bonds. At 192.6 eV, a broad low-intensity peak due to aromatic shake-ups is visible. No other XP signals, with the exception of the metallic Ag substrate, were detected (see Figure S34) in the samples. Especially the absence of carbon and oxygen confirms the formation of high-quality, carbon-free SAM on the silver surface. The thickness was calculated to be 7 ± 2 Å and the B/S ratio to 19 ± 1:1, matching very well to the molecular structure and nominal stoichiometry of the isomer [4-HS-*syn*-B_18_H_21_] **4**. Figure 11 further shows the steric requirements (both lateral and longitudinal) of all three synthesized isomers and shows the theoretically enabled range in the subnanometer thickness of the respective SAMs.

## Conclusions

We have investigated the *syn*-B_18_H_22_ isomer, the “forgotten” isomer of the long-established B_18_H_22_ molecule.^12,15,24^ The more intensely studied *anti*-B_18_H_22_ isomer has been recently recognized for its lasing properties.^7^ Several previous studies^7,26^ stated that the *syn-*isomer is nonluminescent, and thus comparatively little attention has been paid to it over the last decade compared to *anti*-B_18_H_22_ boron hydride. In this study, we have demonstrated that not only does the parent compound have luminescence properties in its crystalline form but also that the HS-substituted isomers, (1-HS-*syn*-B_18_H_21_) **1**, (3-HS-*syn*-B_18_H_21_) **3**, and (4-HS-*syn*-B_18_H_21_) **4**, show luminescence both in their crystalline form and in solution. Within our systematic investigation of the HS-derivatives of *syn*-B_18_H_22_, we have aimed to use this molecule to reach our goal for the preparation of carbon-free self-assembled monolayers. Additionally, we have focused on monothiolated isomers of *syn*-B_18_H_22_ as constituents that enable us to adjust SAM thicknesses below 1 nm, depending on which vertex bears the HS group. In total, we have prepared five new crystal structures, as two of the isomers, isomers 1-HS-*syn*-B_18_H_21_ and 4-HS-*syn*-B_18_H_21_, were found to exist as two polymorphs. The packing of the molecules in the respective supramolecular structures of all of the isomers was commonly found to exhibit -*S*(H)···μB*H*B- interactions, and these give rise to the existence of different crystal polymorphs. We have also observed the substituent effect on the structure in comparison with the parent *syn*-B_18_H_22_ molecule. Changes of interboron distances adjacent to the substituent are too subtle to be apparent, but they become more obvious when we measure the effect on geometrical parameters effectively encompassing the whole molecule, as a whole, *i.e.*, on the distance between the centroids of the two subclusters of *syn*-B_18_H_22_, or on the distance between vertices from the opposite ends of the molecule, such as B(9)–B(9′). Such substituent metrics have also been estimated in terms of the overall cluster volume,^28,32^ and it is anticipated that *syn*-B_18_H_22_ with other substituents should also modify the photophysical properties. This study lays a solid foundation for further work in this direction.

## Experimental Section

### General Procedure and Instrumentation

All of the experiments were performed under an inert (argon, Ar) atmosphere using the standard Schlenk-line vacuum technique. Prior to use, all of the solvents were dried with sodium-benzophenone ketyl and distilled under an Ar atmosphere. Chloroform-d was purchased from ACROS Organic (Thermo Scientific Chemicals). Starting *syn*-B_18_H_22_ was purchased from Katchem s.r.o., Czech Republic, and used as received. Other chemicals, such as anhydrous aluminum trichloride and sulfur, were purchased from Fluka. The chromatographic separations were performed using a silica gel column. NMR spectra were recorded on a 600 MHz JEOL NMR spectrometer. Chemical shifts were analyzed with reference to the solvent (δ *=* 7.26 ppm, CDCl_3_, 600 MHz, 295K) and boron (δ *=* 0 ppm, relative to BF_3_(OEt)_2_) in CDCl_3_, 192.6 MHz, 295 K for the ^1^H and ^11^B{^1^H} NMR spectra, respectively. Mass spectrometry measurements were performed on a Thermo Scientific LCQ Fleet Ion Trap instrument using ESI with helium (5.0 Messer) as a collision gas in the ion trap. The sample was dissolved in acetonitrile (concentration ∼100 ng/ml) and introduced through a fused-silica sample tube of 0.100 mm (inner S-9 diameter) × 0.19 mm (outer diameter) to the ion source from a Hamilton syringe using infusion at 15 μL/min, a source voltage of 5.47 kV, a tube lens voltage of −44.71 V, a capillary voltage of −23.06 V, a capillary temperature of 165.01 °C, and N2 (isolated from air in NitroGen N1118LA, Peak Scientific) as a nebulizing sheath gas (flow rate 14.97 p.d.u.). Only negative ions of the respective molecular peaks were detected. IR spectra in the region from 4000 to 400 cm^–1^ (with a resolution of 4 cm^–1^) were measured using the Nexus 670 FT-IR firmy Thermo Electron Corporation with an ATR Smart iTX equipped with a diamond crystal. Total number of scans: 192, spectrometer detector: DTGS KBr, beam splitter: KBr. The thermal analysis of the samples was performed using Setsys Evolution 1750 (Setaram) equipped with an MS detector QMG 422 (Pfeiffer).

### Compound Synthesis

#### [1-HS-*syn*-B_18_H_21_] 1 (PM1a and PM1b), [3-HS-*syn*-B_18_H_21_] 3, and [4-HS-*syn*-B_18_H_21_] 4 (PM4a and PM4b)

Under an inert atmosphere of Ar, *syn*-B_18_H_22_ (4.4 g, 20 mmol), sulfur (1.25 g, 39 mmol), and AlCl_3_ (3.8 g, 29 mmol) were placed in a 500 mL round-bottom flask and heated with stirring to 125 °C. After 15 minutes of heating, the reaction mixture melted to form a brown liquid. The stirring continued for 6 h. The reaction mixture was then left to cool to room temperature, 250 mL of hexane was added, and then it was kept under an Ar atmosphere overnight. The reaction mixture was then cooled in an ice bath, and 50 mL of distilled water was added dropwise into the reaction mixture, followed by 10 mL of hydrochloric acid (10% v/v). The product was then gently stirred using a glass rod. The solution turned orange, with brownish solid particles at the bottom of the flask. The solution was filtered, and the filtrate was extracted with hexane (4 × 100 mL). The collected hexane fractions (slightly orange solution) were dried with MgSO_4_ and filtered, and the solvent was evaporated under reduced pressure on a rotary evaporator. All three isomers were then separated using column chromatography on silica gel with diethyl ether as the eluent. The yield was ∼50 mg of isomer 1-HS-*syn*-B_18_H_22_**1**, ∼30 mg of 3-HS-*syn*-B_18_H_22_**3**, and ∼230 mg 4-HS-*syn*-B_18_H_22_**4**.

### MS (ESI^+^)

*m*/*z* calculated for HS-B_18_H_21_ monocationic species: 248.3148 experimentally found 248.3152.

### NMR for [1-HS-*syn*-B_18_H_21_] 1

^11^B{^1^H} NMR (192.6 MHz, 295 K, CDCl_3_): B3 (16.7 ppm) > B3′ (15.0 ppm) > B1 (10.9 ppm) > B10′ (9.6 ppm) > B10 (8.3 ppm, according to the B10–B5 cross peak 8.6 ppm) > = B6 (8.3 ppm, according to the B6–B2,B2′ cross peak 8.3 ppm) >B 9′ (3.3 ppm) > B1′ (2.0 ppm) > B9 (1.0 ppm) > B8 (0.0 ppm) > B8′ (−2.0 ppm) >B 5 (−3.8 ppm) > B7 (−14.3 ppm) > B7′ (−16.6 ppm) > B2 (−28.1 ppm, according to the B2–B1 and B2–B3 cross peaks: −28.0 ppm on the *x*-axis, −27.9 on the *y*-axis) > = B2′ (−28.1 ppm, according to the B2′–B3′ and B1′–B2′ cross peaks: −28.4 ppm on the *x*-axis, −28.3 on the *y*-axis) > B4 (−37.2 ppm) > B4′ (−40.6 ppm).

### Crystal Data for PM1a

Empirical formula = B_18_ H_22_ S_1,_*M*_r_ = 248.8, monoclinic, space group *P*21/*c*, a = 10.0437 (6) Å, b = 11.3556 (6) Å, c = 13.8050 (8) Å, α = 90°, β = 108.575 (5)°, γ = 90°, *V* = 1492.47 (15) Å^3^, *Z* = 4, ρ_calc._ = 1.1073 g/cm^3^, μ = 1.536 mm^–1^, *F*(000) = 512, *R*1 = 0.0452, w*R*2 = 0.0944, 2952 independent reflections and 238 parameters (for more detail, see Table S8).

### Crystal Data for PM1b

Empirical formula = B_18_ H_22_ S_1,_*M*_r_ = 248.8, monoclinic, space group *P*21/*n*, a= 10.0415(7) Å, b = 11.6578(8) Å, c = 13.4184(9) Å, α = 90°, β = 110.018(6)°, γ = 90°, *V* = 1475.88(18) Å^3^, *Z* = 4, ρ_calc._ = 1.1198 g/cm^3^, μ = 1.553 mm^–1^, *F*(000) = 512, *R*1 = 0.0451, w**R**2 = 0.1115, 2867 independent reflections and 238 parameters (for more detail, see Table S9).

### NMR for [3-HS-*syn*-B_18_H_21_] 3

^11^B{^1^H} NMR (192.6 MHz, 295K CDCl_3_): B3 (21.4 ppm) > B3′ (13.5 ppm) > B10 (10.3 ppm) > B10′ (9.7 ppm) > B6 (6.6 ppm) > B1 (4.4 ppm) > B9′ (3.7 ppm) > B1′ (2.7 ppm) > B9 (0.8 ppm) > B8′ (−2.0 ppm according to COSY) > = B5 (−2.2 ppm) > B8 (−3.2 ppm) > B7′ (−15.4 ppm) > B7 (−16.8 ppm) > B2 (−28.6 ppm) > B2′ (−29.4 ppm) > B4 (−38.5 ppm) > B4′ (−39.4 ppm).

### Crystal Data

Empirical formula = B_18_ H_22_ S_1,_*M*_r_ = 248.8, monoclinic, space group *P*21/*c*, *a* = 12.264(2) Å, *b* = 6.659(2) Å, *c* = 19.098(4) Å, α = 90°, β = 107.06(3)°, γ = 90°, *V* = 1491.0(6) Å^3^, *Z* = 4, ρ_calc._ = 1.1084 g cm^–3^, μ = 1.538 mm^–1^, *F*(000) = 512, *R*1 = 0.0356, *w*R**2 = 0.0804, 2891 independent reflections and 238 parameters (for more detail, see Table S10).

### NMR for [4-HS-*syn*-B_18_H_21_] 4

^11^B{^1^H} NMR (192.6 MHz, 295 K, CDCl_3_): B3 (14.9 ppm) > B3′ (13.8 ppm) > B10 (10.3 ppm) > B10′ (9.1 ppm) > B6 (8.1 ppm) > B9′ (3.5 ppm) > B1 (3.0 ppm) > B9 (2.6 ppm) > B1′ (2.0 ppm) > B8 (−1.2 ppm) > B8′ (−1.6 ppm) > B5 (−3.4 ppm) > B7′ (−16.1 ppm) > = B7 (−16.2 ppm) > B2 (−27.7 ppm) > B2′ (−28.1 ppm) > B4 (−29.2 ppm) > B4′ (−39.8 ppm).

### Crystal Data for PM4a

Empirical formula = B_18_ H_22_ S_1,_*M*_r_ = 248.8, orthorhombic, space group *Pbca*, *a* = 14.4249 (5) Å, *b* = 11.9778 (4) Å, *c* = 17.8595 (6) Å, α = 90°, β = 90°, γ = 90°, *V* = 3085.74 (18) Å^3^, *Z* = 8, ρ_calc._ = 1.0712 g cm^–3^, μ = 1.486 mm^–1^, F(000) = 1024, *R*1 = 0.0300, w**R**2= 0.0720, 2994 independent reflections and 238 parameters (for more details, see Table S11).

### Crystal Data for PM4b

Empirical formula = B_18_ H_22_ S_1,_*M*_r_ = 248.8, monoclinic, space group *P*21/*n*, *a* = 10.453 (2) Å, *b* = 12.585 (3) Å, *c* = 11.985 (2) Å, α = 90°, β = 91.29 (3)°, γ=90°, *V* = 1576.2(5) Å^3^, *Z* = 4, ρ_calc._ = 1.0485 g cm^–3^, μ = 1.454 mm^–1^, *F*(000) = 512, *R*1 = 0.0355, w**R**2 = 0.0781, 3109 independent reflections and 238 parameters (for more details, see Table S13).

### Computational Study

Quantum chemistry calculations were performed with the Gaussian 16 package. The geometries were optimized by means of the density functional theory with the long-range-corrected functional wB97XD from Head-Gordon and co-workers, which includes empirical dispersion,^55^ using Ahlrichs′ triple-zeta set with polarization functions def2-TZVPP.^56^ A series of optimizations were performed in internal coordinates with the torsion angle between the thiol hydrogen atom and a selected neighbor of the substituted boron (2 for 1 and 3, 6 for 2, and 1 for 4) kept fixed at values reflecting the pentagonal arrangement around the substituted boron: 36, 108, 180, 252, and 324° (hydrogen between two neighboring borons) as well as 0, 72, 144, 216, and 288° (hydrogen above a neighboring boron); then the angle was relaxed, and a full optimization to a local minimum was conducted without constraints. NMR shielding was then calculated at the same level of theory using the GIAO method^57^ for the minima found (only two for each isomer), and their weighted averages with Boltzmann factors at 300 K for the weights were used to calculate the ^11^B chemical shifts, based on the shielding of diborane (chemical shift δ(^11^B) +16.6) calculated at the same level of theory in geometry optimized at the same level. Computationally obtained dipole moment values are summarized in the supporting information (Table S14 and Figure S47). The excited states were calculated within the frame of the time-dependent DFT (TD-DFT); the results are provided in the Supporting information file (Table S15 and Figures S48–S55)

### Single-Crystal X-ray Diffraction Analysis

Single crystals suitable for X-ray diffraction analysis of all three isomers were grown by slow evaporation of diethyl ether solutions at room temperature (18 °C). The X-ray diffraction data of the samples were collected with a Rigaku OD Supernova using an Atlas S2 CCD detector and a mirror-collimated Cu K_α_ (λ = 1.54184 Å) from a microfocused sealed X-ray tube. The samples were cooled to 95 K during the measurement. Integration of the CCD images, absorption correction, and scaling were done by the program CrysAlisPro 1.171.41.123a (Rigaku Oxford Diffraction, 2022). Crystal structures were solved by charge-flipping with the program SUPERFLIP^58^ and refined with the Jana2020 program package^59^ by full-matrix least-squares technique on *F*^2^. The crystal data collection and analysis details are reported in more detail in the Supporting Information.

### Luminescence Properties

Luminescence properties were analyzed on an FLS1000 spectrometer (Edinburgh Instruments, U.K.) using a cooled PMT-900 photon detection module (Edinburgh Instruments, U.K.). The FLS1000 spectrometer was also used for time-resolved luminescence measurements using, for excitation, a microsecond flash lamp (for **1**, **3**, and **4**) or a 402 nm EPL Series laser diode (for *syn*-B_18_H_22_). The recorded decay curves were fitted to exponential functions using the Fluoracle software (v. 2.13.2, Edinburgh Instruments, U.K.). Luminescence quantum yields were recorded using a Quantaurus QY C11347-1 spectrometer (Hamamatsu, Japan).

### Formation of Self-Assembled Monolayers (SAMs)

SAMs of [4-HS-*syn*-B_18_H_21_] **4** were prepared by evaporation in a UHV Multiprobe system (<2 × 10^–10^ mbar, Scienta Omicron) using a molecular evaporator (Kentax). The molecules were evaporated at 80 °C for 1 h on 300 nm silver on mica substrates (Georg Albert PVD), which were held at room temperature. The substrates were cleaned before by repeated sputtering with argon ions (1 keV, 10 mA, FDG15, Focus) and annealing at 370 °C.

### X-ray Photoelectron Spectroscopy (XPS)

XPS was measured in the same UHV system used for SAM formation with a monochromatic X-ray source (Al K_α_, 1486.7 eV) and an electron analyzer (Argus CU) with a spectral energy resolution of 0.6 eV. The XP spectra were calibrated by referencing the binding energy of the Ag 3d_5/2_ signal at 368.2 eV and fitted using Voigt functions (30:70) after linear background subtraction. Calculations of stoichiometry were performed with the software CasaXPS using the relative sensitivity factors of 1.68 (S 2p) and 0.49 (B 1s); the layer thickness was calculated using the Lambert–Beer equation. A mean free path of 27 Å was used for electrons that were released from the silver substrate and reached the detector through the SAM.^60^

## Supporting Information Available

Supporting information provides complete information on the NMR spectra (both experimental and computational), IR spectra, ESI mass spectra, results from thermal differential analysis, tables with structural data for all X-ray diffraction-determined isomers and polymorphs, analysis of supramolecular arrangement, more data on photophysical properties, X-ray photoelectron spectra of the respective SAM, and further computational data (PDF)
Optimized structure 1 (XYZ)
Optimized structure 2 (XYZ)
Optimized structure 3 (XYZ)
Optimized structure 4 (XYZ)
Optimized structure 5 (XYZ)

## Supplementary Material

ja3c05530_si_001.xyz

ja3c05530_si_002.xyz

ja3c05530_si_003.xyz

ja3c05530_si_004.xyz

ja3c05530_si_005.xyz
