# Supplementary material for: Macropolyhedral syn-B18H22, the “Forgotten” Isomer
Source: J Am Chem Soc. 2023 Aug 2;145(32):17975–86. doi: 10.1021/jacs.3c05530 (PMC10436279; doi:10.1021/jacs.3c05530)
Supplement: Supplementary file 6 — ja3c05530_si_006.pdf [file ja3c05530_si_006.pdf]

SUPPORTING INFORMATION

Macropolyhedral *syn*-$B_{18}H_{22}$, the "Forgotten" Isomer

Deepak Kumar Patel [a,b], B. S. Sooraj [a,b], Kaplan Kirakci[b], Jan Macháček [b], Monika Kučeráková [c], Jonathan Bould [b], Michal Dušek [c], Martha Frey [d], Christof Neumann [d], Sundargopal Ghosh [a*], Andrey Turchanin [d*], Thalappil Pradeep [a*], and Tomas Base [b*]

[a]*DST Unit of Nanoscience (DST UNS) and Thematic Unit of Excellence (TUE), Department of Chemistry, Indian Institute of Technology, Madras, Chennai – 600036, India. E-mail: pradeep@iitm.ac.in* , sghosh@iitm.ac.in

[b]*Institute of Inorganic Chemistry, The Czech Academy of Science, 25068 Rez, Czech Republic. E-mail: tbase@iic.cas.cz*

[c]*Institute of Physics, The Czech Academy of Science, 182 21 Prague 8, Czech Republic.*

[d]*Institute of Physical Chemistry Friedrich Schiller University Jena, 07743 Jena, Germany. E-mail: andrey.turchanin@uni-jena.de*

Keywords: Macropolyhedral, Boron hydride, *syn*-$B_{18}H_{22}$, Cluster, Luminescence, Phosphorescence, Self-assembled monolayers, Polymorphs

Contents

NMR

Figure S1.Experimental Stack plot of $^{11}B\{^{1}H\}$ decoupled spectra of *syn*-$B_{18}H_{22}$, 1-HS-*syn*-$B_{18}H_{21}$, 3-HS-*syn*-$B_{18}H_{21}$, and 4-HS-*syn*-$B_{18}H_{21}$.

Figure S2. Corrilartion of experimental coupled and decoupled $^{11}B\{^{1}H\}$ spectra of *syn*-$B_{18}H_{22}$, 1-HS-*syn*-$B_{18}H_{21}$, 3-HS-*syn*-$B_{18}H_{21}$, and 4-HS-*syn*-$B_{18}H_{21}$.

Figure S3. Experimental decoupled (top) and coupled (bottom) ^{11}B NMR spectra of *syn*-$B_{18}H_{22}$ with integrals normalised to 1 atom = 10 units.

Figure S4. Correlation between the (top) experimental and (bottom) computational ^{11}B NMR spectra of *syn*-$B_{18}H_{22}$. Ratio of the boron signal intensity of experimental ^{11}B NMR: 2:1:2:2:2:2:1:2:2:2.

Figure S5. 2-D [^{11}B-^{11}B]-COSY-$\{^{1}H\}$ NMR spectrum of *syn*-$B_{18}H_{22}$. with the 1D spectra aligned along its edges.

Figure S6. *syn*-$B_{18}H_{22}$ connections deduced from ^{11}B-COSY NMR

Figure S7. *syn*-$B_{18}H_{22}$ connections deduced from ^{11}B COSY NMR: a) augmented with hydrogen bridges found in ^{11}B–^{1}H HMQC NMR; b) expanded to the full formula.

Figure S8. 2-D [^{1}H-^{11}B]-HMQC NMR spectrum of *syn*-$B_{18}H_{22}$ with the 1D spectra aligned along its edges.

Figure S9. $^{1}H\{B\}$ decoupled (top) and coupled (bottom) proton NMR spectra of *syn*-$B_{18}H_{22}$

Figure S10. Experimental decoupled (top) and coupled (bottom) ^{11}B NMR spectra of 1-HS-*syn*-$B_{18}H_{21}$ (**1**).

Figure S11. Correlation between the (top) experimental and (bottom) computational ^{11}B NMR spectra of 1-HS-*syn*-$B_{18}H_{21}$ (**1**). Ratio of the boron signal intensity of experimental ^{11}B NMR: 1:1:1:1:2:1:1:1:1:1:1:1:1:2:1:1

Figure S12. 2-D [^{11}B-^{11}B]-COSY-$\{^{1}H\}$ NMR spectrum of 1-HS-*syn*-$B_{18}H_{21}$ (**1**).

Figure S13: ^{11}B COSY NMR spectrum of 1-HS-*syn*-$B_{18}H_{21}$ treated to see as many cross peaks as possible.

Figure S14. 2-D [^{1}H-^{11}B]-HMQC NMR spectrum of 1-HS-*syn*-$B_{18}H_{21}$ (**1**).

Figure S15. $^{1}H\{B\}$ decoupled (top) and coupled (bottom) proton NMR spectra of 1-HS-*syn*-$B_{18}H_{21}$ (**1**)

Figure S16. Experimental decoupled (top) and coupled (bottom) ^{11}B NMR spectra of 3-HS-*syn*-$B_{18}H_{21}$ (**3**).

Figure S17. Correlation between the (top) experimental and (bottom) computational ^{11}B NMR spectra of 3-HS-*syn*-$B_{18}H_{21}$ (**3**). Ratio of the boron signal intensity of experimental ^{11}B NMR: 1:1:1:1:1:1:1:2:1:1:1:1:1:1:1:1:1.

Figure S18. 2-D [^{11}B-^{11}B]-COSY-$\{^{1}H\}$ NMR spectrum of 3-HS-*syn*-$B_{18}H_{21}$ (**3**).

Figure S19. 2-D [^{1}H-^{11}B]-HMQC NMR spectrum of 3-HS-*syn*-$B_{18}H_{21}$ (**3**).

Figure S20. $^{1}H\{B\}$ decoupled (top) and coupled (bottom) proton NMR spectra of 3-HS-*syn*-$B_{18}H_{21}$ (**3**).

Figure S21: ^{1}H–^{11}B HMQC NMR spectrum of 3-HS-syn-$B_{18}H_{21}$ treated to resolve the cross peaks of B(2) with H(2) and B(2') with H(2')

Figure S22. Experimental decoupled (top) and coupled (bottom) ^{11}B NMR spectra of 4-HS-*syn*-$B_{18}H_{21}$.

Figure S23:^{1}H decoupled ^{11}B NMR spectrum of 4-HS-*syn*-$B_{18}H_{21}$ superimposed on the non-decoupled one, transformation of the FID fine-tuned to get lines narrow enough for discriminating all the signals.

Figure S24. Correlation between the (top) experimental and (bottom) computational ^{11}B NMR spectra of 4-HS-*syn*-$B_{18}H_{21}$ (**4**). Ratio of the boron signal intensity of experimental ^{11}B NMR: 1:1:1:1:1:1:2:1:2:1:2:1:1:1:1

Figure S25. 2-D [^{11}B-^{11}B]-COSY-$\{^{1}H\}$ NMR spectrum of 4-HS-*syn*-$B_{18}H_{21}$ (**4**)

Figure S26. 2-D [^{1}H-^{11}B]-HMQC NMR spectrum of 4-HS-*syn*-$B_{18}H_{21}$ (**4**)

Figure S27. Decoupled (top) $^1H\{B\}$ and coupled (bottom) proton NMR spectra of 4-HS-*syn*-$B_{18}H_{21}$ (**4**).

Table S1: NMR chemical shifts of ^{11}B and ^1H in *syn*-$B_{18}H_{22}$

Table S2. Calculated chemical shift in ppm

Table S3. Experimental chemical shift in ppm

MS

Figure S28. Positive ion mode MS ESI spectra of HS-$B_{18}H_{21}$. (Top) full range m/z spectrum and (bottom) corresponding experimental and theoretical matching of different fragments.

IR

Figure S29. FTIR of HS-*syn*-$B_{18}H_{21}$ isomers and *syn*-$B_{18}H_{22}$.

TGA

Figure S30. DT analysis showing the volatility of HS-*syn*-$B_{18}H_{21}$ isomers.

Photophysical Properties

Figure S31. Phosphorescence decay kinetics of powder of 4-HS-*syn*-$B_{18}H_{21}$ in air atmosphere (black), argon atmosphere (red) or oxygen atmosphere (blue) excited at 380 nm, recorded at 470 nm.

Figure S32. Normalized emission spectra of *syn*-$B_{18}H_{22}$, **1**, **3**, and **4** in air-saturated hexane, excited at 330 nm; normalized excitation spectra (dashed lines) recorded at the maximum of emission, apart for *syn*-$B_{18}H_{22}$ recorded at 420 nm (A). Fluorescence decay kinetics of *syn*-$B_{18}H_{22}$, **1**, **3**, and **4** in air-saturated hexane, excited at 402 nm, recorded at the maximum of emission, apart for *syn*-$B_{18}H_{22}$ recorded at 420 nm (B).

Figure S33. Normalized emission spectra (plain lines) of *anti*-$B_{18}H_{22}$ excited at 340 nm in air atmosphere; normalized excitation spectra (dashed lines) recorded at the maximum of emission **(A)**. Fluorescence decay kinetics of *anti*-$B_{18}H_{22}$ in air atmosphere, excited at 402 nm, recorded at 420 nm **(B)**.

Table S4. Photophysical properties of *syn*-$B_{18}H_{22}$ and the thiolated boranes in hexane at room temperature.[a]

XPS

Figure S34. Overview X-ray photoelectron spectrum (A) as well as high-resolution C 1s (B) and O 1s (C) spectra of the formed [4-HS-*syn*-$B_{18}H_{21}$] **4** SAM on Ag. The data clearly show only the presence of boron, sulfur and silver signals in the sample. No carbon or oxygen signals are detected demonstrating a high quality of the formed SAM.

X-ray diffraction analysis

Figure S35**.** B-B-B angle, bond and centroid(c1-c2) distance of syn-$B_{18}H_{22,}$ **1**, **3**, and **4** ismores polymorphs (A, B, C, D, E, and F respectively).

Figure S36. Rietveld refinement of the powdered sample of *syn*-$B_{18}H_{22}$.

Figure S37. Rietveld refinement of the powdered sample of1-HS-*syn*-$B_{18}H_{21}$ (PM1b) and *syn*-$B_{18}H_{22}$.

Figure S38. Rietveld refinement of the powdered sample of 4-HS-*syn*-$B_{18}H_{21}$ (PM4a).

Figure S39. Rietveld refinement of the powdered sample of 4-HS-*syn*-$B_{18}H_{21}$ (PM4a and PM4b) and *syn*-$B_{18}H_{22.}$

Figure 40. Powder X-ray diffraction pattern (PXRD) of *anti*-$B_{18}H_{22}$ (obtained at room temperature using a PANalytical Empyrean diffractometer equipped with a conventional Cu X-ray tube and PIXcel detector). The data were processed using the HighScore Plus program from PANalytical, and the obtained diffraction pattern fits perfectly to the single-crystal simulated one.

Figure S41. ORTEP structure of *syn*-$B_{18}H_{22}$ isomer having 50% thermal ellipsoid probability.

Figure S42. ORTEP structure of 1-HS-*syn*-$B_{18}H_{21}$ PM1a isomer having 50% thermal ellipsoid probability.

Figure S43. ORTEP structure of 1-HS-*syn*-$B_{18}H_{21}$ PM1b isomer having 50% thermal ellipsoid probability.

Figure S44. ORTEP structure of 3-HS-*syn*-$B_{18}H_{21}$ isomer having 50% thermal ellipsoid probability.

Figure S45. ORTEP structure of 4-HS-*syn*-$B_{18}H_{21}$ PM4a isomer having 50% thermal ellipsoid probability.

Figure S46. ORTEP structure of 4-HS-*syn*-$B_{18}H_{21}$ PM4b isomer having 50% thermal ellipsoid probability.

Table S5. Bond distance comparison of all the isomers with *syn*-$B_{18}H_{22}$

Table S6. Crystallographic collection and refinement data for *syn*-$B_{18}H_{22}$ measured at 95 K.

Table S7. Crystallographic collection and refinement data for *syn*-$B_{18}H_{22}$ measured at room temperature.

Table S8. Crystallographic collection and refinement data for 1-HS-*syn*-$B_{18}H_{21}$ (PM1a).

Table S9. Crystallographic collection and refinement data for 1-HS-*syn*-$B_{18}H_{21}$ (PM1b).

Table S10. Crystallographic collection and refinement data for 3-HS-*syn*-$B_{18}H_{21}$.

Table S11. Crystallographic collection and refinement data for 4-HS-*syn*-$B_{18}H_{21}$ (PM 4a).

Table S12. Crystallographic collection and refinement data for PM4a measured at room temperature.

Table S13. Crystallographic collection and refinement data for 4-HS-*syn*-$B_{18}H_{21}$ (PM4b).

Dipole moment

Figure S47. Graphical projection of the dipole moment vectors in the SH-$B_{18}H_{21}$ isomers as well as in the parent *syn*-$B_{18}H_{22}$ and *anti*-$B_{18}H_{22}$.

Table S14. Table of the computationally obtained dipole moment values of all isomers and the parent *syn*-$B_{18}H_{22}$.

TD-DFT

Figure S48: Simulated spectra (A, D), excited states (B, E) and excited states in the relaxed geometry of S1 (C, F) of *anti* (A-C) and *syn* (D-F) $B_{18}H_{22}$; in the excited states diagrams, singlets are depicted yellow, triplets orange, and the length of the yellow lines on the left side, with scale labels in nm, represents the relative oscillator strengths of the singlet excited states.

Figure S49: Simulated spectra (A), excited states (B) and excited states in the relaxed geometry of S1 (C) of 1-HS-*syn*-$B_{18}H_{21}$.

Figure S50: Simulated spectra (A), excited states (B) and excited states in the relaxed geometry of S1 (C) of 2-HS-*syn*-$B_{18}H_{21}$.

Figure S51: Simulated spectra (A), excited states (B) and excited states in the relaxed geometry of S1 (C) of 3-HS-*syn*-$B_{18}H_{21}$.

Figure S52: Simulated spectra (A), excited states (B) and excited states in the relaxed geometry of S1 (C) of 4-HS-syn-$B_{18}H_{21}$.

Figure S53: Optimized geometries of *anti*-$B_{18}H_{22}$: the ground state (A) and the first excited singlet (B).

Figure S54: Optimized geometries of *syn*-$B_{18}H_{22}$: the ground state (A) and the first excited singlet (B).

Figure S55: Natural Transition Orbitals (bottom hole, top electron) for the absorption of *syn*-$B_{18}H_{22}$ to the first excited singlet in the ground state geometry (left) and its luminescence from the relaxed geometry of S1 (right).

Table S15: Excitation energies (E_{exc}) and the associated wavelengths (λ) of the first ((S1) and second (S2) excited states in the ground state geometry, and that of the first singlet excited state in its optimised geometry (S1/S1)

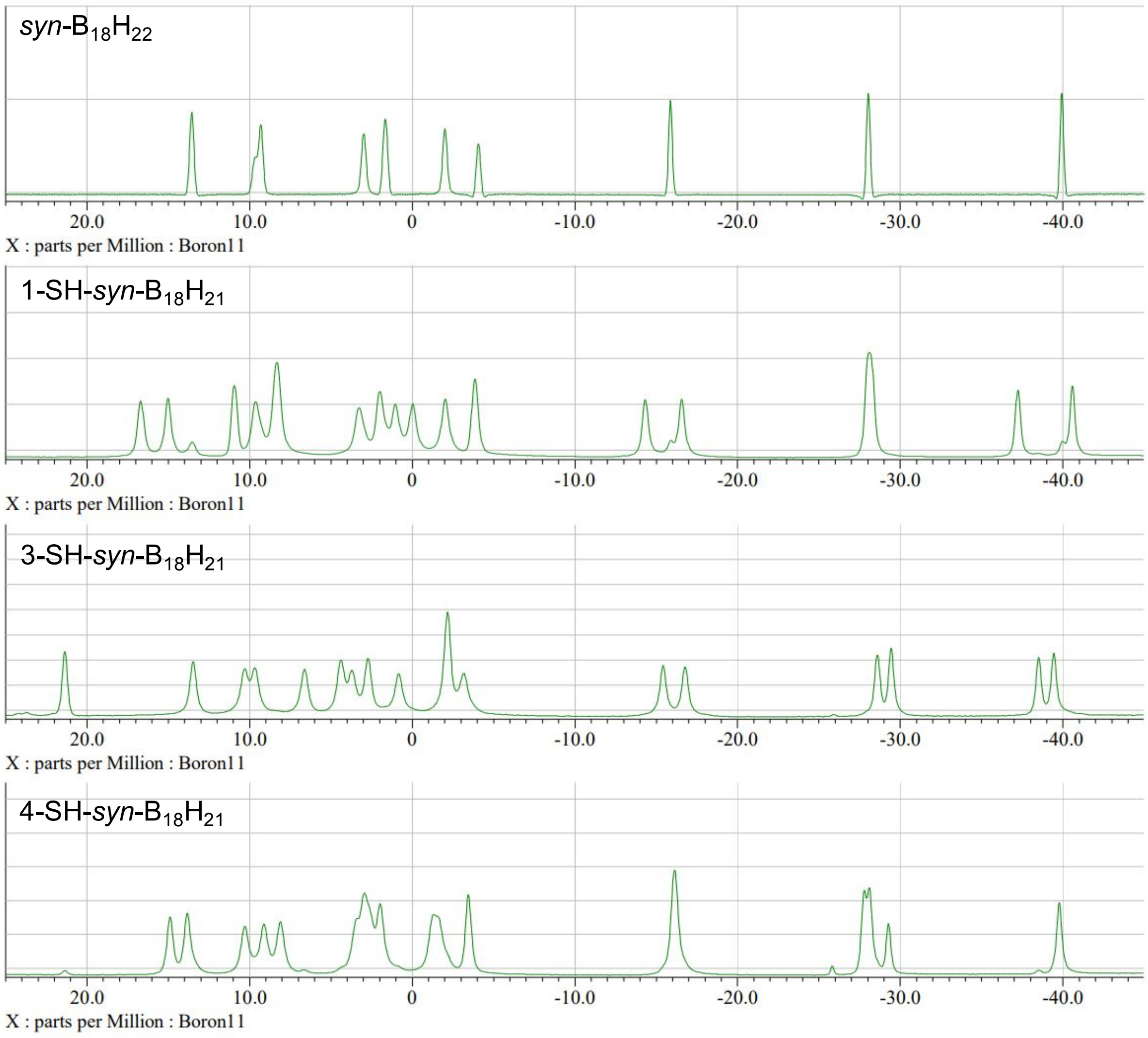

Figure S1. Experimental Stack plot of $^{11}B\{^{1}H\}$ decoupled spectra of *syn*-$B_{18}H_{22}$, 1-HS-*syn*-$B_{18}H_{21}$, 3-HS-*syn*-$B_{18}H_{21}$, and 4-HS-*syn*-$B_{18}H_{21}$.

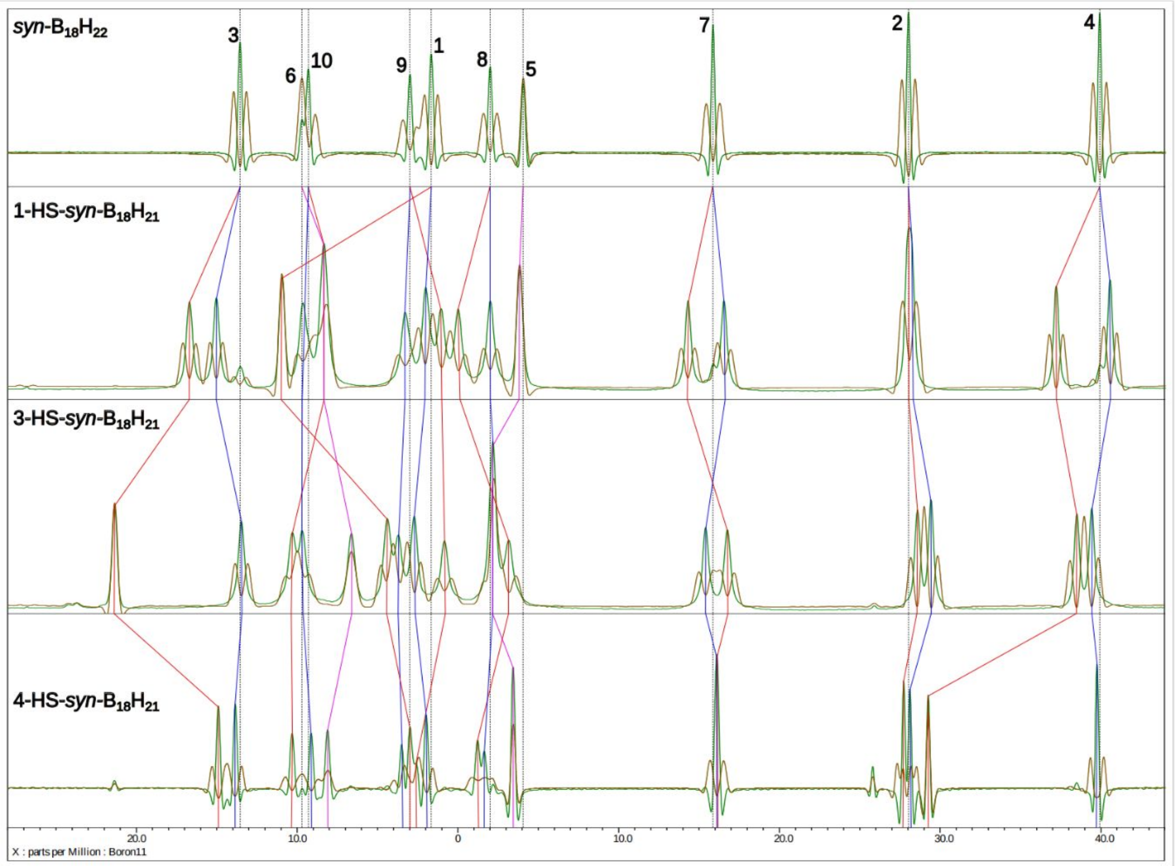

Figure S2. Corrilartion of experimental coupled and decoupled $^{11}B\{^{1}H\}$ spectra of *syn*-$B_{18}H_{22}$, 1-HS-*syn*-$B_{18}H_{21}$, 3-HS-*syn*-$B_{18}H_{21}$, and 4-HS-*syn*-$B_{18}H_{21}$.

syn-$B_{18}H_{21}$ ^{11}B NMR assignment

In the ^{11}B NMR spectrum of *syn*-$B_{18}H_{22}$ (Figure S3) we see ten peaks: 13.5 ppm (a), 9.7 ppm (b), 9.3 ppm (c), 3.0 ppm (d), 1.7 ppm (e), -2.0 ppm (f), -4.1 ppm (g), -15.9 (h), -28.0 (i), and -40.0 (j); b and g are singlets of intensity 1, and all others doublets of intensity 2, so b and g evidently belong to the two common atoms of the two decaborane subunits, 5 and 6.

The ^{11}B COSY NMR spectrum (Figure S5) allows to deduce information on the connectivity of the boron atoms represented by individual signals. The signal a has cross peaks with e, f, h, i, and j; b with i, and possibly a weak one with h; c with e, g, and j; d only with j; e with a, c, g, i, and j; f with a, h, and j; g with c, e, and i; h with a, f, i, and possibly b; i with a, b, e, g, and h; and j with a, c, d, e, and f. So a will be a neighbour of e, and they will have two common neighbours, i and j, which suggests the arrangement of two triangles a–e–i and a–e–j with a–e for a common edge; a and i have h for another common neighbour besides e, a and j form another triangle with f, e

and i with g, and e and j with c, while h and f also interact with one another, and so do c and g; we can connect d on the basis of the COSY only to j, and b to i with a possible but uncertain completion of a triangle with h (Figure S6).

The ^{11}B–^{1}H HMQC spectrum (Figure S8) allows assignment of protons to borons. To identify the correct position of our connection diagram in the schematic structure of *syn*-$B_{18}H_{22}$ (Figure 1B), we will need, especially for the bridging hydrogens, the information which pairs of boron atoms are connected by them. Among our ^{11}B signals, a has a cross peak with ^{1}H at 3.8 ppm, b at 1.3 ppm, and we know for two reasons that this is a bridging hydrogen: b is a singlet, so it has no terminal hydrogen, and the hydrogen at 1.3 has another cross peak with h; at the same time, h has another cross peak with ^{1}H, its terminal hydrogen, at 2.6 ppm. For c, there is a cross peak with -0.9 ppm with another bridging hydrogen that interacts with d as well, and a weak cross peak with its terminal ^{1}H at 3.8 ppm. The cross peak with the terminal ^{1}H of d is at 3.4 ppm, and there is one more cross peak of d with one more bridging hydrogen at -2.5 ppm; that bridging hydrogen interacts with f. That boron has the cross peak with its terminal hydrogen at 3.1 ppm, while e has it at 3.6 ppm, i at -0.2 ppm, j at 0.6 ppm, and g, the second singlet in ^{11}B spectrum, has none. The important part for ^{11}B NMR assignment are the three bridging hydrogens that connect b with h, c with d, and d with f: when we add these hydrogen bridges to our connection diagram, we can finally fit it on the *syn*-$B_{18}H_{22}$ structure unequivocally (Figure S7).

From the comparison of Figure S7 and Figure 1B follows that a (13.5 ppm) = 3, 3'; b (9.7 ppm) = 6 (= 6'); c (9.3 ppm) = 10, 10'; d (3.0 ppm) = 9, 9'; e (1.7 ppm) = 1, 1'; f (-2.0 ppm) = 8, 8'; g (-4.1 ppm) = 5 (= 5'); h (-15.9 ppm) = 7, 7'; i (-28.0 ppm) = 2, 2'; and j (-40.0 ppm) = 4, 4'.

Table S1: NMR chemical shifts of ^{11}B and ^{1}H in *syn*-$B_{18}H_{22}$

positions	1, 1'	2, 2'	3, 3'	4, 4'	5 = 5'	6 = 6'	7, 7'	8, 8'	9, 9'	10, 10'	μ(6, 7)	μ(8, 9)	μ(9, 10)
δ(^{11}B)/ppm	1.7	-28.0	13.5	-40.0	-4.1	9.7	-15.9	-2.0	3.0	9.3	X	X	X
δ(^{1}H)/ppm	3.6	-0.2	3.8	0.6	X	X	2.6	3.1	3.4	3.8	1.3	-2.5	-0.9

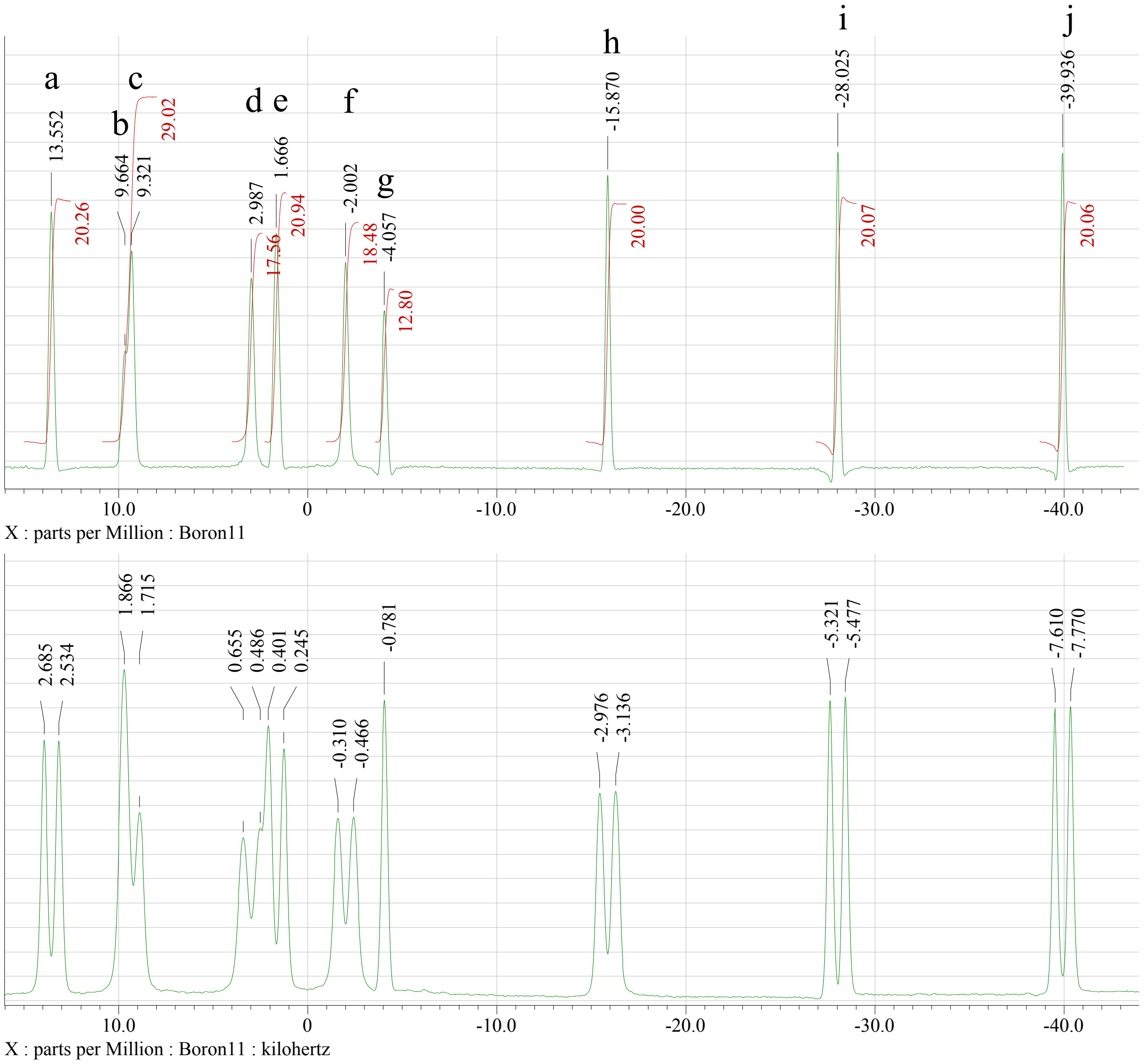

Figure S3. Experimental decoupled (top) and coupled (bottom) ^{11}B NMR spectra of *syn*-$B_{18}H_{22}$ with integrals normalised to 1 atom = 10 units.

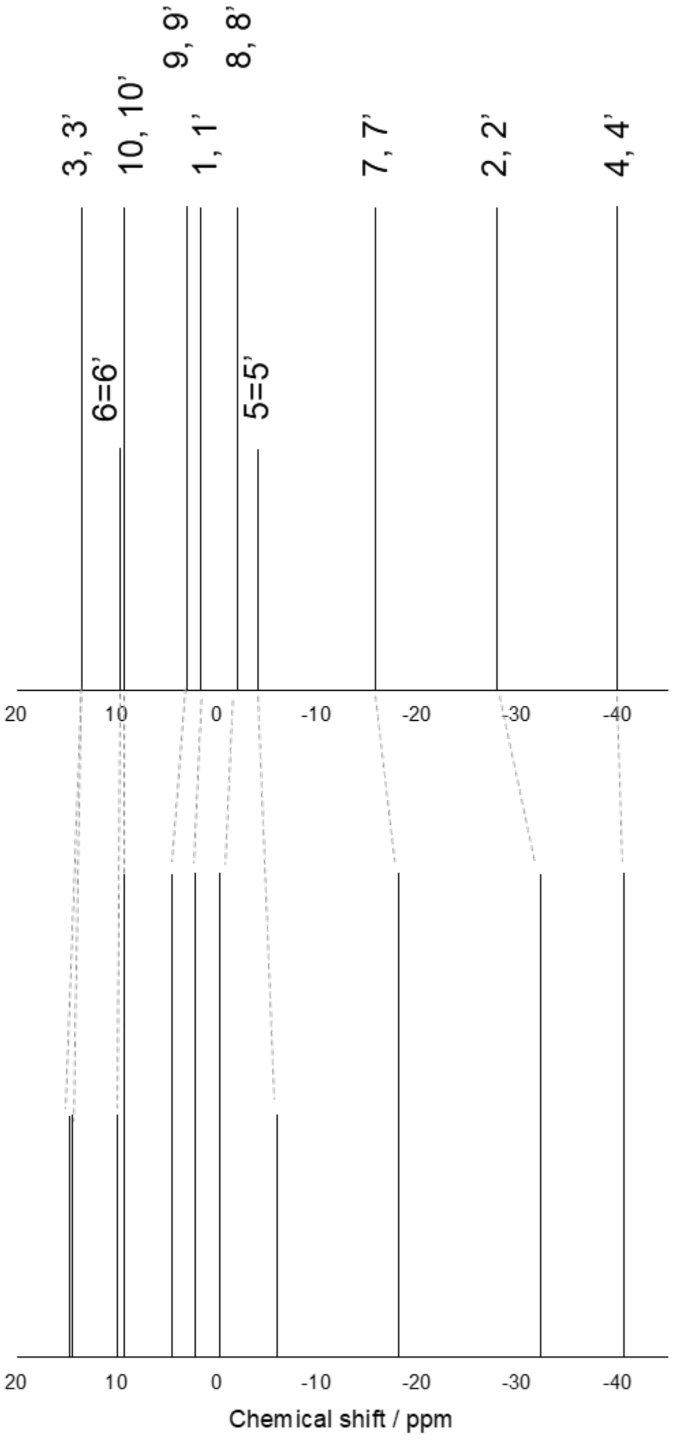

Figure S4. Correlation between the (top) experimental and (bottom) computational ^{11}B NMR spectra of *syn*-$B_{18}H_{22}$. Ratio of the boron signal intensity of experimental ^{11}B NMR: 2:1:2:2:2:2:1:2:2:2.

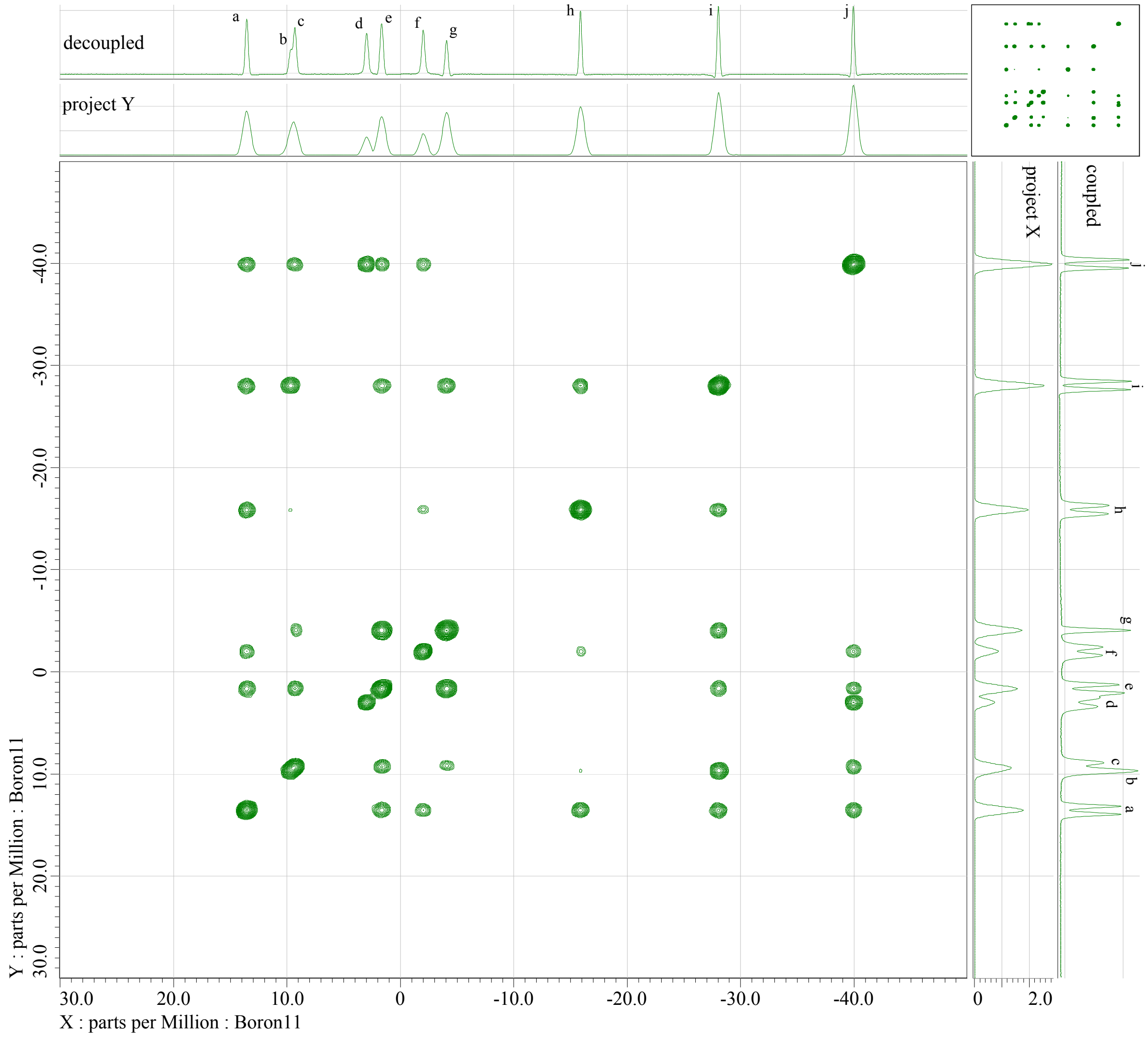

Figure S5. 2-D [^{11}B-^{11}B]-COSY-{^{1}H} NMR spectrum of *syn*-$B_{18}H_{22}$ with the 1D spectra aligned along its edges.

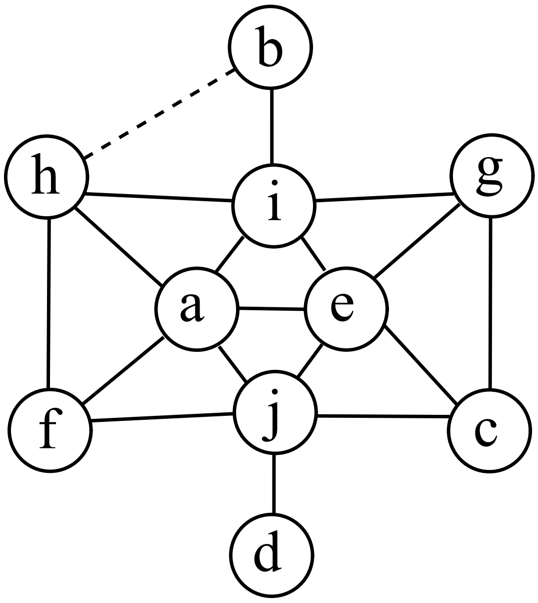

Figure S6. *syn*-$B_{18}H_{22}$ connections deduced from ^{11}B-COSY NMR

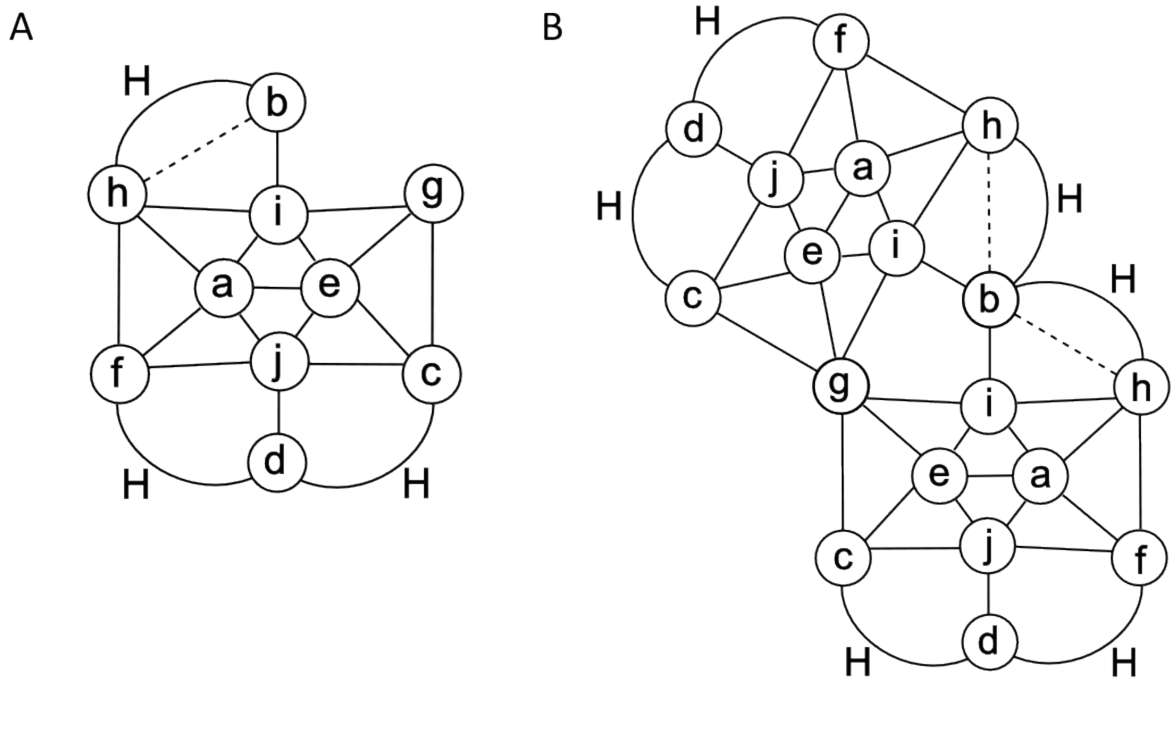

Figure S7. *syn*-$B_{18}H_{22}$ connections deduced from ^{11}B COSY NMR: (A) augmented with hydrogen bridges found in ^{11}B–^{1}H HMQC NMR; (B) expanded to the full formula.

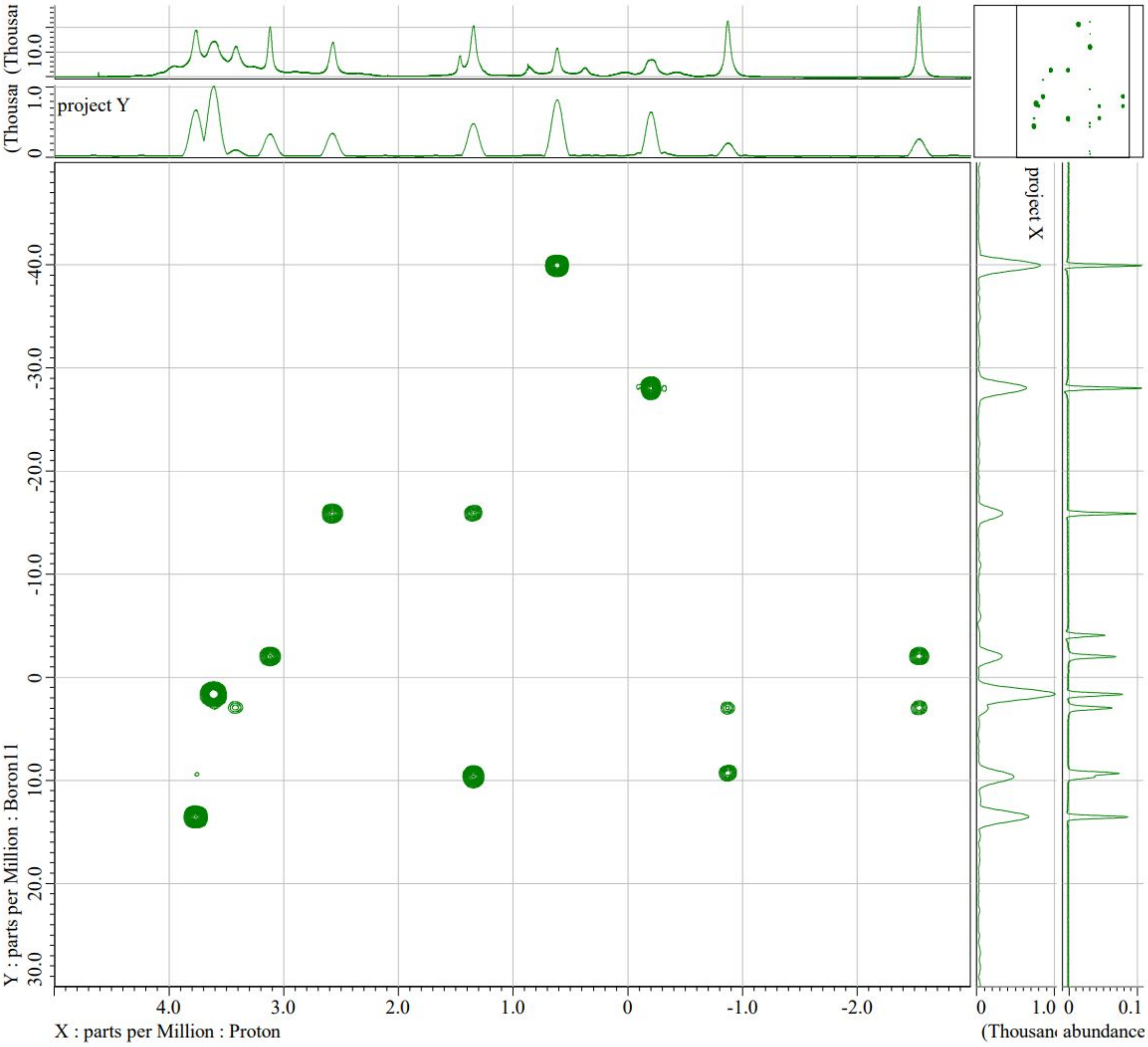

Figure S8. 2-D [^{1}H-^{11}B]-HMQC NMR spectrum of *syn*-$B_{18}H_{22}$ with the 1D spectra aligned along its edges.

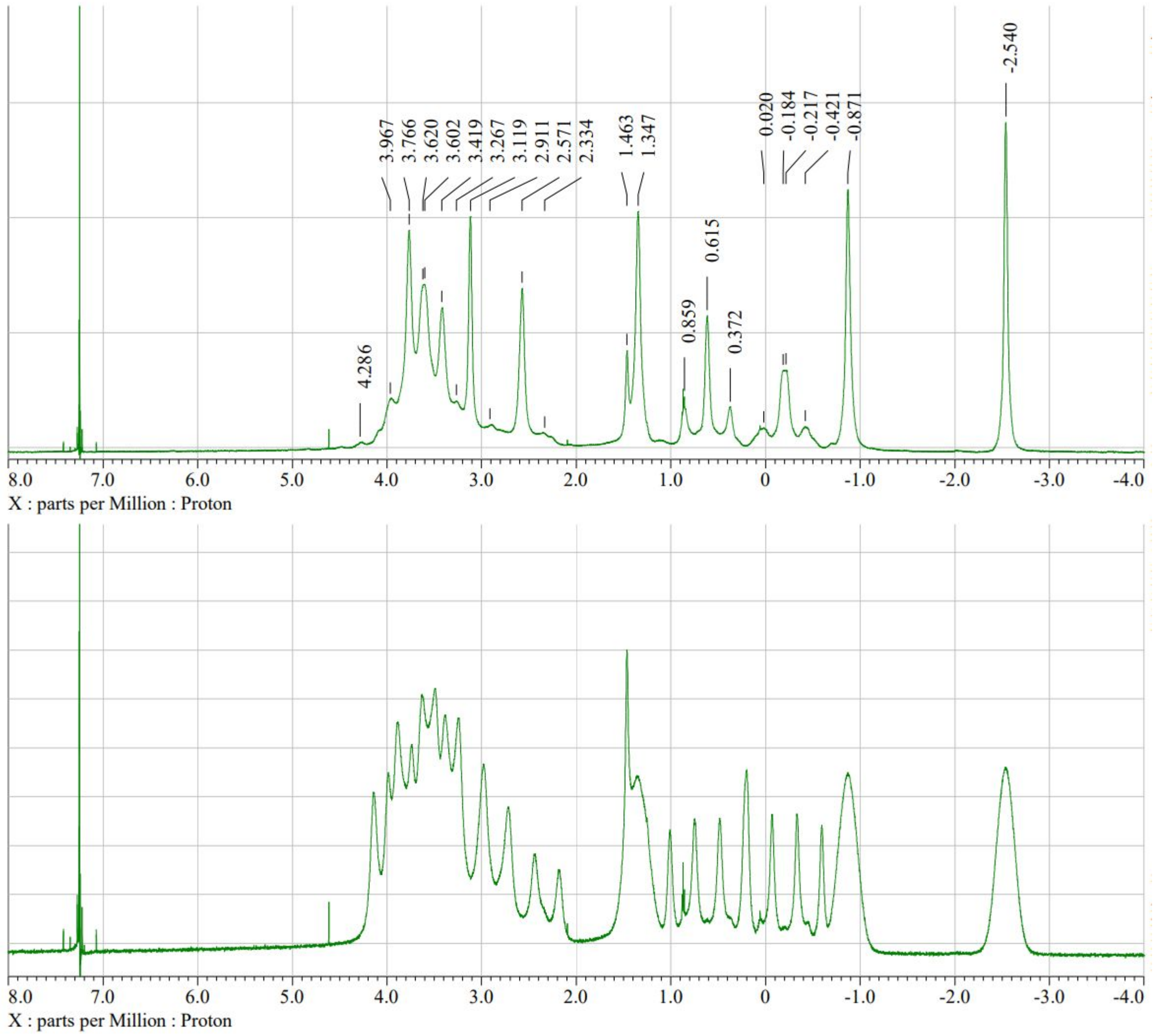

Figure S9. $^{1}H\{B\}$ decoupled (top) and coupled (bottom) proton NMR spectra of *syn*-$B_{18}H_{22}$

1-HS-*syn*-$B_{18}H_{21}$ ^{11}B NMR assignment

In the ^{11}B NMR spectra of monosubstituted *syn*-$B_{18}H_{22}$ derivatives we can expect three singlets: the peaks of B(5) and B(6), and that of the substituted atom. In the spectra of 1-HS-*syn*-$B_{18}H_{21}$ (Figure S10) we see clearly two singlets at 11.0 ppm and -3.8 ppm, altogether 16 major peaks, and three smaller peaks of a major impurity (10–20 molar %) that correspond to three peaks (of intensity 2) of the parent *syn*-$B_{18}H_{22}$. The peaks at 8.3 ppm and -28.1 ppm have integrated intensity two times larger than the other 14, and that at 8.3 ppm contains the third singlet, coinciding with a doublet. Comparing the spectra of the derivative with those of the parent borane, we can tentatively assign the pair of peaks in the lowest field, at 16.7 ppm and 15.0 ppm, to the boron atoms 3 and 3', that in the highest field, at -37.2 and -40.6 ppm, to the borons 4 and 4', the peaks coinciding at -28.0 ppm to 2 and 2', and the pair of peaks at -14.3 and -15.6 ppm to 7 and 7'. If we did not know from the X-ray structure that the compound is 1-HS-*syn*-$B_{18}H_{21}$, we could have deduced it from our chemical expectation that the substitution would occur in one of the four positions 1, 2, 3, and 4, and the fact that all the peaks in the areas around those of the borons 3, 4, and 2 of the parent borane are doublets here. We can expect that the singlet at the highest field, that at -3.8 ppm, will belong to the boron 5 (-4.1 ppm in the parent), while choosing between the assignment of 6 (9.7 ppm in the parent) and 1 (1.7 in the parent) to the singlets at 11.0 ppm and 8.3 ppm is trickier, as the ^{11}B COSY shows.

In the ^{11}B-^{11}B COSY (Figure S12), we may not see all the cross peaks we could expect according to what we have seen in the COSY of the parent, but after the analysis of the spectrum we were able to fine tune its transformation so that we can find at least tentative traces of most, keeping still the occurrence of noise and artefacts at an acceptable level (Figure S13). For 5, we expect cross peaks with 1, 1', 2, 2', and 10, 10', for 6 with 2, and 2' only, and for 1 with 2, 3, 4, 5, and 10, and no one with any atom of the unsubstituted subunit. The singlet at -3.8 ppm has a strong cross peak with that at 11.0 ppm, one more strong one at 2.0 ppm, a weaker one with the peak of 2, 2', and a weak one at 8.6 ppm, with one of the peaks coinciding at 8.3 ppm in the 1D spectrum. That is in accord with the assignment of -3.8 ppm to the boron 5, and points to the singlet at 11.0 ppm belonging to the boron 1, not 6. This is confirmed by the singlet at 11.0 ppm having multiple cross peaks besides that with 5: also with 16.7 ppm (so it is 3, and 15.0 ppm will be 3'), -37.2 ppm (4, and -40.6 ppm will be 4'), -28.0 ppm (2, coinciding with 2'), and with one of the peaks coinciding at 8.3 ppm — only 10 remains to be assigned, and this position corresponds to the cross peak of 5 at 8.6 ppm, the exact position of which is possibly affected there by the neighbouring strong cross peak of 5 with 1. There is another cross peak of the coinciding pair of signals at 8.3 ppm with 2, 2' around [8.3 ppm, -28.0 ppm], and even one uncertain weak one with 4 at [8.3 ppm, -37.2 ppm], so as those signals coinciding at 8.3 ppm should be those of the borons 6 and 10, for 6 we expect the only cross peak with 2, 2', and for 10 those with 1, 4, and 5, we should assign the chemical shift of 8.3 ppm to both borons 6 and 10, their coincidence exact within the error of our measurement.

As the boron 3 in the parent borane has cross peaks with 1, 2, 4, 7, and 8, we can, after the assignment of 11.0 ppm to 1, and consequently 16.7 ppm to 3 and -37.2 ppm to 4 follow the cross peaks of 3 (16.7 ppm) and 3' (15.0 ppm), confirm the assignment of -37.2 ppm to 4 and -40.6 ppm to 4', assign -28.0 ppm to 2 and -28.4 ppm to 2', -14.3 ppm to 7 and -16.6 to 7', and 2.0 ppm to 1', and end up assigning the weak cross peaks at 0.0 ppm to 3 with 8, and at -2.0 ppm to 3' with 8'; we can verify this assignment by looking up other weak cross peaks of 8 with 7, 8' with 7', and 8' with 4', that of 8 with 4 possibly present as well, but hidden by the vicinity of a much stronger cross peak. The assignment of 2.0 ppm to 1' is confirmed by its cross peaks with 5, 2', and 4', the last unassigned of them at 9.7 ppm thus belonging necessarily to 10'. Boron 4 in the parent borane interacts with 1, 3, 8, 9, and 10, here we see cross peaks of 4 with 1 and 3, have already discussed the weak one with 10 and apparent absence of that with 8, so the last one, strong enough, at 1.1 ppm remains for 9; analogically we have already assigned the strong cross peaks of 4' with 1' and 3', as well as the weak ones with 10' and 8', so the last unassigned strong cross peak 3.3 ppm must correspond to 9'.

We thus get the observed ^{11}B NMR (192.6 MHz, 295K, $CDCl_3$) signals of 1-HS-*syn*-$B_{18}H_{22}$ assigned in the sequence:: B3 (16.7 ppm) > B3' (15.0 ppm) > B1 (10.9 ppm) > B10' (9.6 ppm) > B10 (8.3 ppm, according to the B10–B5 cross peak 8.6 ppm) >= B6 (8.3 ppm, according to the B6–B2,B2' cross peak 8.3 ppm) >B 9' (3.3 ppm) > B1' (2.0 ppm) > B9 (1.0 ppm) > B8 (0.0 ppm) > B8' (-2.0 ppm) >B 5 (-3.8 ppm) > B7 (-14.3 ppm) > B7' (-16.6 ppm) > B2 (-28.1 ppm, according to the B2–B1 and B2–B3 cross peaks: -28.0 ppm on the x-axis, -27.9 on the y-axis) >= B2' (-28.1 ppm, according to the B2'–B3' and B1'–B2' cross peaks: -28.4 ppm on the x-axis, -28.3 on the y-axis) > B4 (-37.2 ppm) > B4' (-40.6 ppm).

The cross peaks we see in the ^{11}B–^{1}H HMQC NMR spectrum (Figure S14) do not allow us to assign ^{1}H NMR shifts to all the hydrogen atoms in the molecule. Looking up the cross peaks of the boron atoms from 3 (16.7 ppm) to 4' (-40.6 ppm), we find the ^{1}H chemical shifts 4.2 ppm for 3, and 3.9 ppm for 3'. The substituted boron 1 has no terminal hydrogen, but 10' does, and we see only its cross peak with the bridging hydrogen 10'–9' at -1.0 ppm anyway. The case of 10 and 6 is identical, μH(10, 9) has the chemical shift -0.4 ppm, and μH(6, 7) 1.7 ppm; even the expected cross peak of B(6) with μH(6, 7') is absent. The boron 9' has, besides its cross peak with μH(10', 9') that with μH(9', 8') at -2.5 ppm, but again none with a terminal hydrogen, only for 1' we see the cross peak with H(1') at 3.7 ppm. μH(9, 8) has chemical shift -2.5 ppm but only B(8') has also a visible cross peak with its terminal hydrogen at 3.1 ppm. For the boron 5 we do not expect contact with any hydrogen but at the borons 7 and 7' we again have expected cross peaks missing, 7 only having on with μH(6, 7), and 7' in contrast with H(7') at 2.6 ppm. According to the only cross peak of the coinciding signals of B(2) and B(2'), H(2) and H(2') probably have the same chemical shift -0.2 ppm, and the cross peaks of the borons 4 and 4' show the chemical shifts of H(4) as 0.7 ppm and of H(4') as 0.6 ppm. At least the five bridging hydrogens identified of the expected six confirm our assignment of the ^{11}B spectrum.

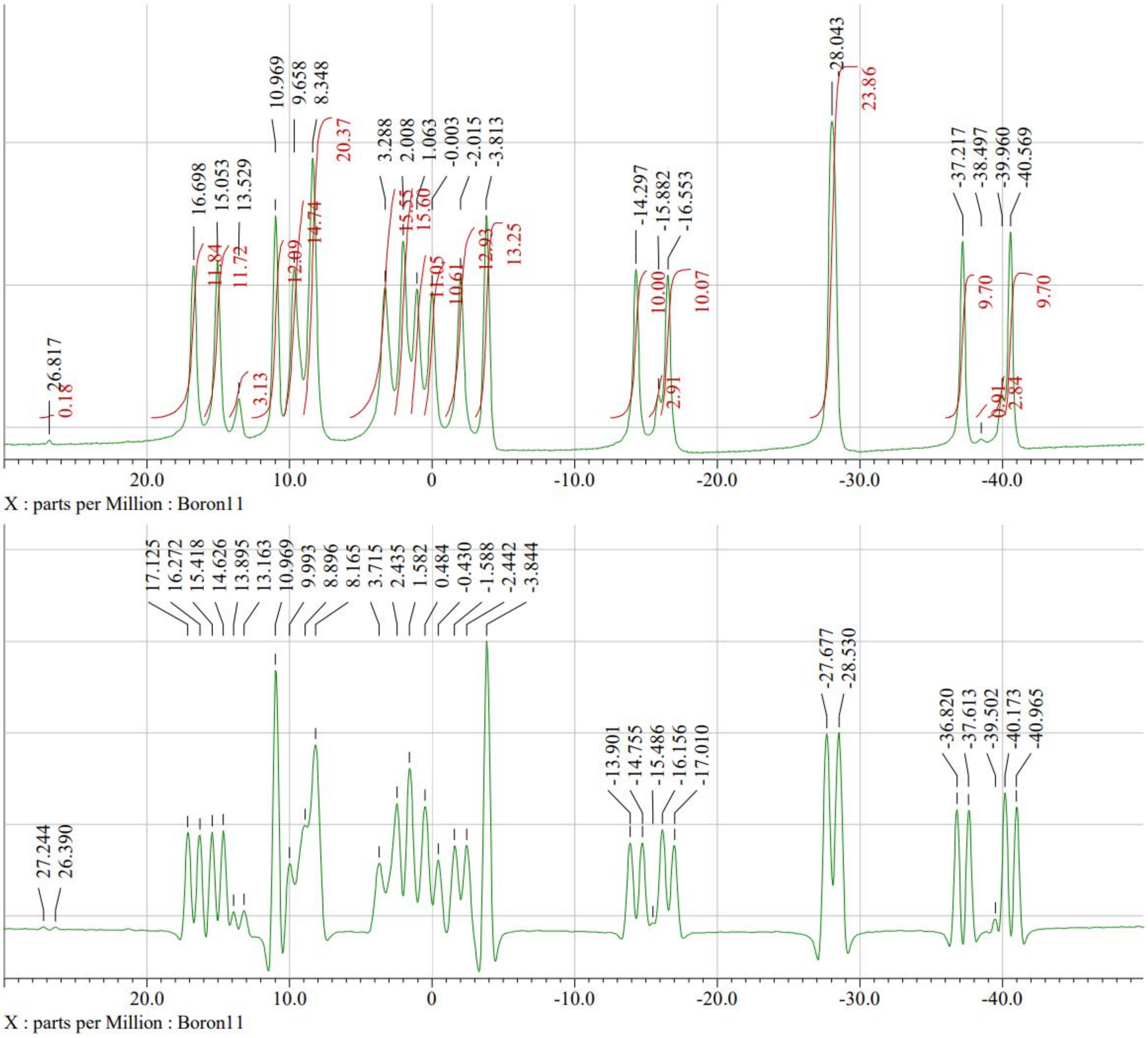

Figure S10. Experimental decoupled (top) and coupled (bottom) ^{11}B NMR spectra of 1-HS-*syn*-$B_{18}H_{21}$ (**1**).

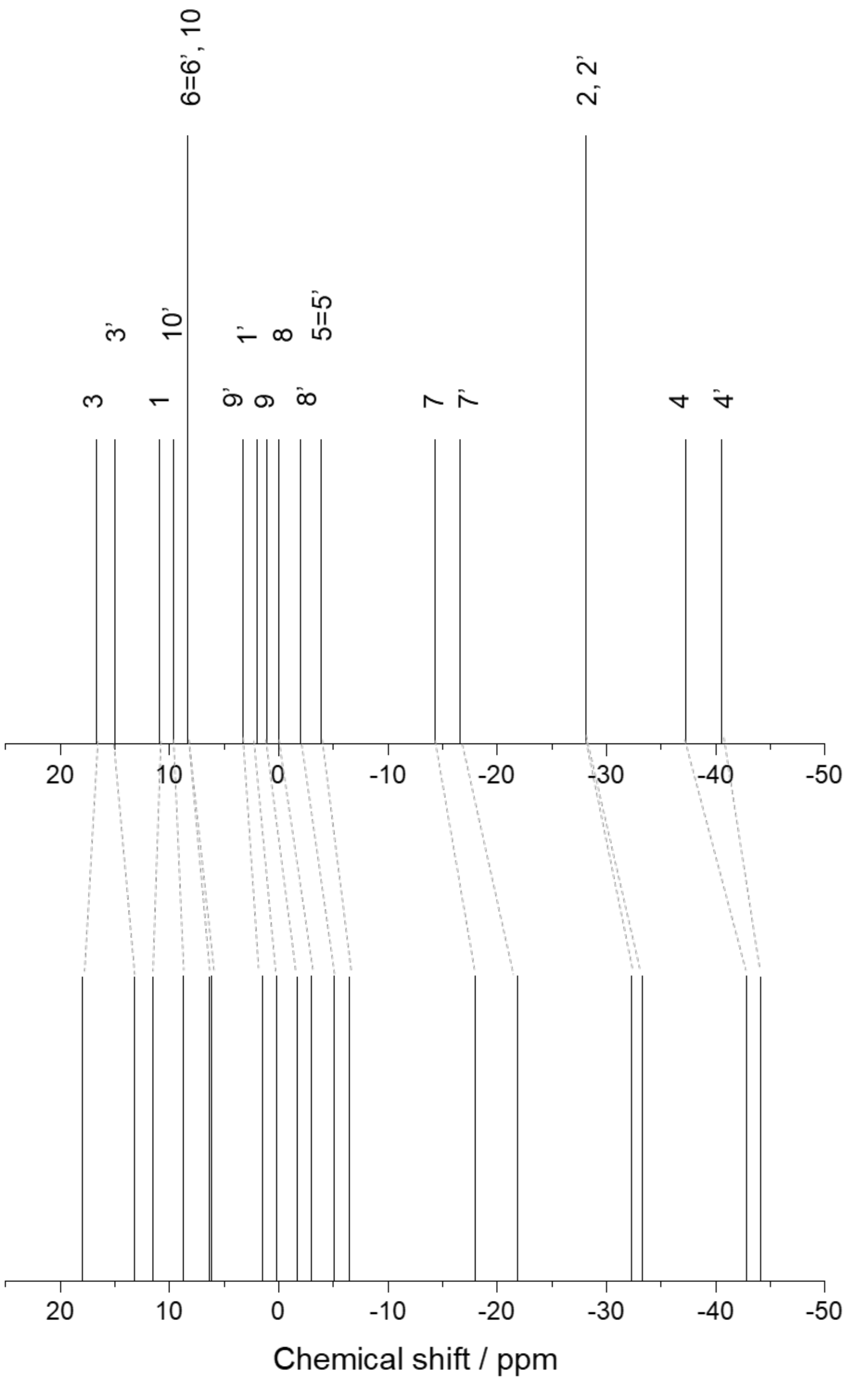

Figure S11. Correlation between the (top) experimental and (bottom) computational ^{11}B NMR spectra of 1-HS-*syn*-$B_{18}H_{21}$ (**1**). Ratio of the boron signal intensity of experimental ^{11}B NMR: 1:1:1:1:2:1:1:1:1:1:1:1:1:2:1:1

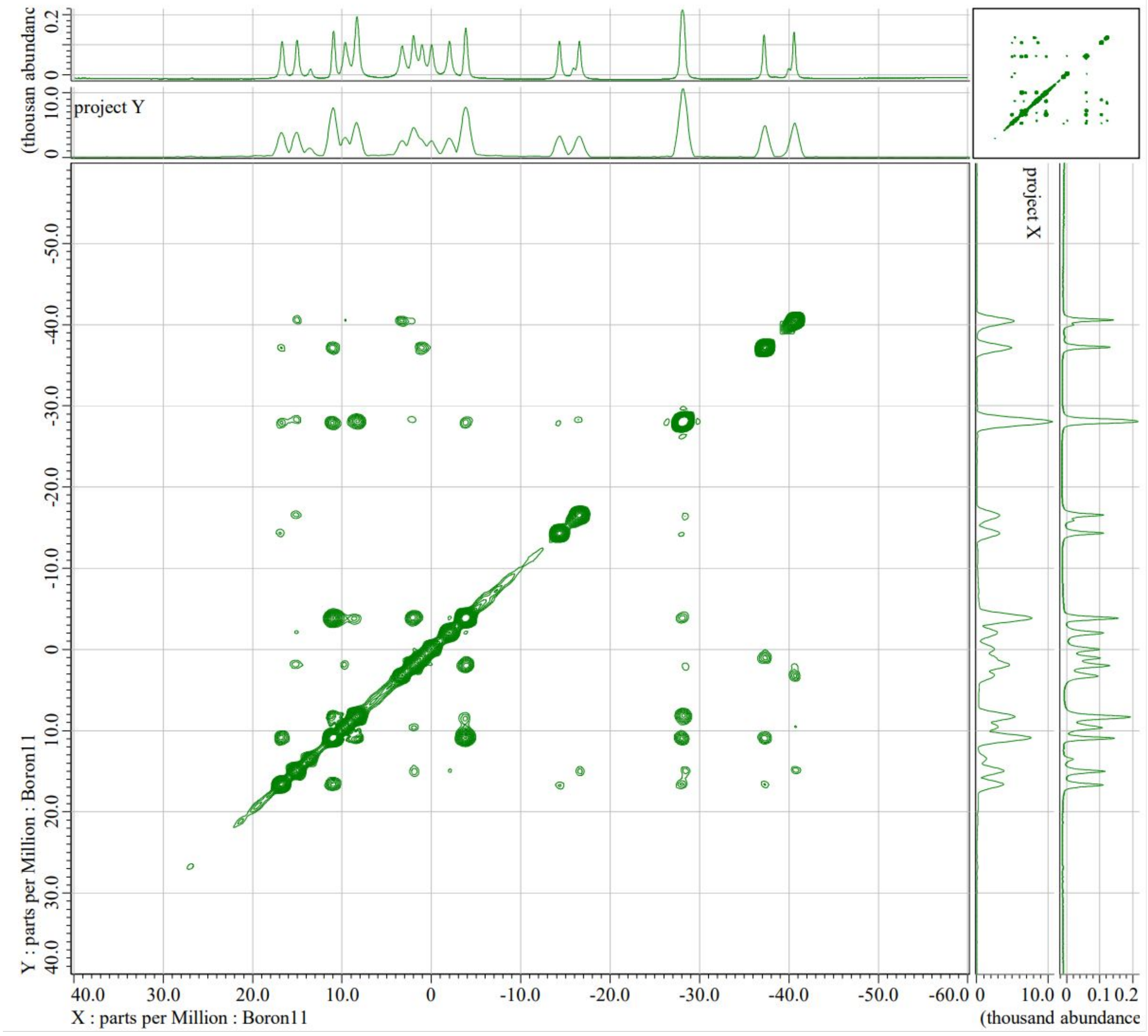

Figure S12. 2-D [^{11}B-^{11}B]-COSY-{^{1}H} NMR spectrum of 1-HS-*syn*-$B_{18}H_{21}$ (**1**).

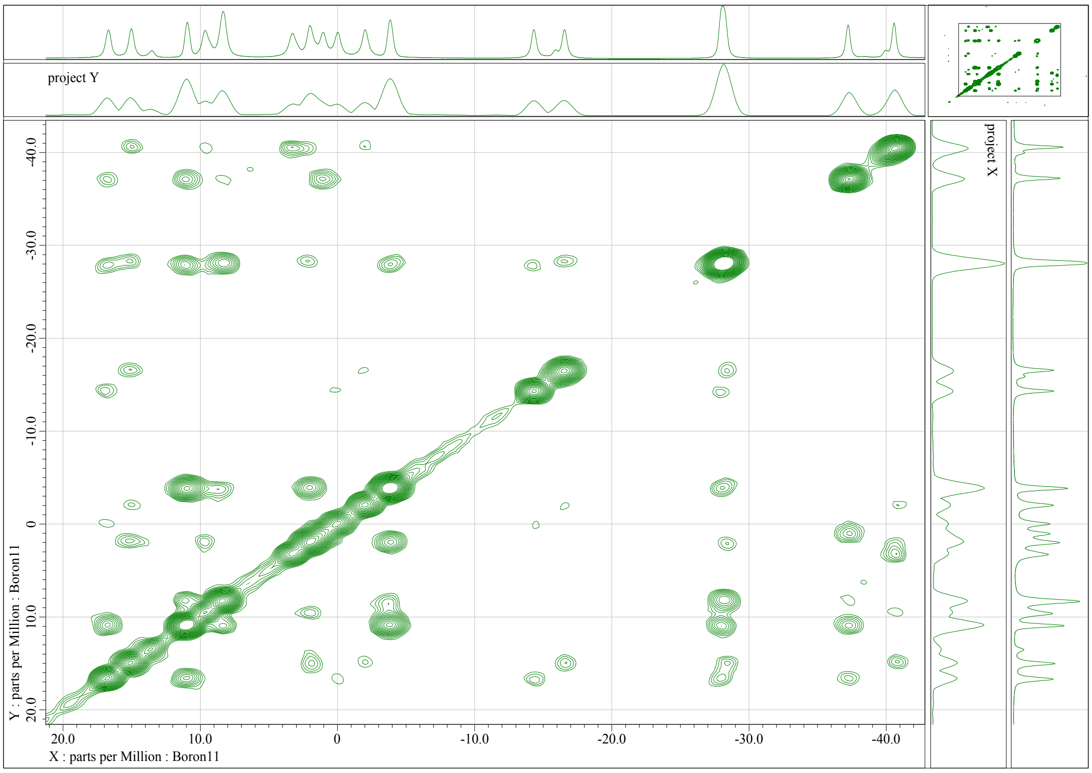

Figure S13: ^{11}B COSY NMR spectrum of 1-HS-*syn*-$B_{18}H_{21}$ treated to see as many cross peaks as possible.

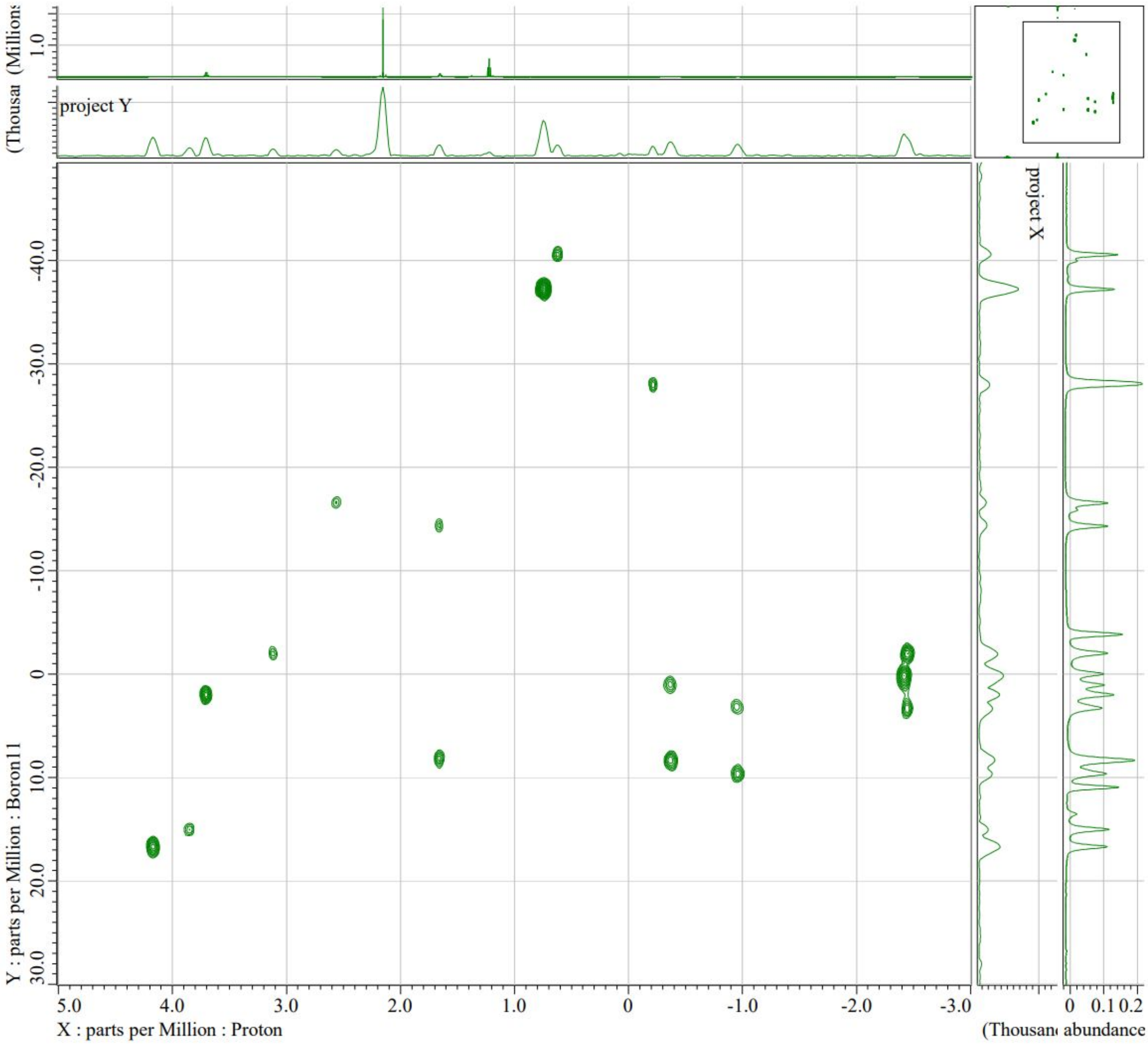

Figure S14. 2-D [^{1}H-^{11}B]-HMQC NMR spectrum of 1-HS-*syn*-$B_{18}H_{21}$ (**1**).

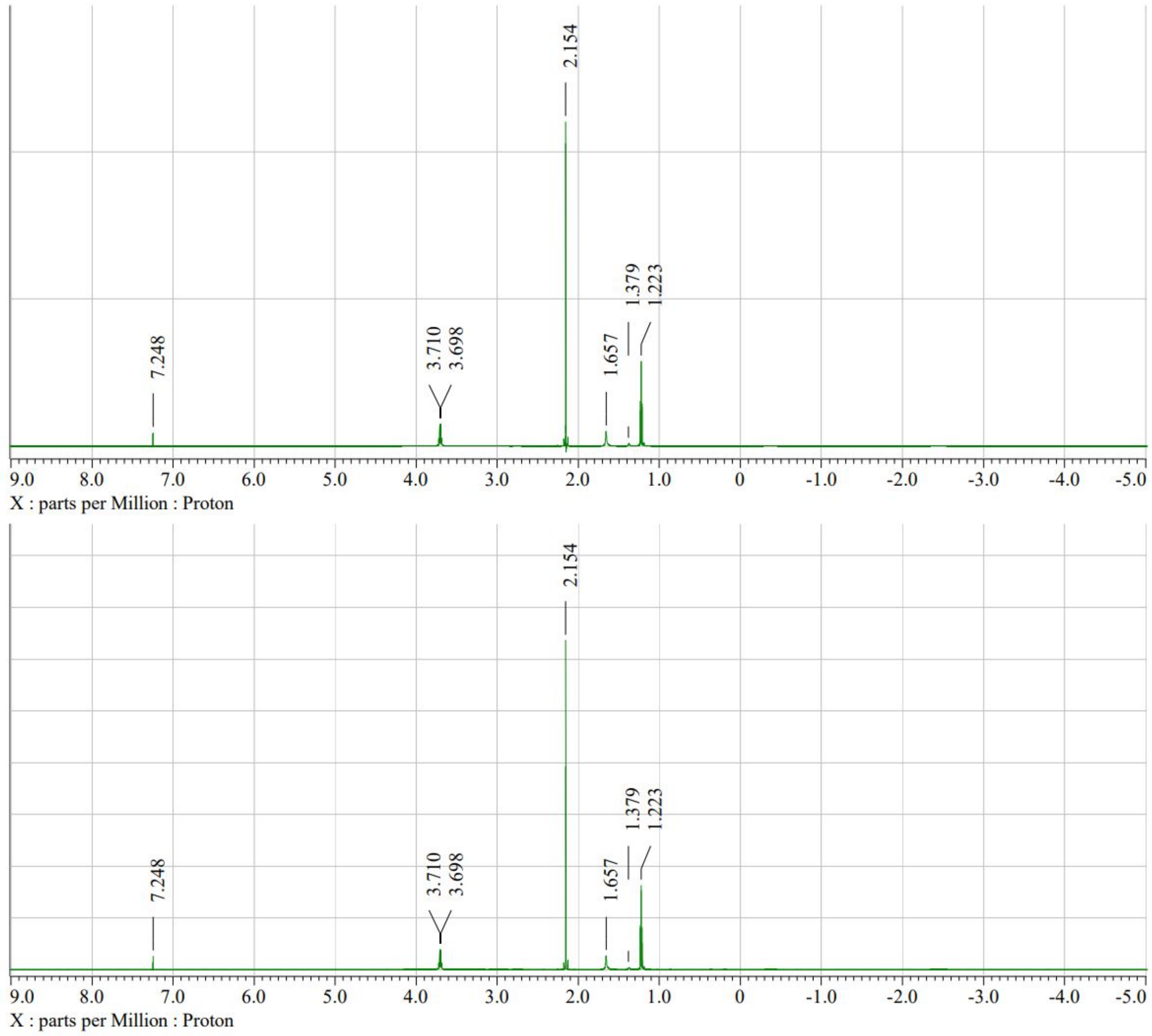

Figure S15. $^{1}H\{B\}$ decoupled (top) and coupled (bottom) proton NMR spectra of 1-HS-*syn*-$B_{18}H_{21}$ (**1**)

3-HS-*syn*-$B_{18}H_{21}$ ^{11}B NMR assignment

In the comparison of ^{1}H decoupled and simple (coupled) ^{11}B NMR spectra of 3-HS-*syn*-$B_{18}H_{21}$ (Figure S16) we see immediately three singlets, that we can on the basis of the parent borane spectrum assign to the substituted position 3 (21.4 ppm) and the two borons common to both subunits, that have no terminal hydrogens, 6 (6.6 ppm) and 5 (-2.2 ppm). The decoupled spectrum contains 17 peaks, in one of them the singlet of B(5) coincides with a doublet of another boron atoms, and all the signals of the other 16 boron atoms are individually resolved. Still by the analogy with the spectra of the parent we can assign 15.0 ppm to 3' and tell that the three pairs in the upfield part of the spectrum belong -15.4 ppm and -16.8 ppm to 7 and 7', -28.6 ppm and -29.4 ppm to 2 and 2', and -38.5 ppm and -39.4 ppm to 4 and 4'.

Again, we verify and complete the assignment by interpreting the ^{11}B–^{11}B COSY NMR spectrum (Figure S18). Cross peaks of 3 and 3' allow us to assign -38.5 ppm to 4 and -39.4 ppm to 4', -28.6 ppm to 2 and -29.4 ppm to 2', -29.4 ppm to 7 and -28.6 ppm to 7', and 4.4 ppm to 1 and 2.7 ppm to 1', and -3.2 ppm to 8, and -2.0 ppm to 8', in the 1D spectrum coinciding with 5 at -2.2 ppm. As we expect for 5 cross peaks with 1, 1', 2, 2', 10, and 10', for 8'

with 3', 4', and 7', and for 1' with 2', 3', 4', 5, and 10', our assignment is verified by the cross peaks at [-2.2 ppm, 4.4 ppm] and [-2.2 ppm, 2.6 ppm] that can only belong to 5–1 and 5–1' because 8 and 8' cannot have cross peaks with 5, nor with 1'. We do not see the cross peaks of 5 with 10 and 10' but 1 has a weak cross peak with 10 at 10.3 ppm, and 1' has similar with 10' at 9.7 ppm. Even though we do not see all the expected cross peaks for 4 and 4', there are strong enough cross peaks of 4 with 9 at 0.8 ppm and 4' with 9' at 9.7 ppm. The assignment of the ^{11}B spectrum is thus complete, and other visible cross peaks like those of 5 with 2 and 2' of 6 with 2 and 2' or those of 1 with 2 and 1' with 2' corroborate it.

The sequence of the observed ^{11}B NMR signals of 3-HS-*syn*-$B_{18}H_{22}$ is: 3 (21.4 ppm) > 3' (13.5 ppm) > 10 (10.3 ppm) > 10' (9.7 ppm) > 6 (6.6 ppm) > 1 (4.4 ppm) > 9' (3.7 ppm) > 1' (2.7 ppm) > 9 (0.8 ppm) > 8' (-2.0 ppm) >= 5 (-2.2 ppm) > 8 (-3.2 ppm) > 7' (-15.4 ppm) > 7 (-16.8 ppm) > 2 (-28.6 ppm) > 2' (-29.4 ppm) > 4 (-38.5 ppm) > 4' (-39.4 ppm).

An interesting feature in the ^{11}B–^{1}H HMQC spectrum of 3-HS-*syn*-$B_{18}H_{21}$ (Figure S19) is the cross peak of the substituted B(3) at 1.4 ppm — with the thiol group hydrogen, in the ^{1}H NMR spectrum decoupled by ^{11}B represented by a very sharp peak, and without decoupling only slightly broadened (Figure S20). 3' has a cross peak with its terminal hydrogen at 3.8 ppm. For 10 and 10' we have detected cross peaks only with their bridging hydrogens with B(9) and B(9'); the chemical shifts of those hydrogens are -0.9 ppm, identical within our experimental error, even though μH(9, 10) is visibly upfield of μH(9', 10'). B(6) has strong cross peaks with its bridging hydrogens, μH(6, 7) at 1.9 ppm, downfield from μH(6, 7') at 1.1 ppm. According to the cross peaks of 1 and 1', H(1) has chemical shift 4.0 ppm, and H(1') 3.6 ppm. For borons 9 and 9' we see only their cross peaks with their bridging hydrogens, besides μH(9, 10) and μH(9', 10'), there are μH(8, 9) at -2.1 ppm and μH(8', 9') at -2.5 ppm. As B(5) is not connected to any hydrogen atom, it does not interfere with the assignment of the chemical shift 3.5 ppm to H(8´), while H(8) has 3.1 ppm, according to a weak cross peak. The borons 7 and 7' have, besides their cross peaks with μH(6, 7) and μH(6, 7') those with their terminal hydrogens too, even though that of B(7) with H(7) at 2.6 ppm is weaker than that of B(7') with H(7') at 2.7 ppm, just like that of B(8) with H(8) in comparison to that of B(8') with H(8'). According to their cross peaks with B(4) and B(4'), H(4) has chemical shift 0.7 ppm and H(4') 0.6 ppm. For B(2) and B(2'), we had to adjust the parameters of the transformation of the spectrum to resolve their cross peaks with H(2) and H(2') at the expense of others (Figure S21): follows that H(2) is slightly upfield of H(2') but within our experimental error their chemical shifts are identical, -0.2 ppm.

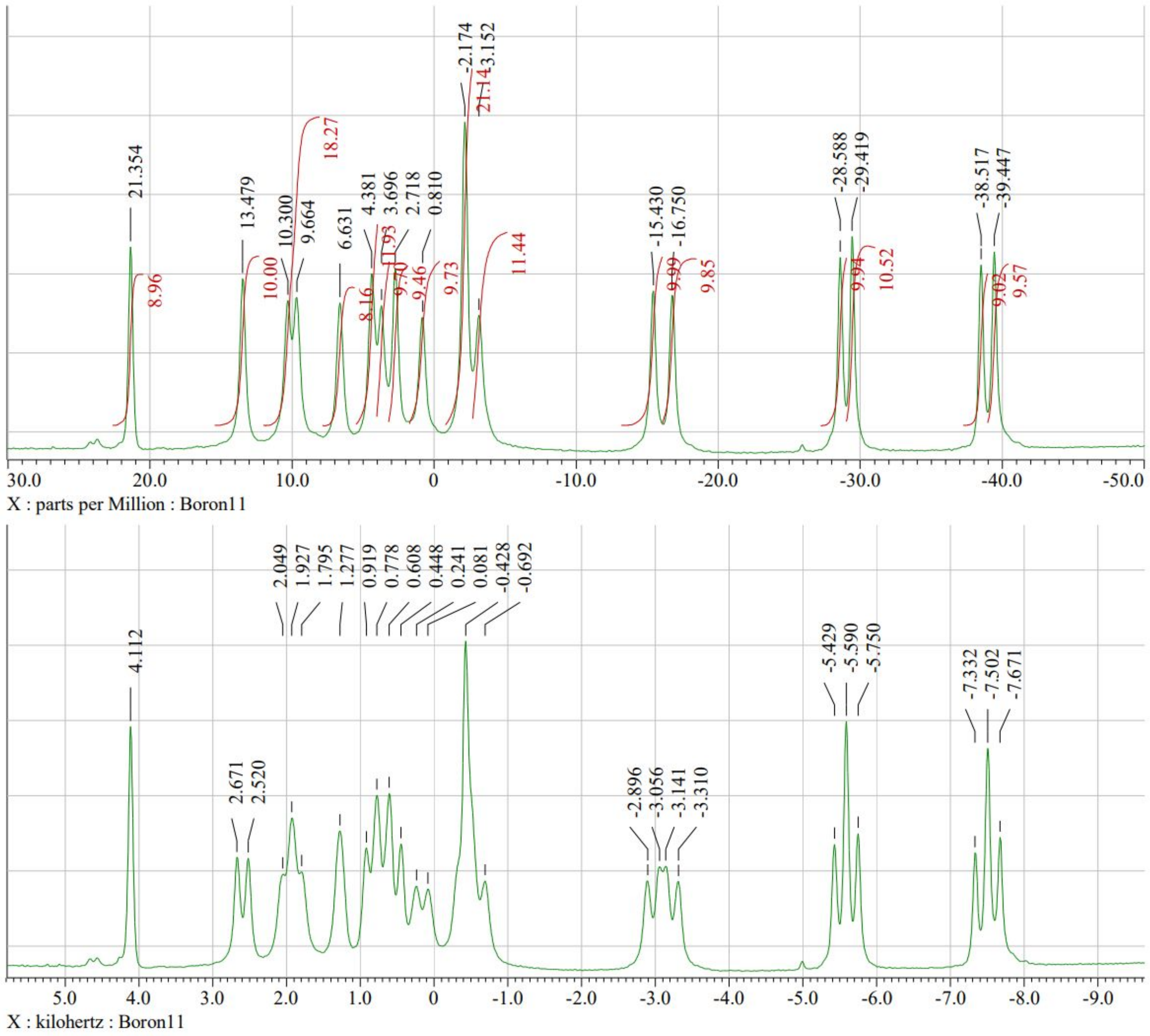

Figure S16. Experimental decoupled (top) and coupled (bottom) ^{11}B NMR spectra of 3-HS-*syn*-$B_{18}H_{21}$ (**3**).

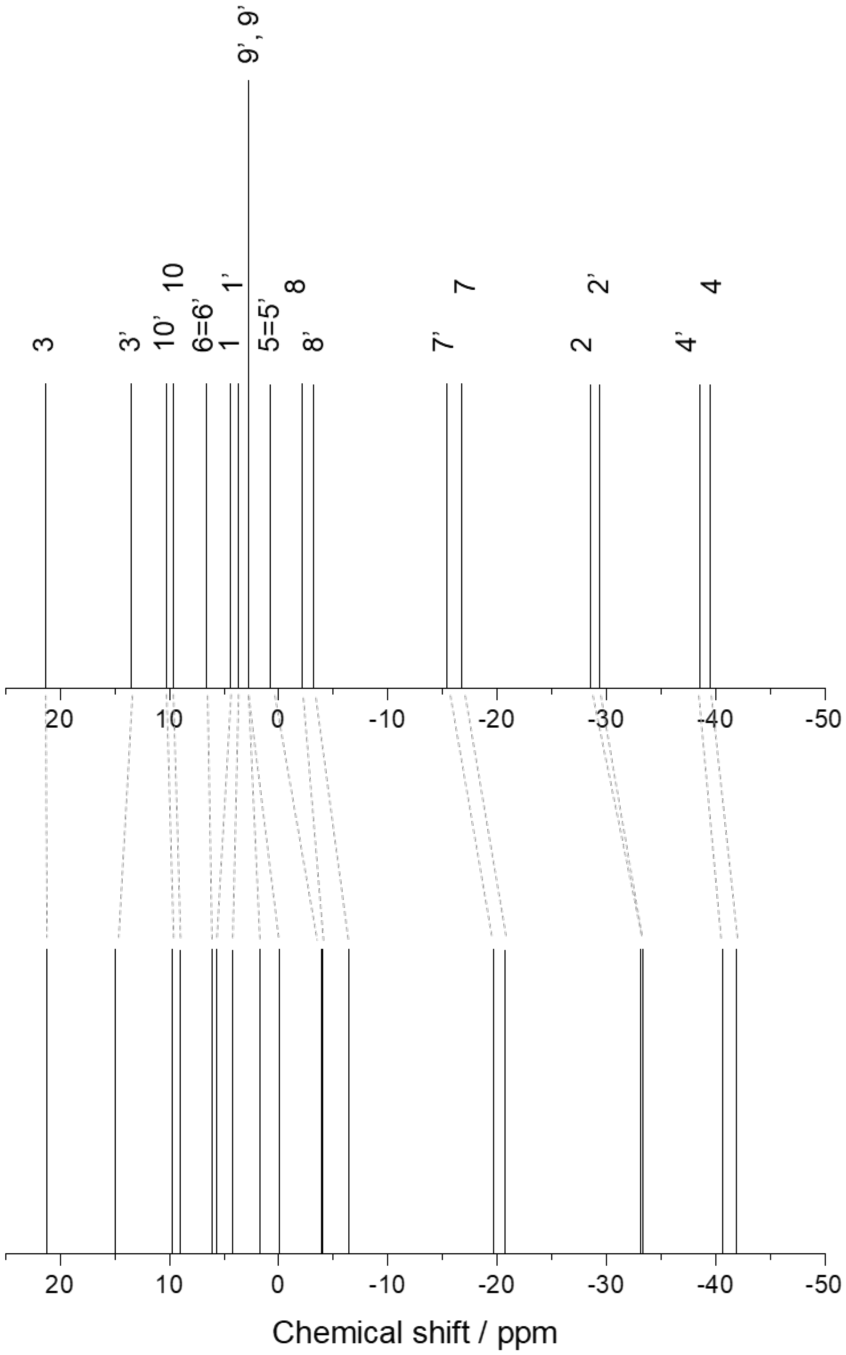

Figure S17. Correlation between the (top) experimental and (bottom) computational ^{11}B NMR spectra of 3-HS-*syn*-$B_{18}H_{21}$ (**3**). Ratio of the boron signal intensity of experimental ^{11}B NMR: 1:1:1:1:1:1:1:2:1:1:1:1:1:1:1:1:1.

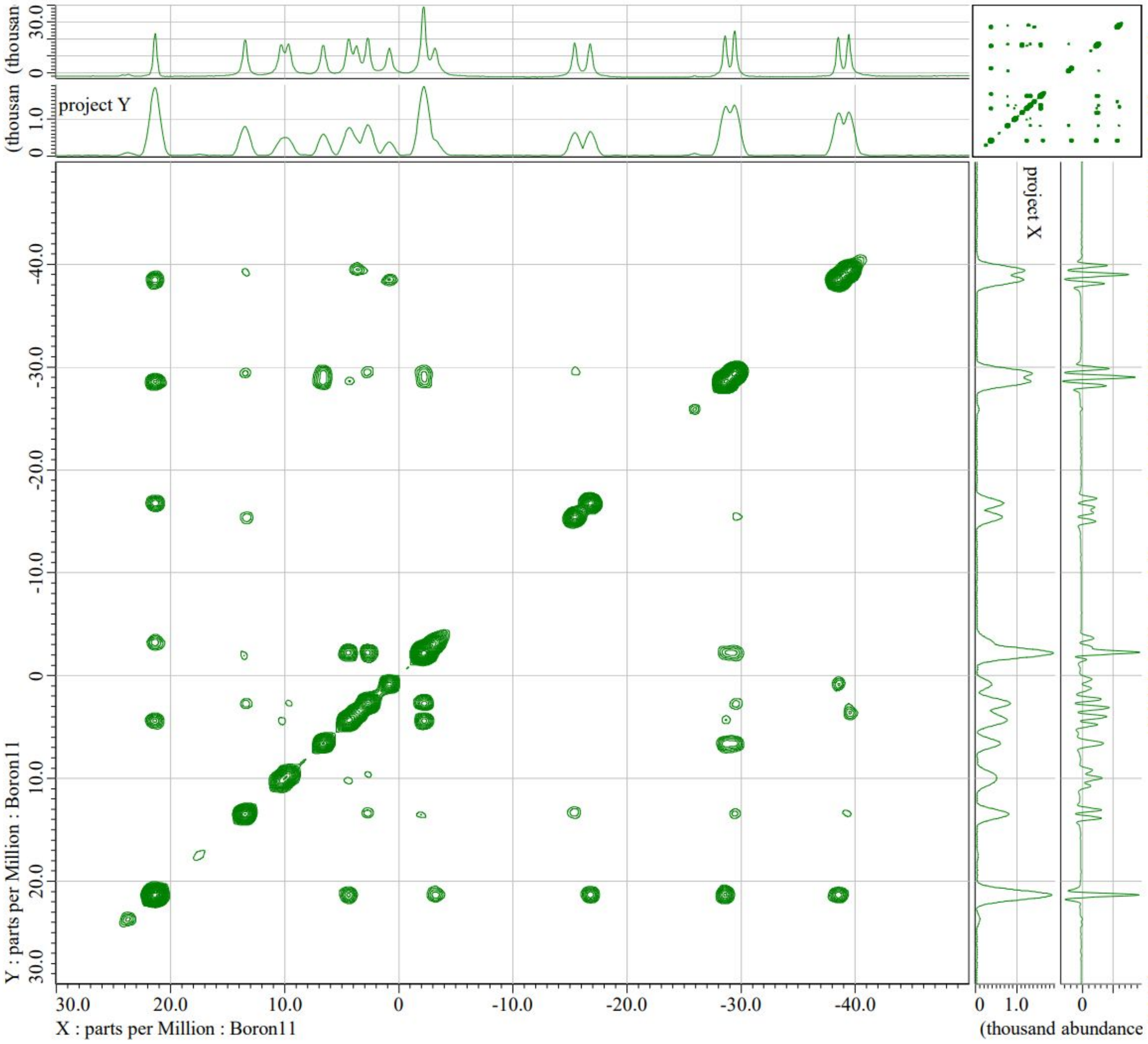

Figure S18. 2-D [^{11}B-^{11}B]-COSY-{^{1}H} NMR spectrum of 3-HS-*syn*-$B_{18}H_{21}$ (**3**).

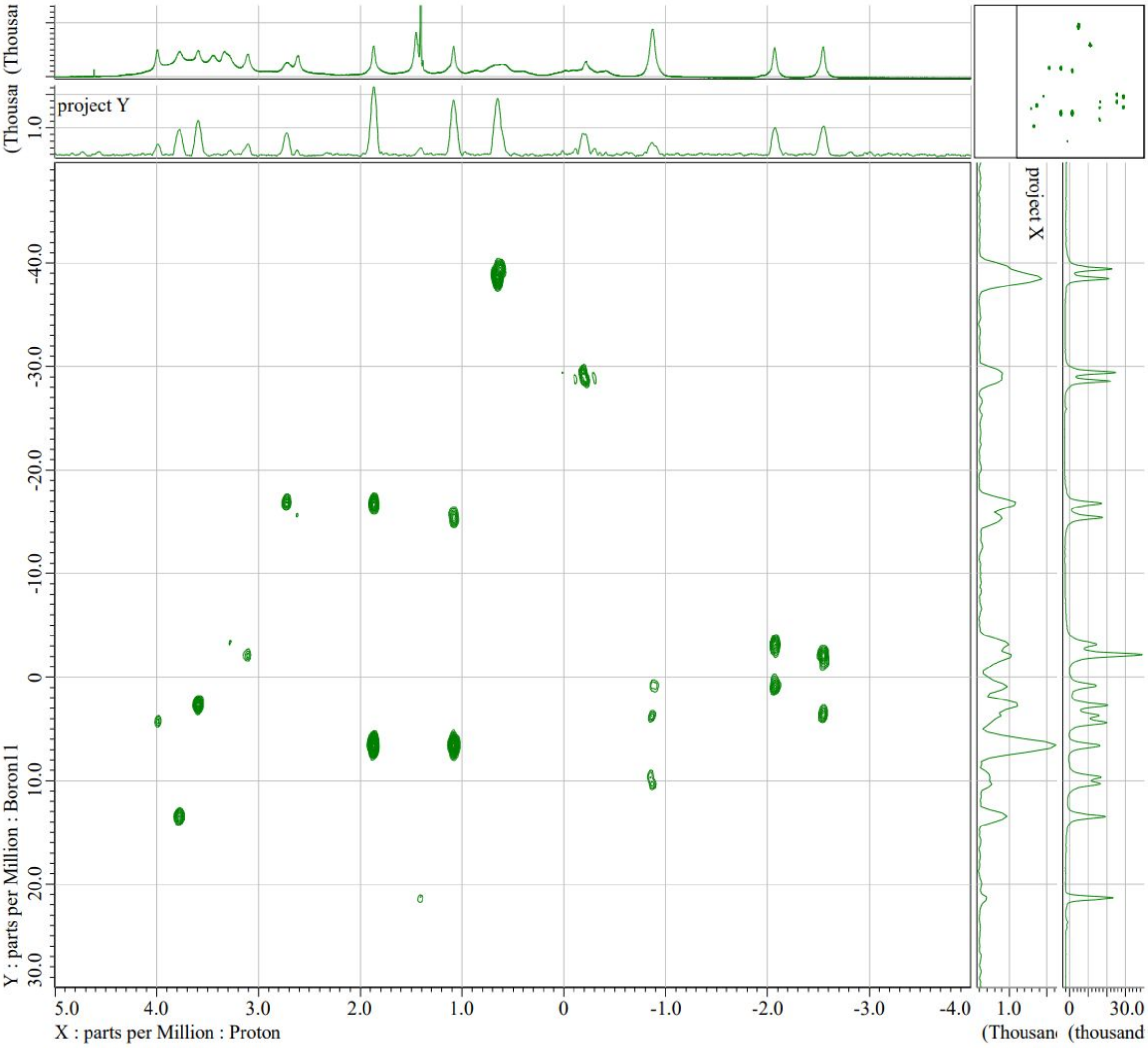

Figure S19. 2-D [^{1}H-^{11}B]-HMQC NMR spectrum of 3-HS-*syn*-$B_{18}H_{21}$ (**3**).

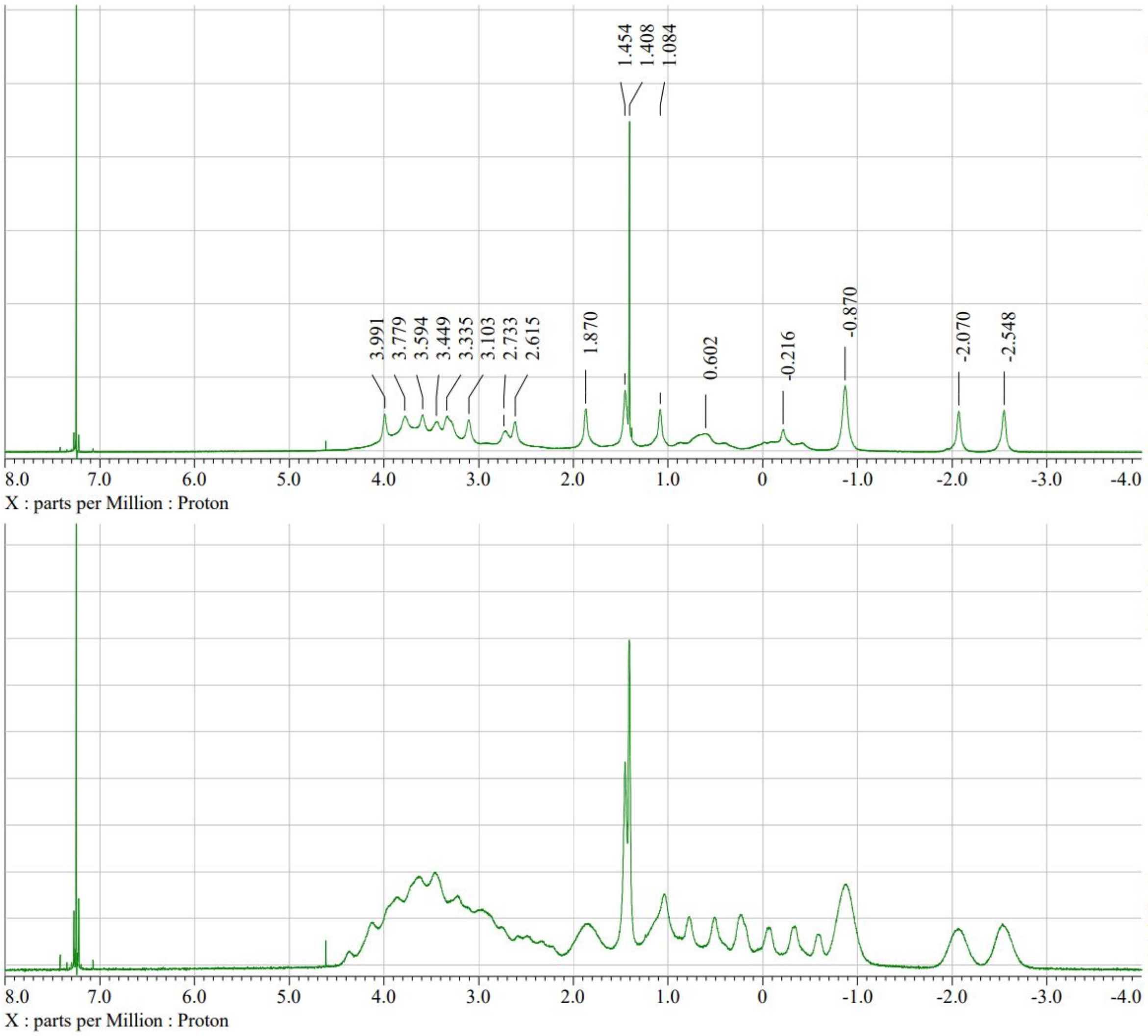

Figure S20. $^{1}H\{B\}$ decoupled (top) and coupled (bottom) proton NMR spectra of 3-HS-*syn*-$B_{18}H_{21}$ (**3**).

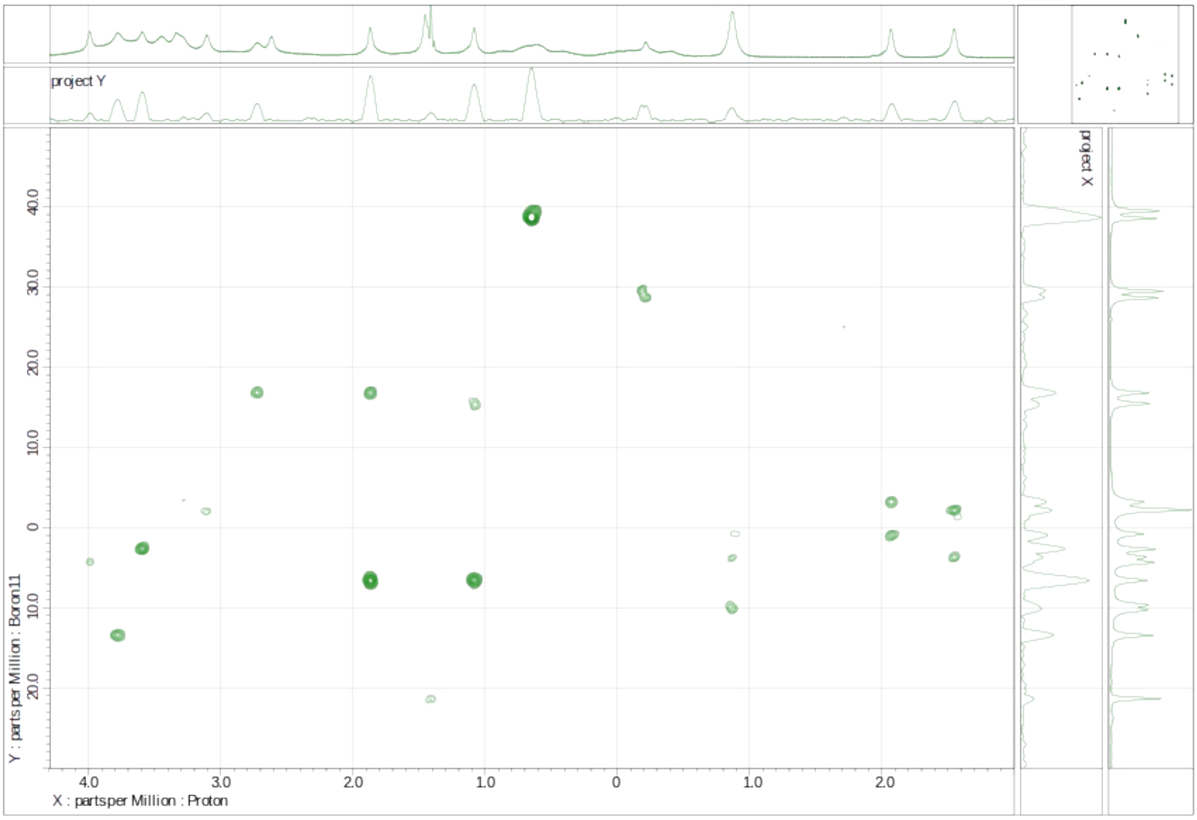

Figure S21: ^{1}H–^{11}B HMQC NMR spectrum of 3-HS-syn-$B_{18}H_{21}$ treated to resolve the cross peaks of B(2) with H(2) and B(2') with H(2')

4-HS-*syn*-$B_{18}H_{21}$ ^{11}B NMR assignment

4-HS-*syn*-$B_{18}H_{21}$ has many ^{11}B NMR signals (Figure S22) very close together, especially the group 1, 1', 9, 9'; 8 and 8' almost coincide, and 7, 7' do not differ at all. Nevertheless, a strong line narrowing transformation (Gaussian window on the FID before the Fourier transform 42 Hz wide and shifted 13 ms for the ^{1}H decoupled spectrum and 50 Hz shifted 10 ms for the coupled one) allows to differentiate all the peaks (Figure S23). Interestingly, there is at -25.8 ppm, in the typical area of 2, 2' signals, a singlet, so likely a signal of a substituted B(2), which suggests that even the 2-HS isomer that we have not obtained in pure form may have been form at least as a very minor product, and present as an impurity.

Singlets at 8.1 ppm, -3.4 ppm, and -29.2 ppm can be assigned to B(6), B(5), and B(4) respectively just on the comparison with the spectra of the parent and the other isomers: 8.1 ppm is upfield of B(6) of the parent, slightly more than in 1-HS and less than in 3-HS, -3.4 ppm is downfield of B(5) in the parent, again more than in 1-HS but less than in 3-HS, and -29.2 ppm is a singlet downfield of B(4) peaks at the upfield end of *syn*-$B_{18}H_{22}$ derivatives spectra, shifted from the parent towards the B(2) region, but must belong to the substituted B(4), even if we did not know from the X-ray that this is the 4-HS isomer, because there are two doublets close to one another at -27.7 ppm and -28.1 ppm, evidently B(2) and B(2') (we only have to find out which is which), and only one doublet of B(4') is left at -39.8 ppm, downfield of 4, 4' of the parent but less than B(4') of 3-HS.

In ^{11}B-^{11}B COSY, B(4) of *syn*-$B_{18}H_{22}$ (Figure S5) has cross peaks with 1, 2, 3, 8, 10, and 9. In the COSY of 4-HS-*syn*-$B_{18}H_{21}$ (Figure S25), no 4'–2' or 4–2 cross peak is seen. There are four cross peaks of 4 (or 5 if there are two at 3.0 and 2.6 ppm fused together), and four of 4', so besides those with 2 and 2', one more must be missing, at least for 4', perhaps that with 8', based on our experience with 1-HS and 3-HS. Assignment of 14.9 ppm to 3 and 13.8

ppm to 3' is easy, both downfield of the parent, 3 about as much as 3' in 1-HS, 3' less. Similarly, 10.3 ppm will be B(10), downfield of the parent about as much as in 3-HS, and 9.1 ppm B(10'), slightly upfield of the parent, but less then B(10) in 1-HS. 4' has a strong cross peak with 3.5 ppm and weaker with 2.0 ppm, 4 has a strong one with 2.6 ppm, and possibly a weaker one with 3.0 ppm, fused with the stronger one. We can assign them, along with 2 and 2', based on the cross peaks of 2 and 2', as this is the area of borons 1 and 9, and in the parent borane, 9 has a cross peak only with 4 (and can hardly have one with 2, not even hypothetically), while 1 has cross peaks with both 4 and 2 (and 3, 5, and 10). The signal at -28.1 ppm has a cross peak with 2.0 ppm, which has a cross peak with 4', so 2.0 ppm is 1', and -28.1 ppm is 2'. It leaves 2 to assign to -27.7 ppm, and its cross peak with 3.0 ppm, that may, with some uncertainty, have one with 4 as well, make 3.0 ppm B(1). Subsequently 3.5 ppm must belong to B(9'), and 2.6 ppm to B(9). The remaining cross peak of B(4) at -1.2 ppm allows us to assign -1.2 ppm to B(8), and consequently -1.6 ppm, even without cross peaks detected, to B(8'). The cross peaks of 1 with 3 and 10, and of 1' with 3' and 10', corroborate this assignment, as do the cross peaks of 3 with 2 and 3' with 2', borons 5 and 6 having cross peaks with both 2 and 2'. The peak at -16.1 ppm that belongs to borons 7 and 7', has cross peaks only with 3 and 3'; those allow to differentiate a bit, albeit around the level of experimental error, between B(7) and B(7'), and to assign -16.1 ppm to 7', and -16.2 ppm to 7. We thus have the signals in the ^{11}B (^{1}H decoupled) NMR spectrum of 4-HS-*syn*-$B_{18}H_{21}$ assigned as: 3 (14.9 ppm) > 3' (13.8 ppm) > 10 (10.3 ppm) > 10' (9.1 ppm) > 6 (8.1 ppm) > 9' (3.5 ppm) > 1 (3.0 ppm) > 9 (2.6 ppm) > 1' (2.0 ppm) > 8 (-1.2 ppm) > 8' (-1.6 ppm) > 5 (-3.4 ppm) > 7' (-16.1 ppm) >= 7 (-16.2 ppm) > 2 (-27.7 ppm) > 2' (-28.1 ppm) > 4 (-29.2 ppm) > 4' (-39.8 ppm).

From the cross peaks in the ^{1}H–^{11}B HMQC spectrum (Figure S26) we see that H(3) has chemical shift 4.0 ppm, H(3') 3.8 ppm, μH(9, 10) -0.3 ppm, μH(9', 10') -0.9 ppm, μH(6, 7') 1.2 ppm, μH(6, 7) 1.4 ppm, μH(8', 9') -2.5 ppm, μH(8, 9) -1.9 ppm, the cross peaks of the terminal hydrogens at the positions 10 and 10' are not detected, H(1) has chemical shift 3.8 ppm, H(1') 3.7 ppm, H(8) 3.4 ppm, H(8') 3.1 ppm, H(7') 2.5 ppm, H(7) 2.7 ppm, H(2) -0.1 ppm, H(2') -0.3 ppm, and H(4') 0.6 ppm. The cross peaks of the bridging hydrogens confirm our assignment of the 11B spectrum.

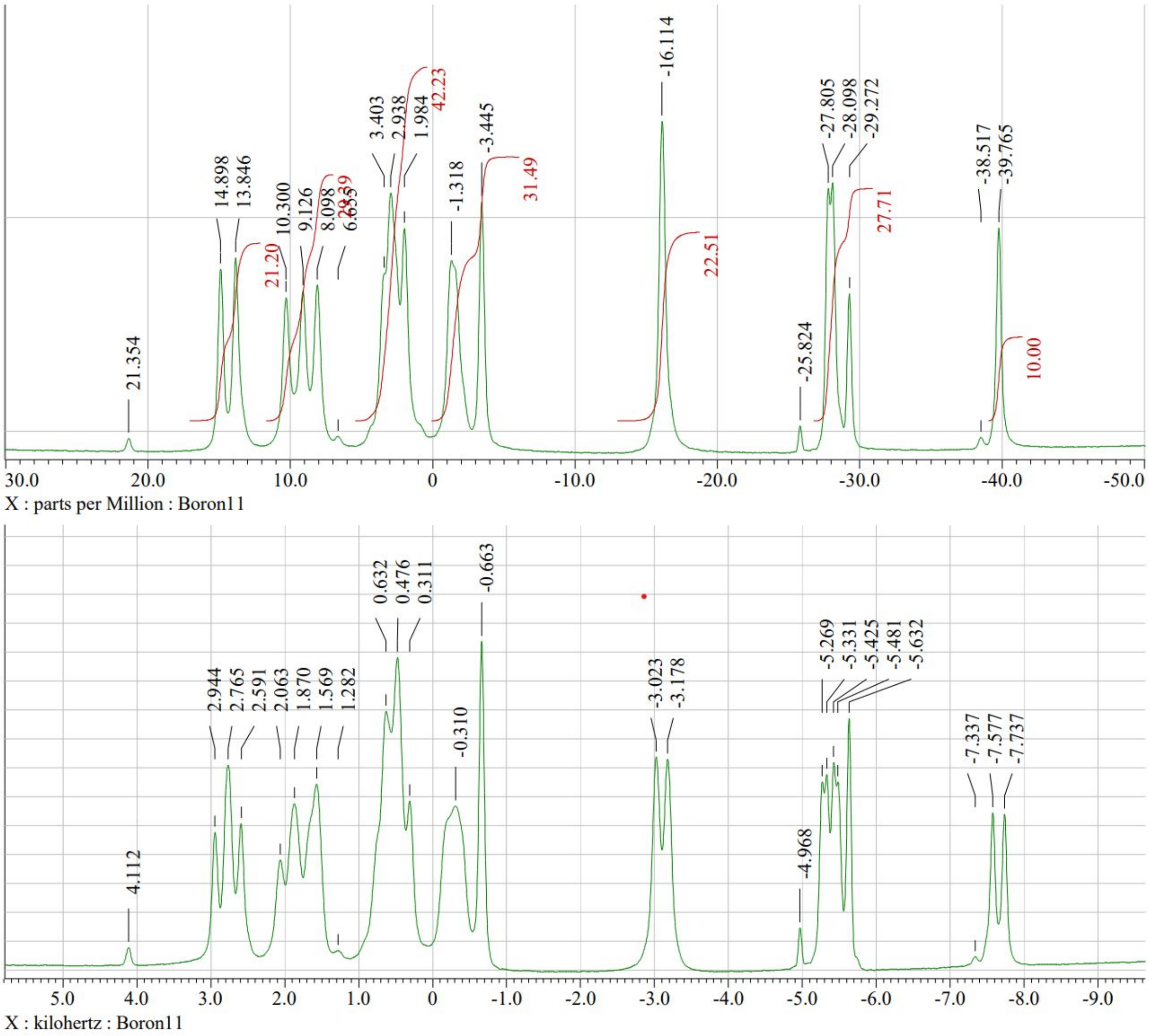

Figure S22. Experimental decoupled (top) and coupled (bottom) ^{11}B NMR spectra of 4-HS-*syn*-$B_{18}H_{21}$.

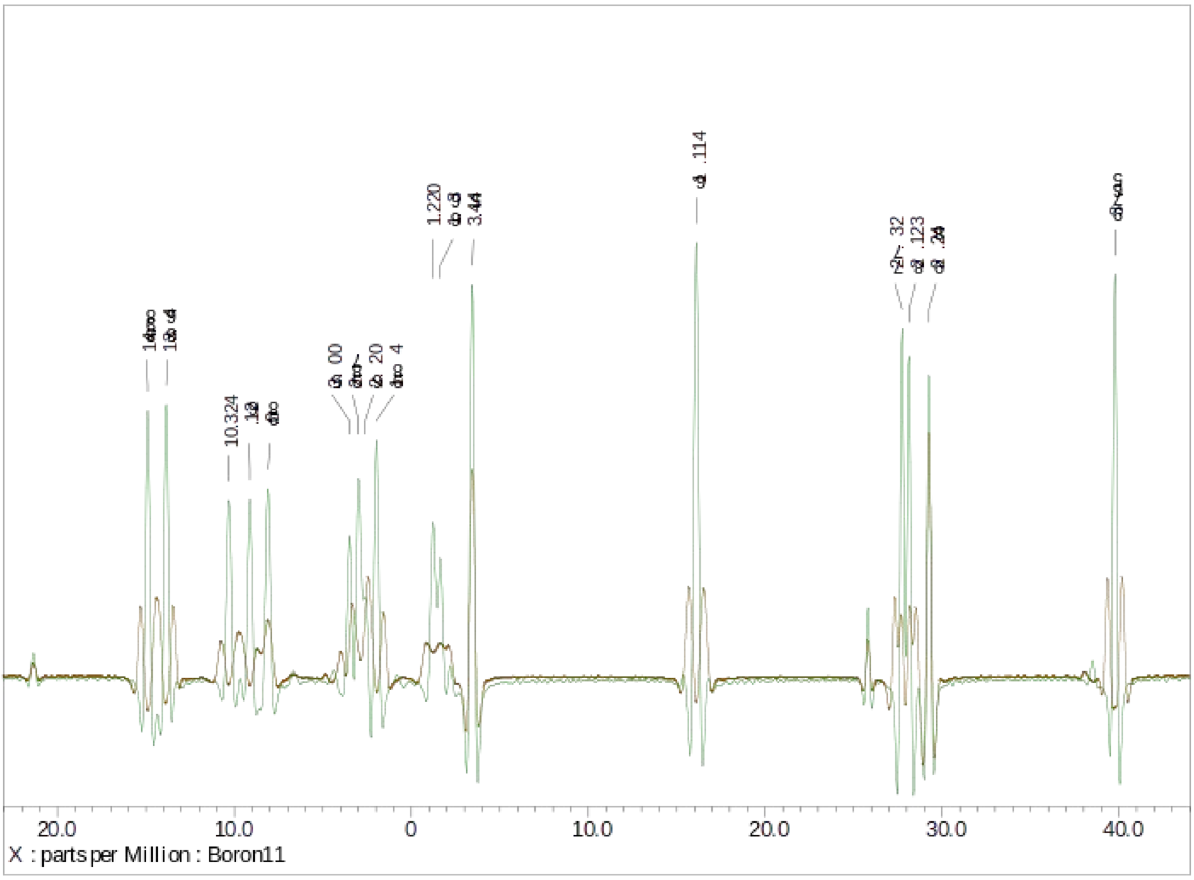

Figure S23: ^{1}H decoupled ^{11}B NMR spectrum of 4-HS-*syn*-$B_{18}H_{21}$ superimposed on the non-decoupled one, transformation of the FID fine-tuned to get lines narrow enough for discriminating all the signals.

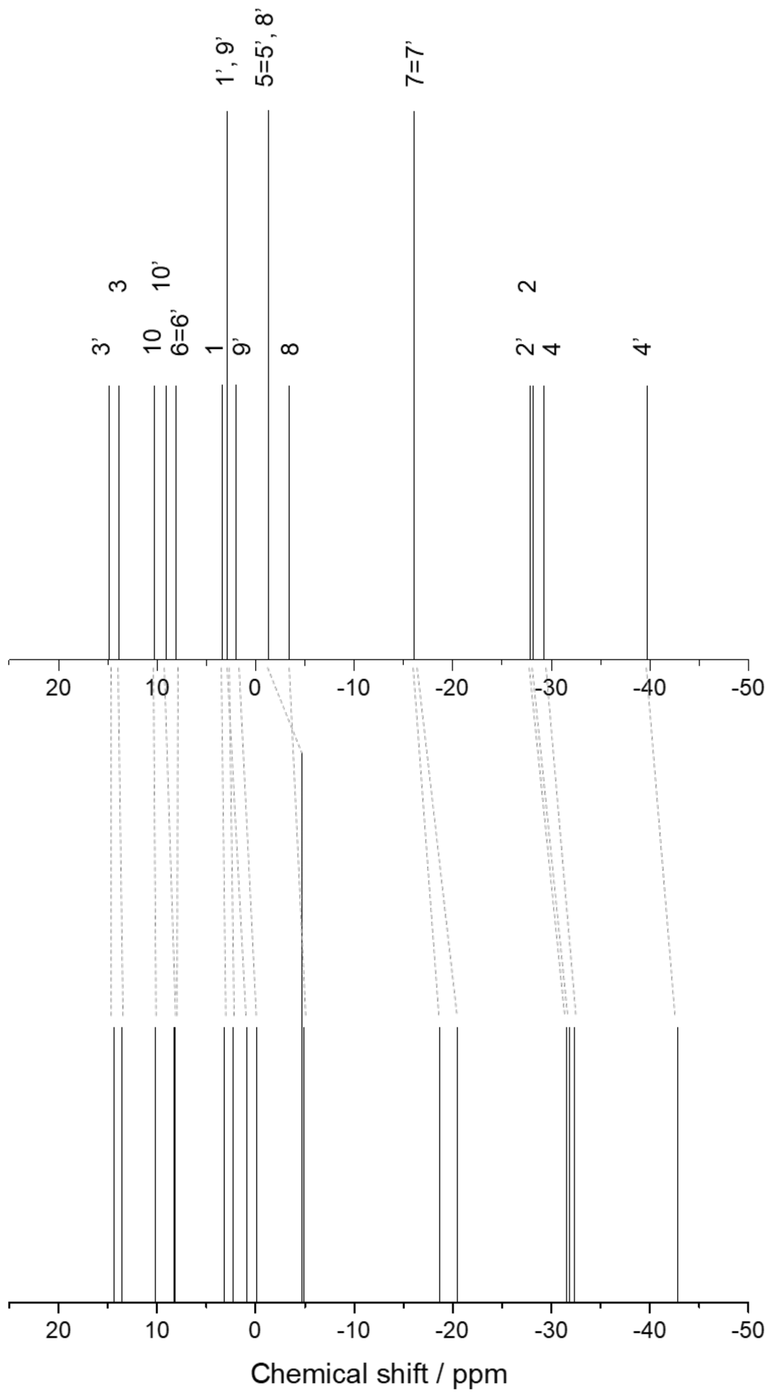

Figure S24. Correlation between the (top) experimental and (bottom) computational ^{11}B NMR spectra of 4-HS-*syn*-$B_{18}H_{21}$ (**4**). Ratio of the boron signal intensity of experimental ^{11}B NMR: 1:1:1:1:1:1:2:1:2:1:2:1:1:1:1

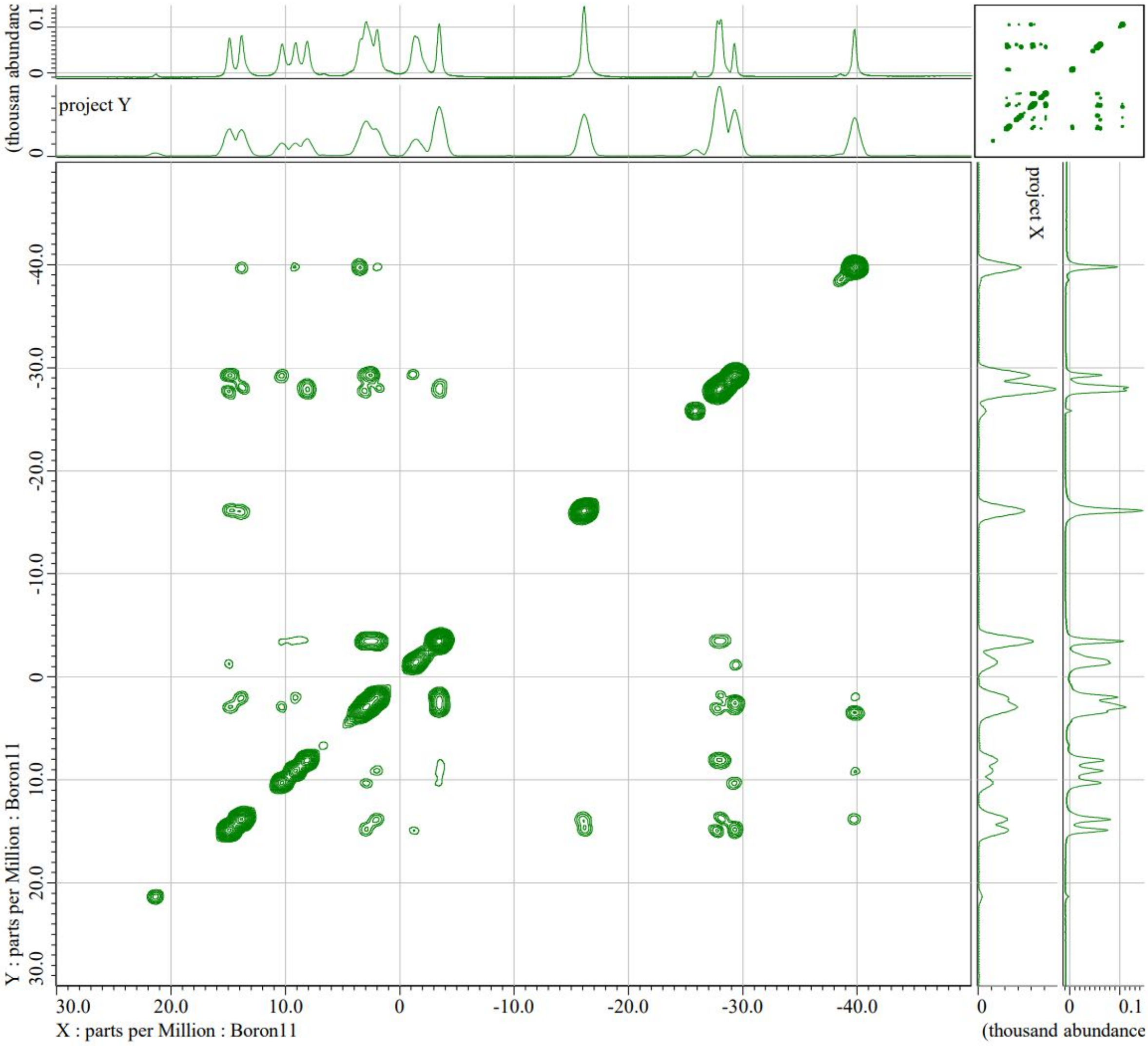

Figure S25. 2-D [^{11}B-^{11}B]-COSY-{^{1}H} NMR spectrum of 4-HS-*syn*-$B_{18}H_{21}$ (**4**)

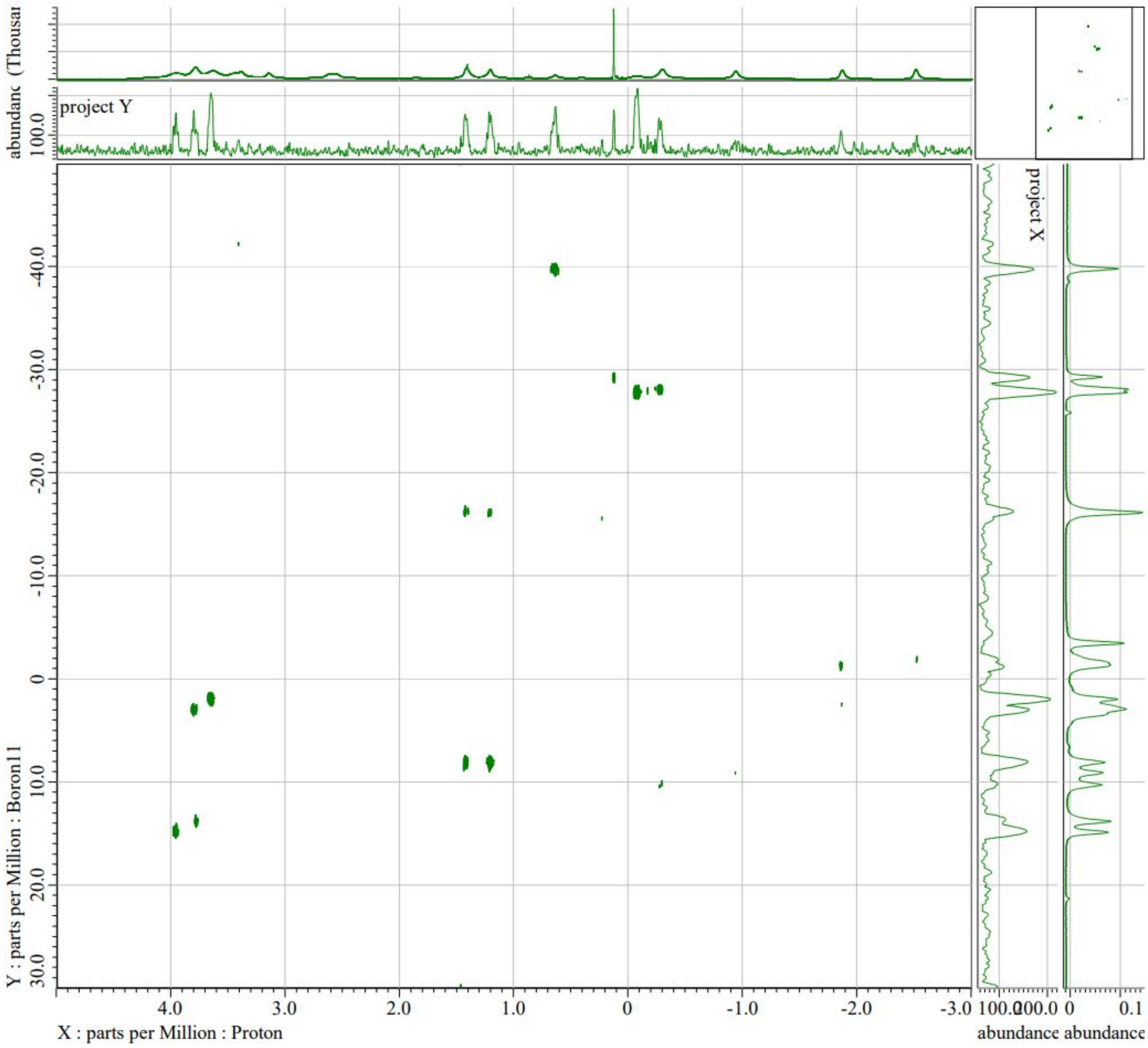

Figure S26. 2-D [^{1}H-^{11}B]-HMQC NMR spectrum of 4-HS-*syn*-$B_{18}H_{21}$ (**4**)

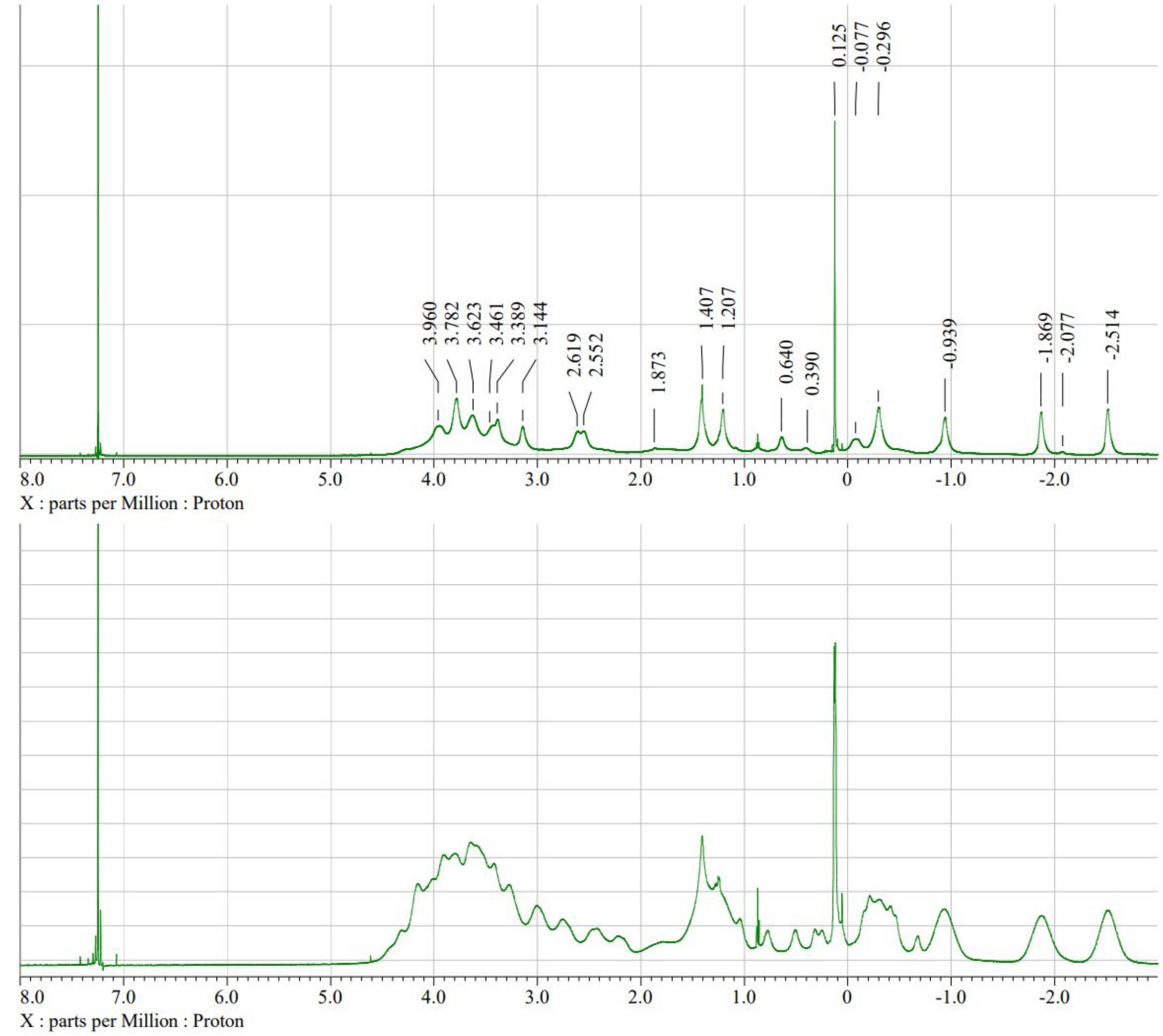

Figure S27. Decoupled (top) $^{1}H\{B\}$ and coupled (bottom) proton NMR spectra of 4-HS-*syn*-$B_{18}H_{21}$ (**4**).

Table S2. Calculated chemical shift in ppm

Atom (Position)	*syn*-$B_{18}H_{22}$	1-HS-*syn*-$B_{18}H_{21}$	2-HS-*syn*-$B_{18}H_{21}$	3-HS-*syn*-$B_{18}H_{21}$	4-HS-*syn*-$B_{18}H_{21}$
B(1)	2.15	11.57	3.49	5.70	3.18
B(2)	-32.27	-33.26	-27.29	-33.15	-32.37
B(3)	14.76	17.93	16.04	21.23	13.57
B(4)	-40.64	-42.84	-42.36	-41.86	-31.81
B(5=5’)	-6.03	-5.04	-4.60	-3.94	-4.67
B(6=6’)	9.93	6.13	7.63	6.08	8.13
B(7)	-18.12	-18.00	-20.19	-20.67	-20.43
B(8)	-0.28	-2.99	-5.89	-4.01	-4.88
B(9)	4.44	-1.74	-2.17	-0.04	-0.13
B(10)	9.32	6.31	7.78	8.96	10.15
B(1’)	2.15	1.50	3.77	4.17	2.27
B(2’)	-32.27	-32.29	30.72	-33.34	-31.57
B(3’)	14.46	13.24	10.71	15.00	14.38
B(4’)	-40.64	-44.07	-37.88	-40.66	-42.81
B(7’)	-18.12	-21.84	-18.52	-19.68	-18.66
B(8’)	-0.28	-6.42	-7.57	-6.42	-4.67
B(9’)	4.44	0.19	5.75	1.73	0.87
B(10’)	9.32	8.75	3.32	9.72	8.23

Table S3. Experimental chemical shift in ppm

syn-$B_{18}H_{22}$	Intensity Ratio	1-HS-*syn*-$B_{18}H_{21}$	Intensity Ratio	3-HS-*syn*-$B_{18}H_{21}$	Intensity Ratio	4-HS-*syn*-$B_{18}H_{21}$	Intensity Ratio
13.552	2	16.698	1	21.354	1	14.898	1
9.664	1	15.022	1	13.479	1	13.846	1
9.321	2	10.938	1	10.300	1	10.3	1
2.987	2	9.628	1	9.664	1	9.126	1
1.666	2	8.348	2	6.631	1	8.098	1
-2.002	2	3.288	1	4.381	1	3.403	1
-4.057	1	2.008	1	3.696	1	2.938	2
-15.870	2	1.063	1	2.718	2	1.984	1
-28.025	2	-0.003	1	0.810	1	-1.318	2
-39.936	2	-2.015	1	-2.174	1	-3.445	1
		-3.844	1	-3.152	1	-16.114	2
		-14.328	1	-15.430	1	-27.805	1
		-16.553	1	-16.750	1	-28.098	1
		-28.073	2	-28.588	1	-29.272	1
		-37.217	1	-29.419	1	-39.765	1
		-40.569	1	-38.517	1		
				-39.447	1		

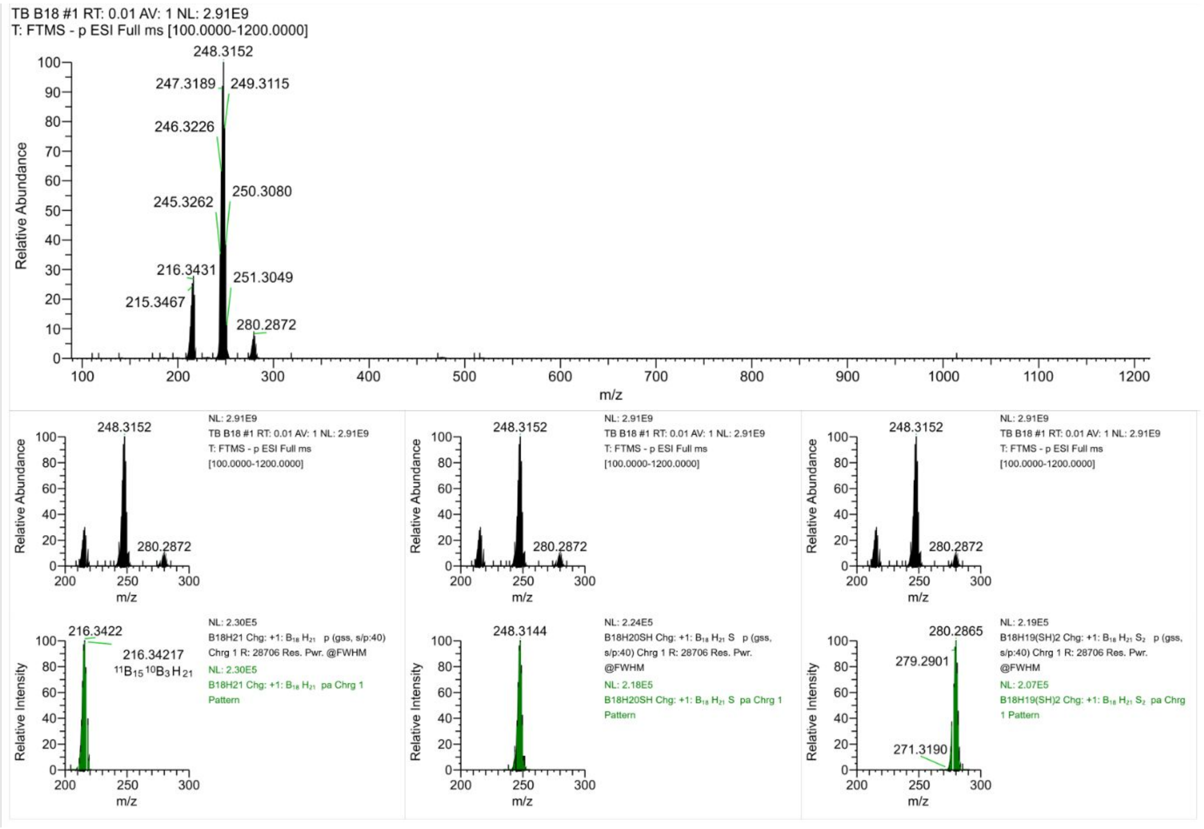

Figure S28. Positive ion mode MS ESI spectra of HS-$B_{18}H_{21}$. (Top) full range m/z spectrum and (bottom) corresponding experimental and theoretical matching of different fragments.

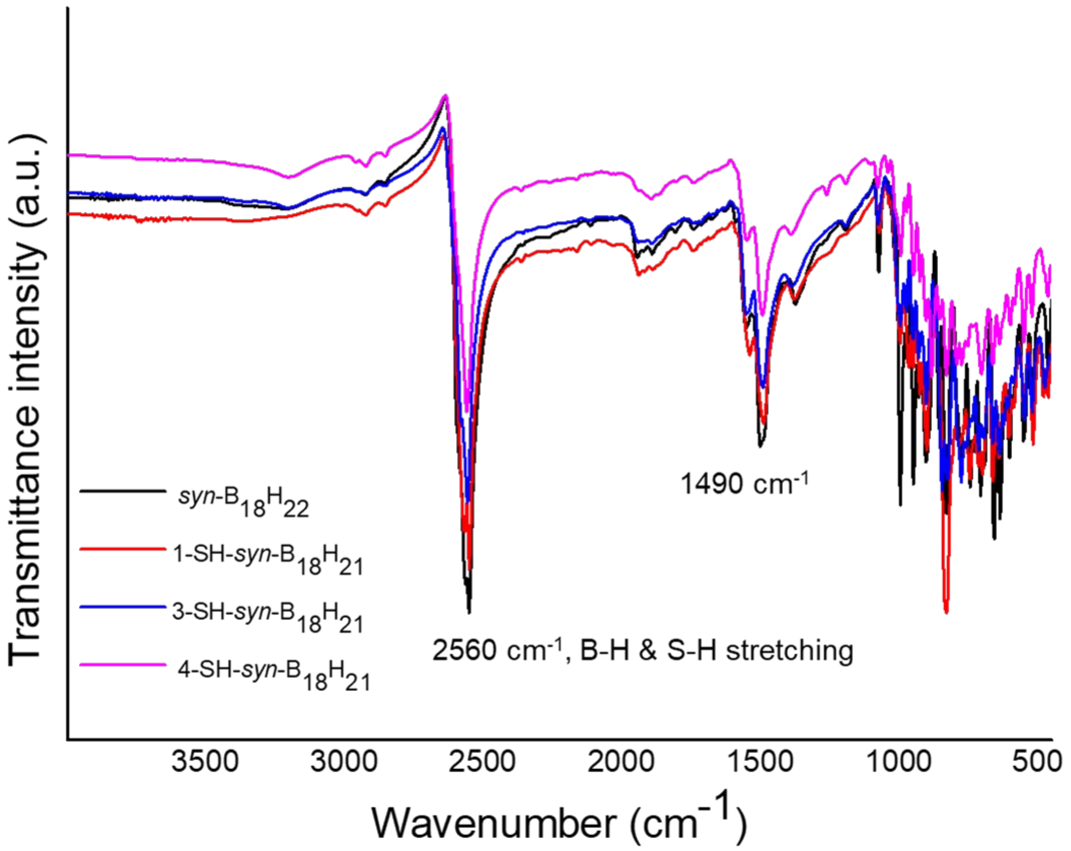

Figure S29. FTIR of HS-*syn*-$B_{18}H_{21}$ isomers and *syn*-$B_{18}H_{22}$.

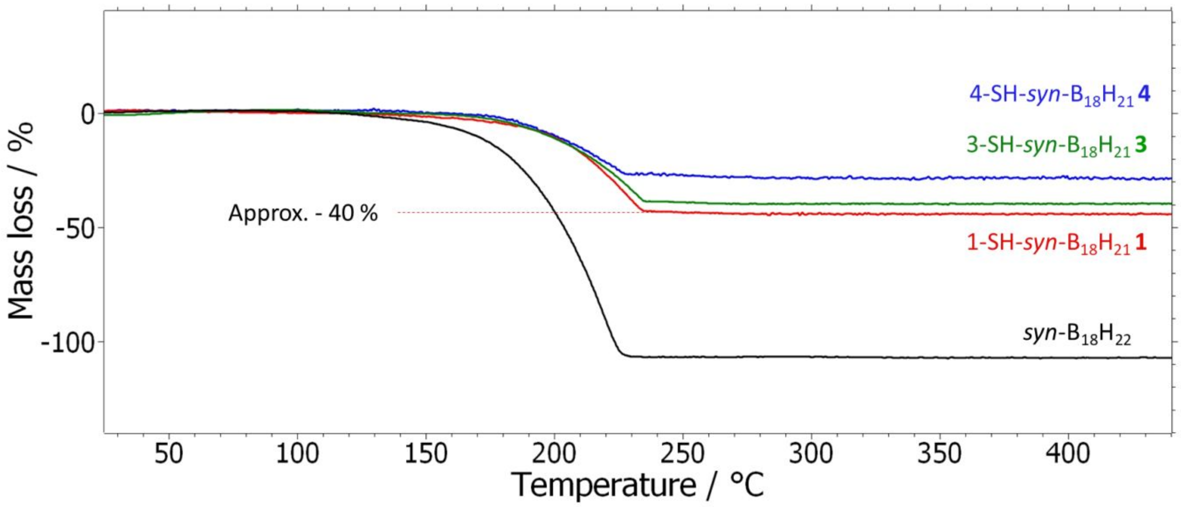

Figure S30. DT analysis showing the volatility of HS-*syn*-$B_{18}H_{21}$ isomers and *syn*-$B_{18}H_{22}$.

Luminescence properties

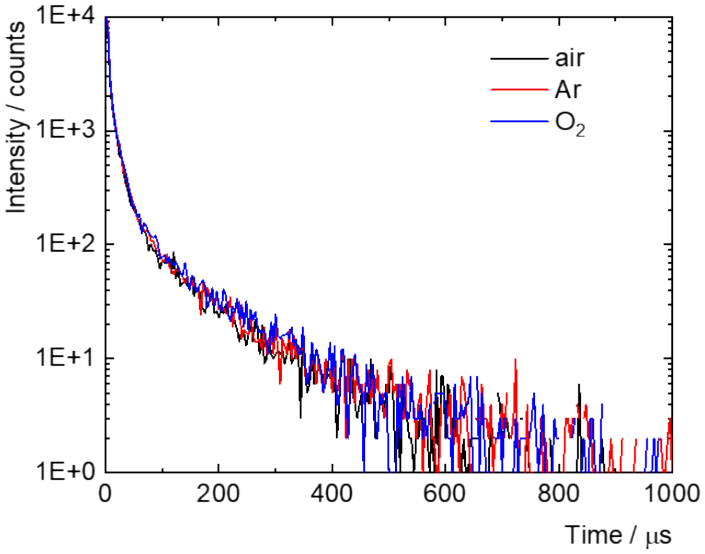

Figure S31. Phosphorescence decay kinetics of powder of 4-HS-*syn*-$B_{18}H_{21}$ in air atmosphere (black), argon atmosphere (red) or oxygen atmosphere (blue) excited at 380 nm, recorded at 470 nm.

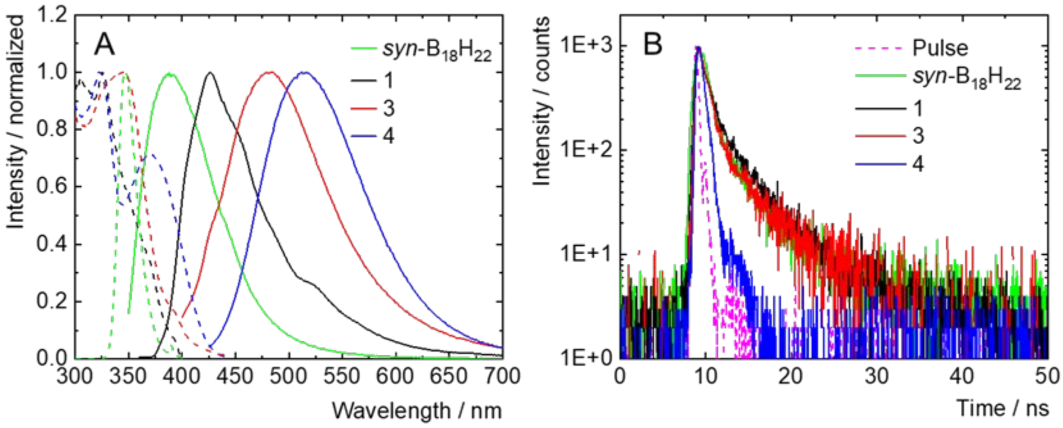

Figure S32. Normalized emission spectra of *syn*-$B_{18}H_{22}$, **1**, **3**, and **4** in air-saturated hexane, excited at 330 nm; normalized excitation spectra (dashed lines) recorded at the maximum of emission, apart for syn-$B_{18}H_{22}$ recorded at 420 nm **(A)**. Fluorescence decay kinetics of *syn*-$B_{18}H_{22}$, **1**, **3**, and **4** in air-saturated hexane, excited at 402 nm, recorded at the maximum of emission, apart for syn-$B_{18}H_{22}$ recorded at 420 nm **(B)**.

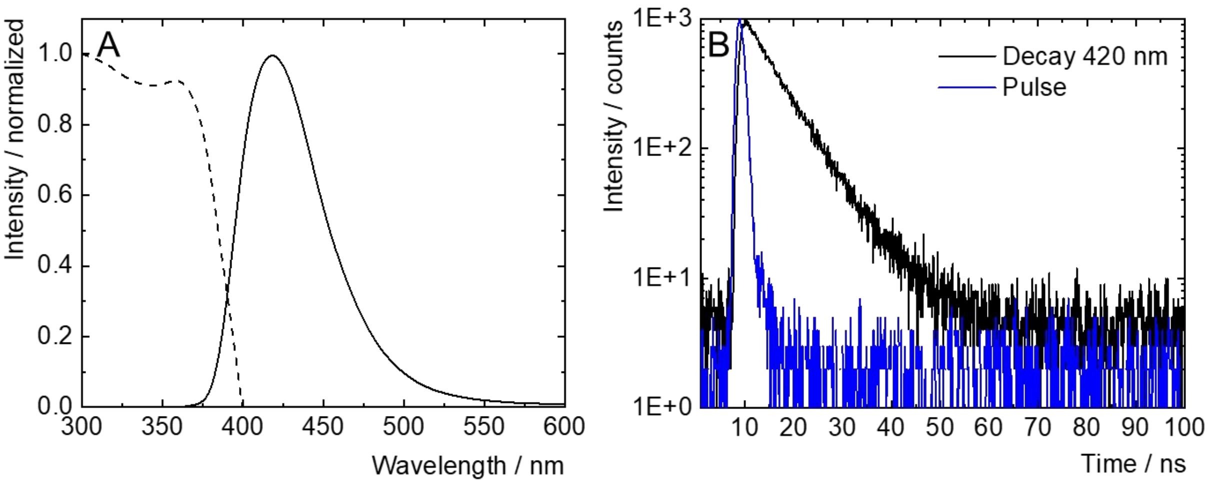

Figure S33. Normalized emission spectra (plain lines) of *anti*-$B_{18}H_{22}$ excited at 340 nm in air atmosphere; normalized excitation spectra (dashed lines) recorded at the maximum of emission **(A)**. Fluorescence decay kinetics of *anti*-$B_{18}H_{22}$ in air atmosphere, excited at 402 nm, recorded at 420 nm **(B)**.

Table S4. Photophysical properties of *syn*-$B_{18}H_{22}$ and the thiolated boranes in hexane at room temperature.[a]

Sample	λ_L / nm	ϕ_L	τ_L / ns
Syn-$B_{18}H_{22}$	390	<0.01	1.1
1	426	<0.01	1.4
3	483	<0.01	1.0
4	515	<0.01	0.3

[a] λ_L - luminescence maximum (λ_{exc} = 380 nm); τ_L –amplitude average lifetimes (λ_{exc} = 380 nm) measured at the maximum of emission; Φ_L luminescence quantum yields (λ_{exc} = 380 nm, experimental error of Φ_L is ± 0.01); [b] shoulder; [c] after grinding

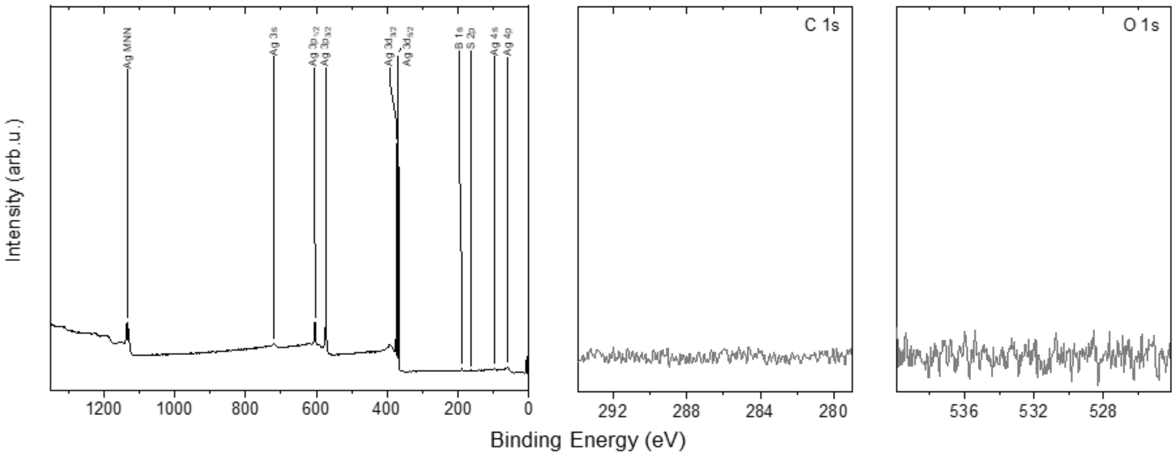

Figure S34. Overview X-ray photoelectron spectrum (A) as well as high-resolution C 1s (B) and O 1s (C) spectra of the formed [4-HS-syn-$B_{18}H_{21}$] **4** SAM on Ag. The data clearly show only the presence of boron, sulfur and silver signals in the sample. No carbon or oxygen signals are detected demonstrating a high quality of the formed SAM.

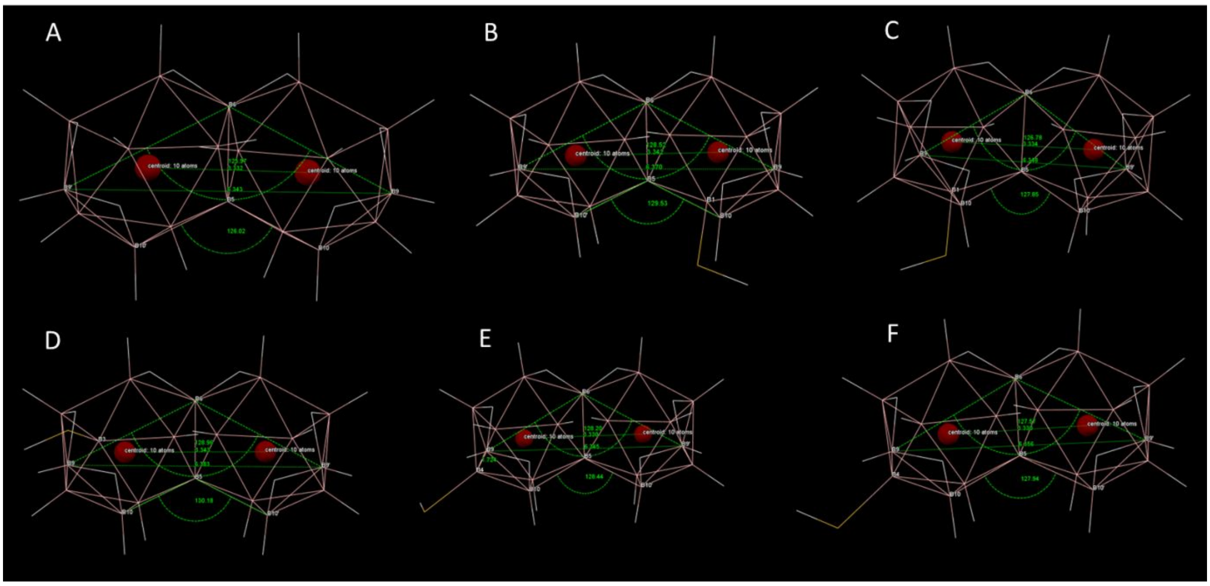

Figure S35. B-B-B angle, bond and centroid(c1-c2) distance of syn-$B_{18}H_{22,}$ **1**, **3**, and **4** ismores polymorphs (A, B, C, D, E, and F respectively).

Table S5. Bond distance comparison of all the isomers with *syn*-$B_{18}H_{22}$

Atom1	Atom2	*syn*-$B_{18}H_{22}$	1-HS-*syn*-$B_{18}H_{21}$ **PM1a**	1-HS-*syn*-$B_{18}H_{21}$ **PM1b**	1-HS-*syn*-$B_{18}H_{21}$	4-HS-*syn*-$B_{18}H_{21}$ **PM4a**	4-HS-*syn*-$B_{18}H_{21}$ **PM4b**
B6	B5	1.810(2)	1.808(3)	1.815(3)	1.810(3)	1.803(2)	1.799(2)
B6	B2'	1.735(2)	1.734(3)	1.735(4)	1.741(3)	1.737(2)	1.737(2)
B6	B7	1.789(2)	1.783(3)	1.793(4)	1.795(2)	1.797(2)	1.785(2)
B6	B2	1.729(2)	1.737(4)	1.736(4)	1.740(3)	1.743(2)	1.737(3)

B6	B7'	1.792(2)	1.798(3)	1.796(5)	1.796(3)	1.781(2)	1.783(2)
B6	H1b0607'	1.28(2)	1.21(2)	1.23(3)	1.27(2)	1.30(1)	1.29(2)
B6	H1b0607	1.28(1)	1.30(2)	1.28(3)	1.30(2)	1.28(1)	1.30(2)
B5	B1'	1.777(2)	1.768(3)	1.776(4)	1.768(2)	1.775(2)	1.775(2)
B5	B2'	1.837(2)	1.834(4)	1.841(4)	1.829(2)	1.830(2)	1.833(3)
B5	B10	1.985(2)	1.980(3)	1.973(4)	1.995(3)	1.991(2)	1.973(2)
B5	B1	1.771(2)	1.793(4)	1.778(4)	1.771(2)	1.775(2)	1.779(2)
B5	B2	1.843(2)	1.840(3)	1.841(4)	1.831(2)	1.834(2)	1.839(2)
B5	B10'	1.981(2)	1.988(3)	1.977(4)	1.990(2)	1.976(2)	1.976(2)
B1'	B2'	1.788(2)	1.791(3)	1.793(3)	1.793(3)	1.793(2)	1.791(2)
B1'	B10'	1.749(2)	1.746(4)	1.747(3)	1.749(3)	1.754(2)	1.745(3)
B1'	B3'	1.789(2)	1.787(3)	1.792(4)	1.786(3)	1.790(2)	1.785(2)
B1'	B4'	1.797(2)	1.796(3)	1.792(4)	1.794(3)	1.796(2)	1.795(2)
B1'	H1b1'	1.05(2)	1.05(2)	1.09(2)	1.06(2)	1.05(1)	1.06(2)
B2'	B3'	1.763(2)	1.757(3)	1.758(5)	1.765(2)	1.762(2)	1.764(2)
B2'	B7'	1.803(2)	1.794(3)	1.793(4)	1.801(3)	1.793(2)	1.796(2)
B2'	H1b2'	1.05(1)	1.05(2)	1.10(3)	1.05(2)	1.07(1)	1.06(2)
B10	B1	1.746(2)	1.760(4)	1.761(3)	1.754(3)	1.744(2)	1.752(2)
B10	B9	1.781(2)	1.784(3)	1.778(4)	1.781(2)	1.791(2)	1.786(3)
B10	B4	1.792(2)	1.793(4)	1.792(5)	1.787(2)	1.788(2)	1.789(3)
B10	H1b0910	1.25(2)	1.27(3)	1.26(3)	1.31(2)	1.26(1)	1.24(2)
B10	H1b10	1.08(2)	1.09(3)	1.07(4)	1.06(2)	1.05(1)	1.07(2)
B7	B8	1.953(2)	1.949(3)	1.953(4)	1.923(3)	1.946(2)	1.951(2)
B7	B2	1.796(2)	1.793(3)	1.796(3)	1.801(3)	1.796(2)	1.799(3)
B7	B3	1.758(2)	1.758(4)	1.761(4)	1.770(3)	1.760(2)	1.760(3)
B7	H1b7	1.05(2)	1.03(3)	1.09(4)	1.10(2)	1.08(1)	1.09(2)
B7	H1b0607	1.26(2)	1.22(3)	1.20(3)	1.22(2)	1.22(1)	1.22(2)
B1	B2	1.787(2)	1.798(3)	1.794(4)	1.796(3)	1.790(2)	1.793(2)
B1	B4	1.798(2)	1.797(3)	1.800(4)	1.805(3)	1.805(2)	1.795(2)
B1	B3	1.787(2)	1.794(3)	1.795(4)	1.790(2)	1.795(2)	1.791(2)
B1	H1b1	1.08(2)			1.07(2)	1.07(1)	1.08(2)
B8	B9	1.800(2)	1.798(3)	1.803(4)	1.802(3)	1.805(2)	1.812(3)
B8	B4	1.808(2)	1.804(4)	1.804(4)	1.808(3)	1.813(2)	1.809(3)
B8	B3	1.763(2)	1.761(4)	1.769(4)	1.774(3)	1.767(2)	1.762(3)
B8	H1b8	1.08(2)	1.04(2)	1.07(3)	1.07(2)	1.07(1)	1.07(2)
B8	H1b0809	1.23(2)	1.26(3)	1.26(4)	1.24(2)	1.25(1)	1.23(2)
B9	B4	1.726(2)	1.721(4)	1.727(5)	1.729(3)	1.724(2)	1.725(3)
B9	H1b0910	1.27(2)	1.27(3)	1.21(4)	1.19(2)	1.24(1)	1.26(2)
B9	H1b9	1.07(1)	1.05(3)	1.07(3)	1.02(2)	1.05(2)	1.09(2)
B9	H1b0809	1.27(2)	1.34(3	1.32(4)	1.27(2)	1.27(2)	1.27(2)
B2	B3	1.763(2)	1.767(3)	1.767(5)	1.756(2)	1.761(2)	1.756(2)
B2	H1b2	1.05(1)	1.05(2)	1.11(2)	1.05(2)	1.07(1)	1.04(2)
B10'	B4'	1.786(2)	1.782(5)	1.779(4)	1.783(3)	1.785(2)	1.785(3)
B10'	B9'	1.783(2)	1.788(4)	1.778(4)	1.788(3)	1.784(2)	1.785(2)
B10'	H1b10'	1.09(2)	1.06(3)	1.02(2)	1.04(2)	1.10(1)	1.07(2
B10'	H1b0910'	1.27(2)	1.28(3)	1.27(3)	1.21(2)	1.25(1)	1.27(2)
B4	B3	1.782(2)	1.785(3)	1.789(3)	1.771(3)	1.775(2)	1.787(2)
B4	H1b4	1.03(2)	1.08(3)	1.03(4)	1.06(2)		
B3'	B7'	1.762(2)	1.762(3)	1.759(4)	1.761(2)	1.761(2)	1.762(2)
B3'	B4'	1.780(2)	1.774(4)	1.775(3)	1.778(3)	1.780(2)	1.774(3)
B3'	B8'	1.767(2)	1.759(3)	1.760(3)	1.761(3)	1.765(2)	1.761(2)
B3'	H1b3'	1.07(2)	1.04(3)	1.01(4)	1.07(2)	1.09(1)	1.08(2)
B7'	B8'	1.959(2)	1.950(3)	1.965(4)	1.954(2)	1.952(2)	1.956(2)
B7'	H1b7'	1.07(2)	1.03(3)	1.05(3)	1.08(2)	1.07(1)	1.08(2)
B7'	H1b0607'	1.23(2)	1.22(3)	1.22(3)	1.22(2)	1.23(1)	1.23(2)
B3	H1b3	1.05(2)	1.13(3)	1.13(3)		1.08(1)	1.07(2)
B4'	B8'	1.811(2)	1.802(3)	1.800(3)	1.810(3)	1.804(2)	1.801(2)
B4'	B9'	1.725(2)	1.724(4)	1.729(3)	1.727(3)	1.727(2)	1.723(3)
B4'	H1b4'	1.05(2)	1.27(3)	1.05(3)	1.06(2)	1.09(1)	1.15(2)

B8'	B9'	1.806(2)	1.796(4)	1.798(3)	1.796(3)	1.794(2)	1.794(3)
B8'	H1b8'	1.08(2)	1.08(3)	1.02(3)	1.03(2)	1.06(2)	1.03(2)
B8'	H1b0809'	1.26(2)	1.20(3)	1.24(4)	1.28(2)	1.25(1)	1.26(2)
B9'	H1b9'	1.07(1)	1.02(2)	1.09(3)	1.08(2)	1.07(2)	1.05(2)
B9'	H1b0809'	1.29(2)	1.29(2)	1.30(3)	1.30(2)	1.30(2)	1.26(2)
B9'	H1b0910'	1.25(2)	1.23(3)	1.23(4)	1.18(2)	1.23(1)	1.24(2)

Powder X-ray diffraction and Rietveld refinement

XRPD was investigated on a SmartLab Rigaku powder diffractometer with a rotating Cu anode at room temperature using a HyPix-3000 detector. It was confirmed that no phase of HS-*syn*-$B_{18}H_{21}$ was missed. The data were processed using the HighScore Plus program from PANalytical. The results of the phase analysis are in following Figures S36 to S40.

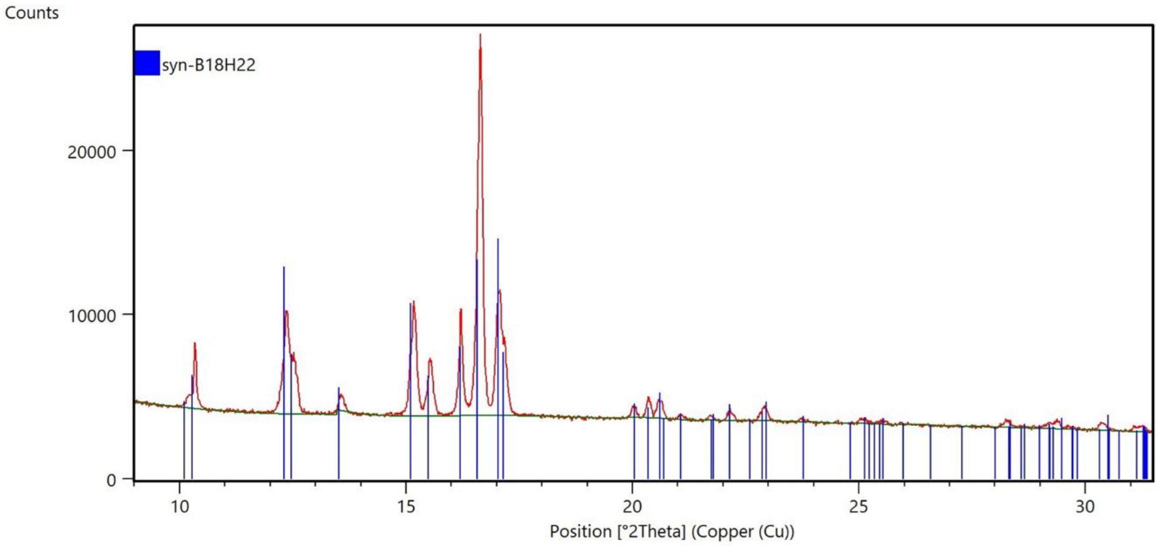

Figure S36. Rietveld refinement of the powdered sample of *syn*-$B_{18}H_{22}$.

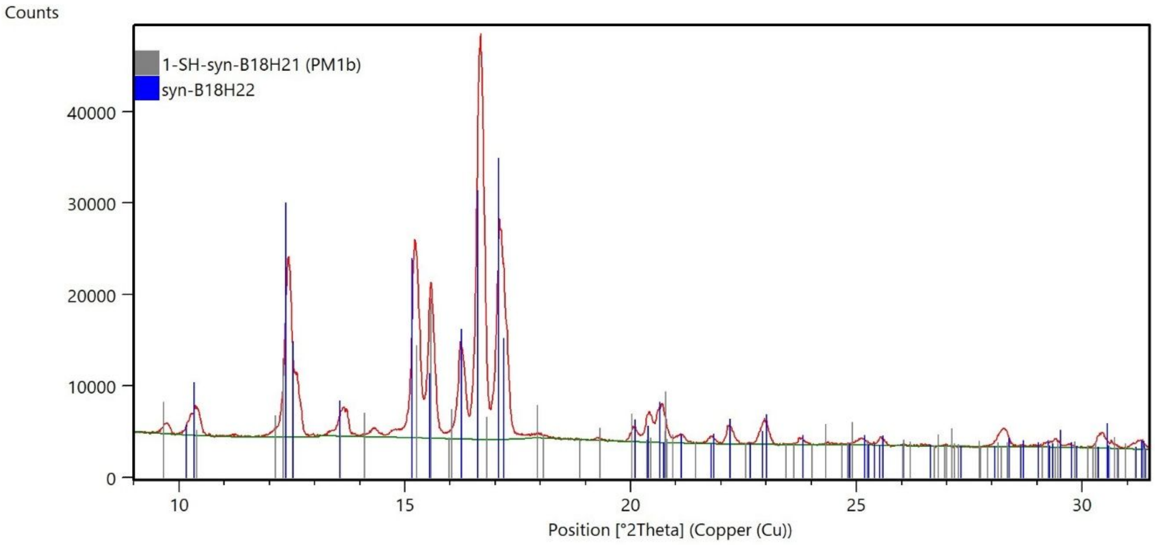

Figure S37. Rietveld refinement of the powdered sample of1-HS-*syn*-$B_{18}H_{21}$ (PM1b) and *syn*-$B_{18}H_{22}$.

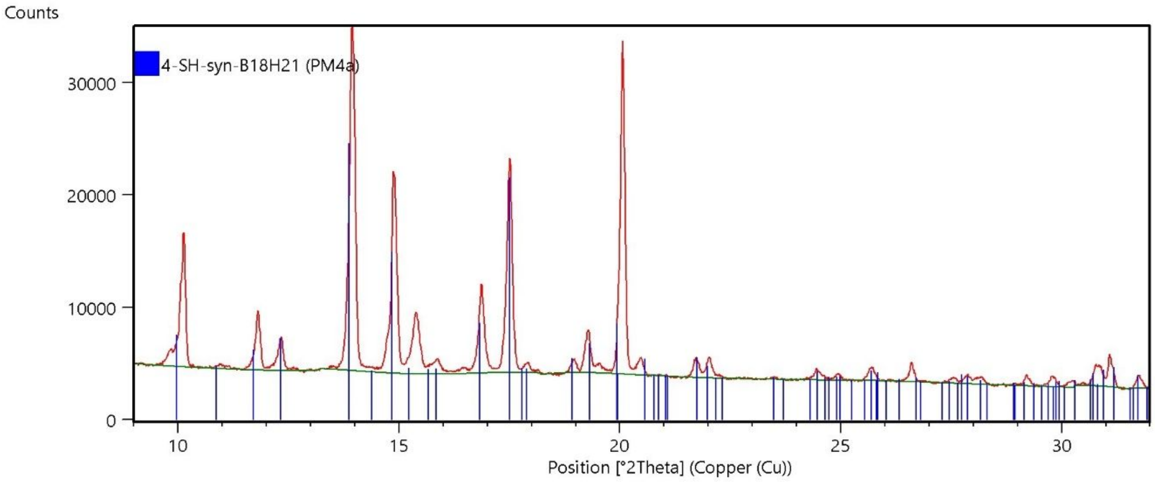

Figure S38. Rietveld refinement of the powdered sample of 4-HS-*syn*-$B_{18}H_{21}$ (PM4a).

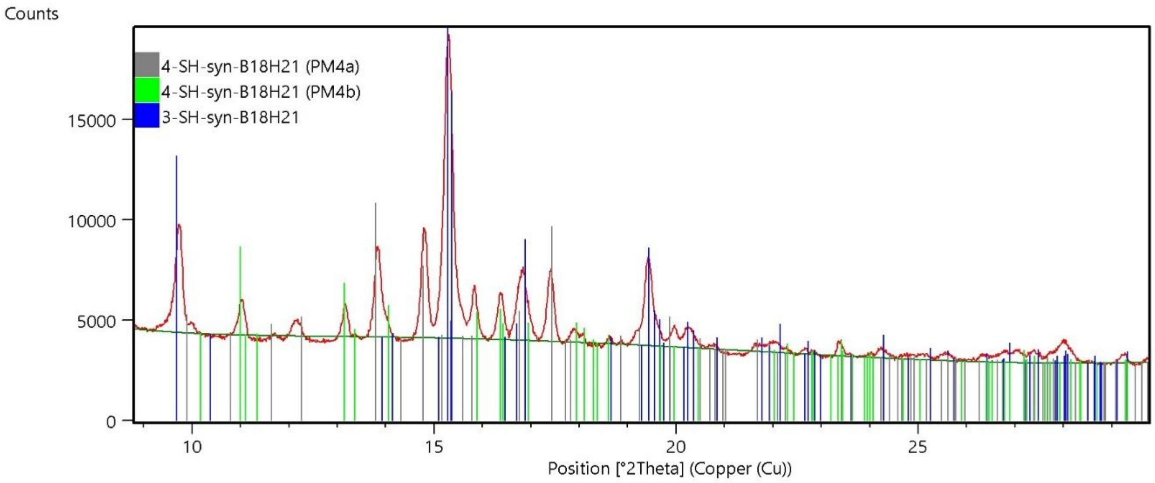

Figure S39. Rietveld refinement of the powdered sample of 4-HS-*syn*-$B_{18}H_{21}$ (PM4a and PM4b) and *syn*-$B_{18}H_{22}$.

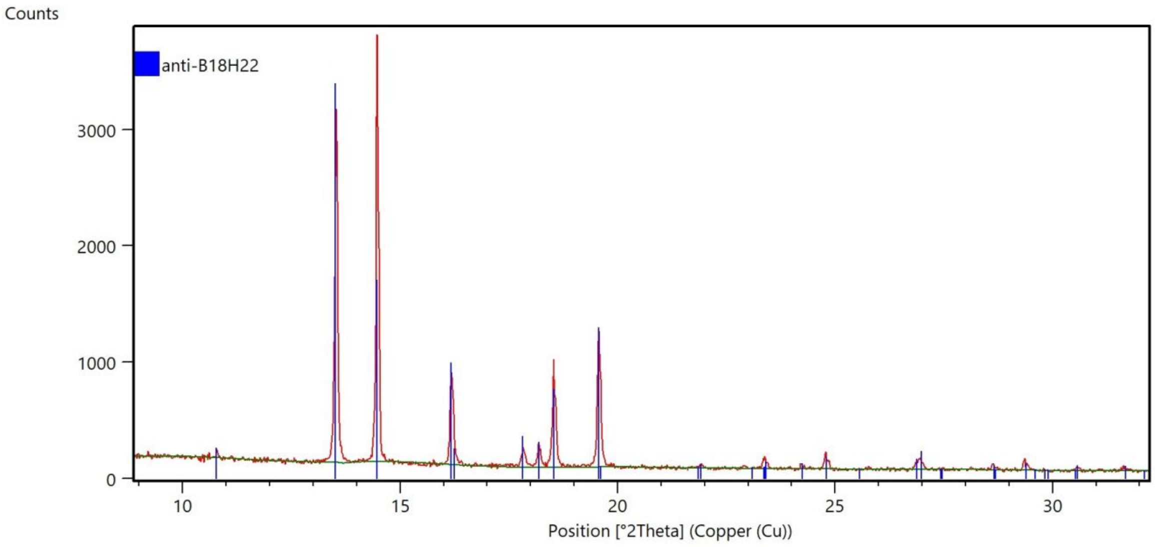

Figure 40. Powder X-ray diffraction pattern (PXRD) of *anti*-$B_{18}H_{22}$ (obtained at room temperature using a PANalytical Empyrean diffractometer equipped with a conventional Cu X-ray tube and PIXcel detector). The data were processed using the HighScore Plus program from PANalytical, and the obtained diffraction pattern fits perfectly to the single-crystal simulated one (CCDC number 602765).

Single crystal X-ray analysis

The X-ray diffraction data of samples were collected with Rigaku OD Supernova using Atlas S2 CCD detector and mirror collimated Cu-Kα (λ = 1.54184 Å) from micro-focused sealed X-ray tube. The samples were cooled to 95 K during the measurement. Integration of the CCD images, absorption correction and scaling were done by program CrysAlisPro 1.171.41.123a (Rigaku Oxford Diffraction, 2022). Crystal structures were solved by charge flipping with program SUPERFLIP and refined with the Jana2020 program package by full-matrix least-squares technique on F^2. All hydrogen atoms were visible in difference Fourier maps and positions of all of them were

refined freely. The isotropic atomic displacement parameters of hydrogen atoms were evaluated as 1.2Ueq of the parent atom. ORTEP diagrams (Figure S41 to S46) were made in Diamond 4.5.1. Obtained crystallographic data are in Tables S6 to S13

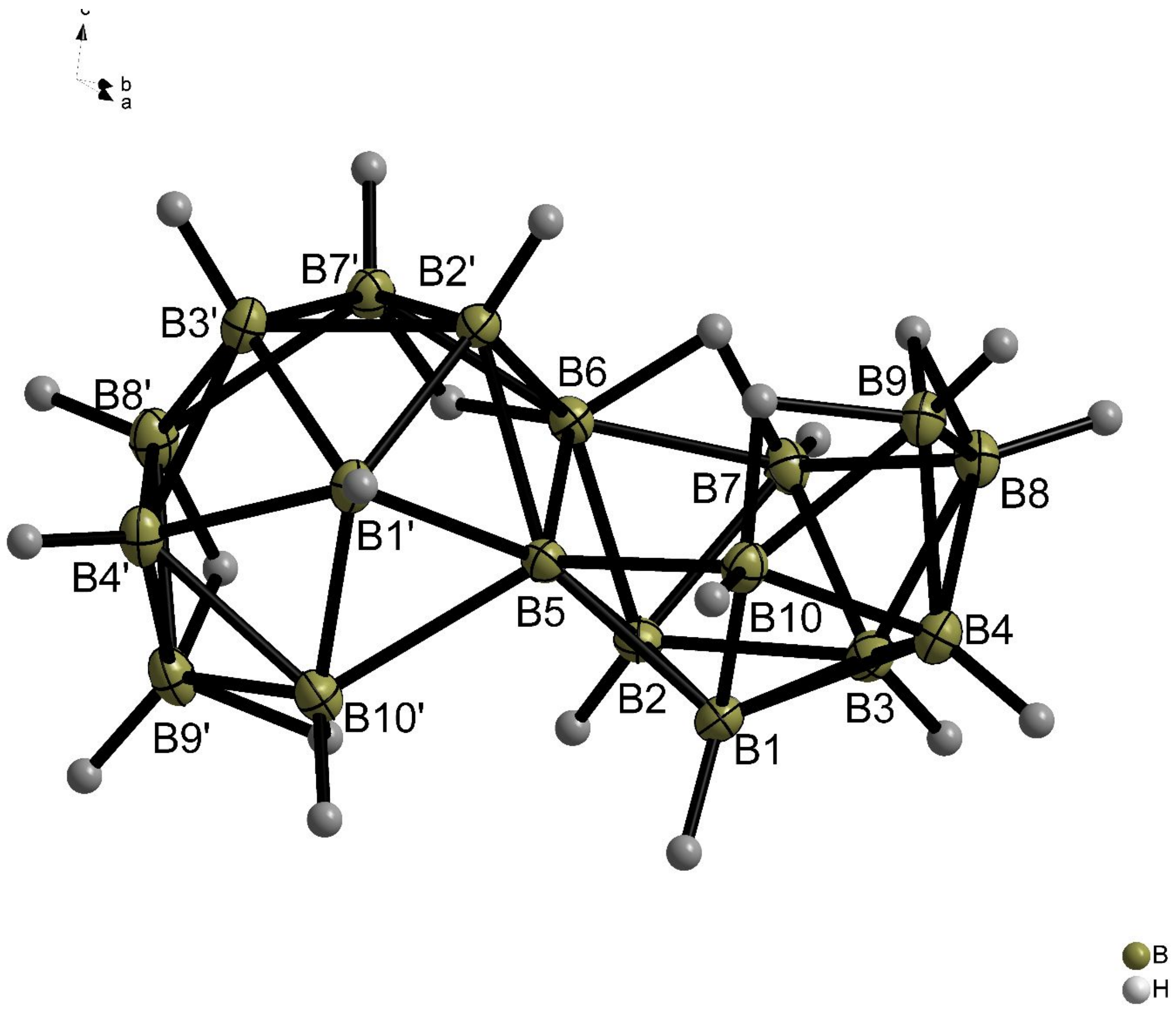

Figure S41. ORTEP structure of *syn*-$B_{18}H_{22}$ isomer having 50% thermal ellipsoid probability.

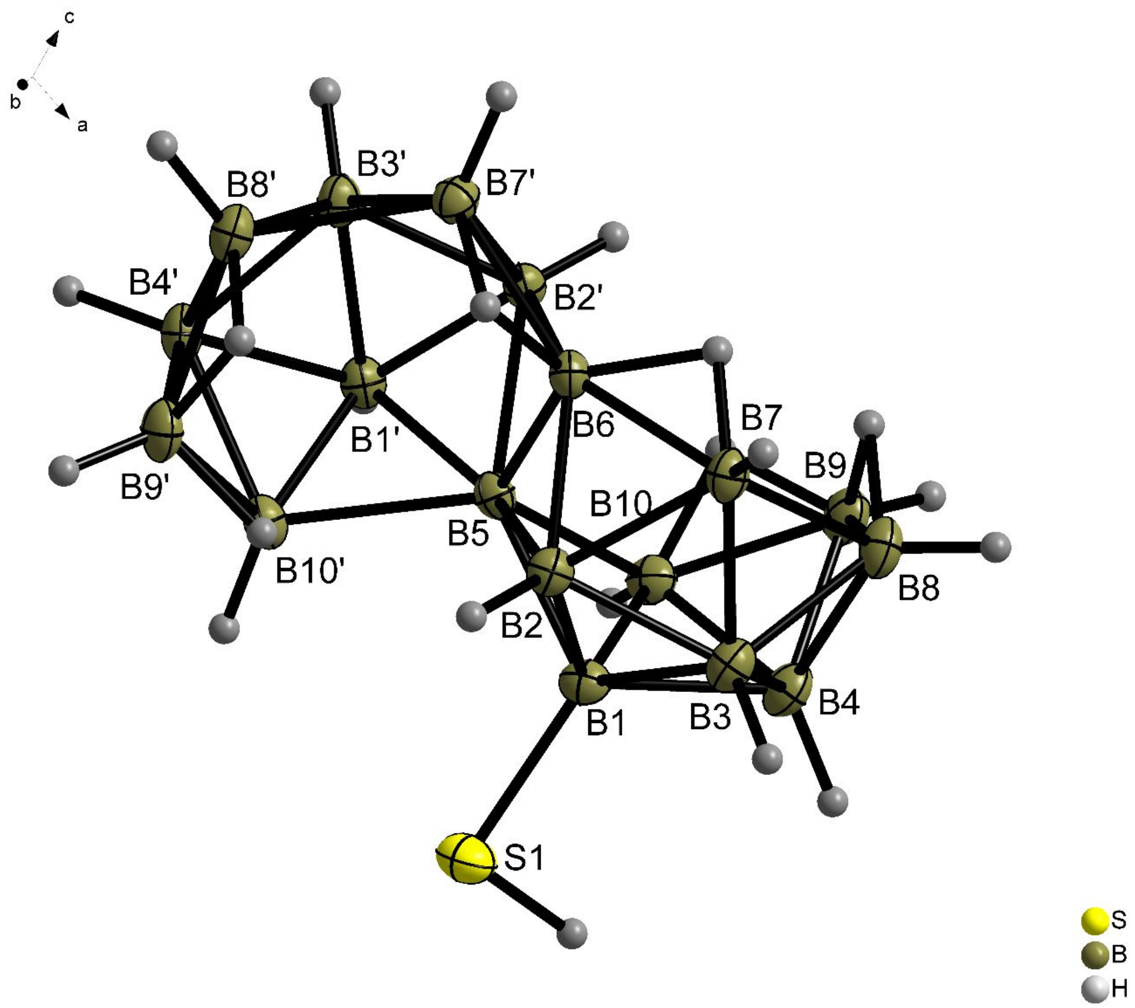

Figure S42. ORTEP structure of 1-HS-*syn*-$B_{18}H_{21}$ PM1a isomer having 50% thermal ellipsoid probability.

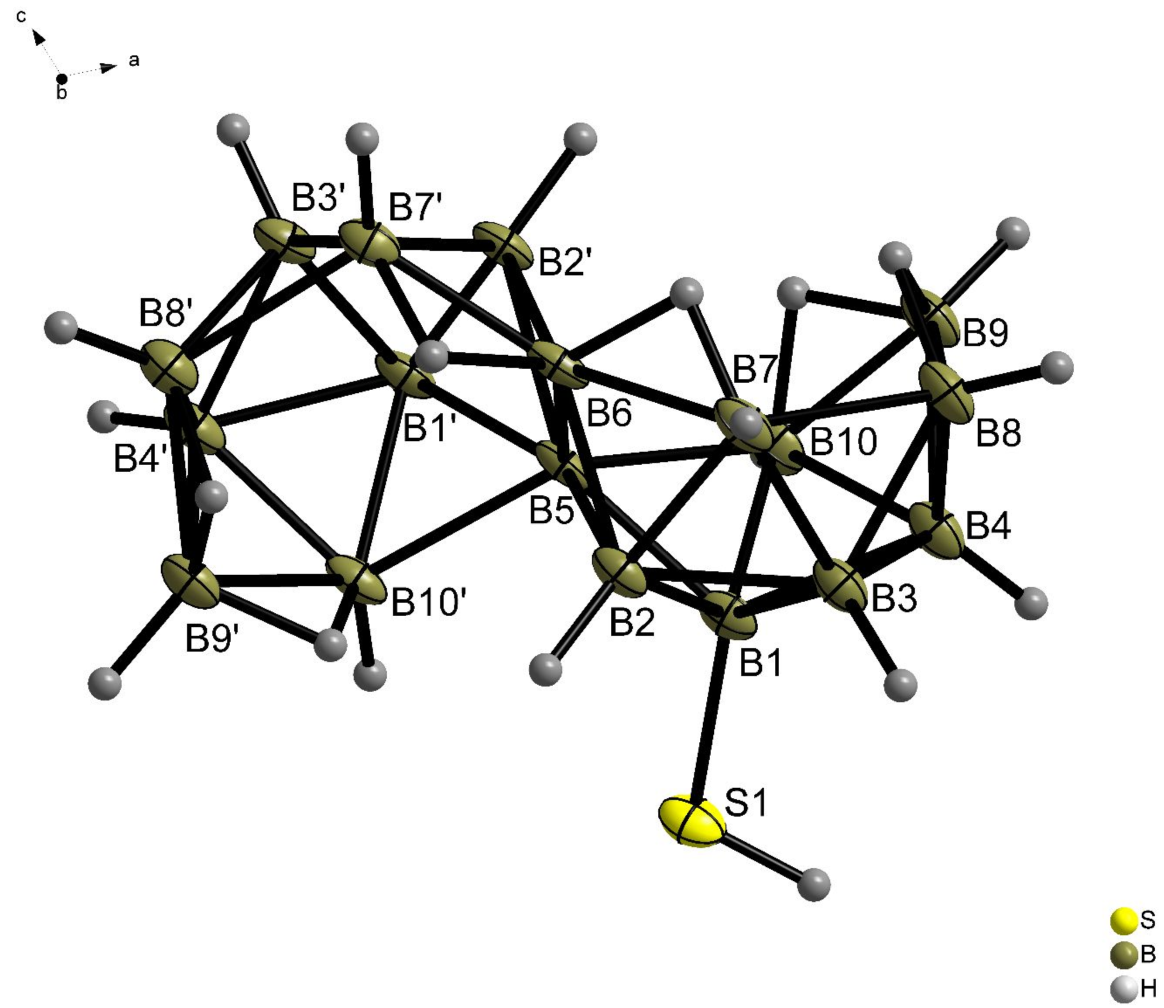

Figure S43. ORTEP structure of 1-HS-*syn*-$B_{18}H_{21}$ PM1b isomer having 50% thermal ellipsoid probability.

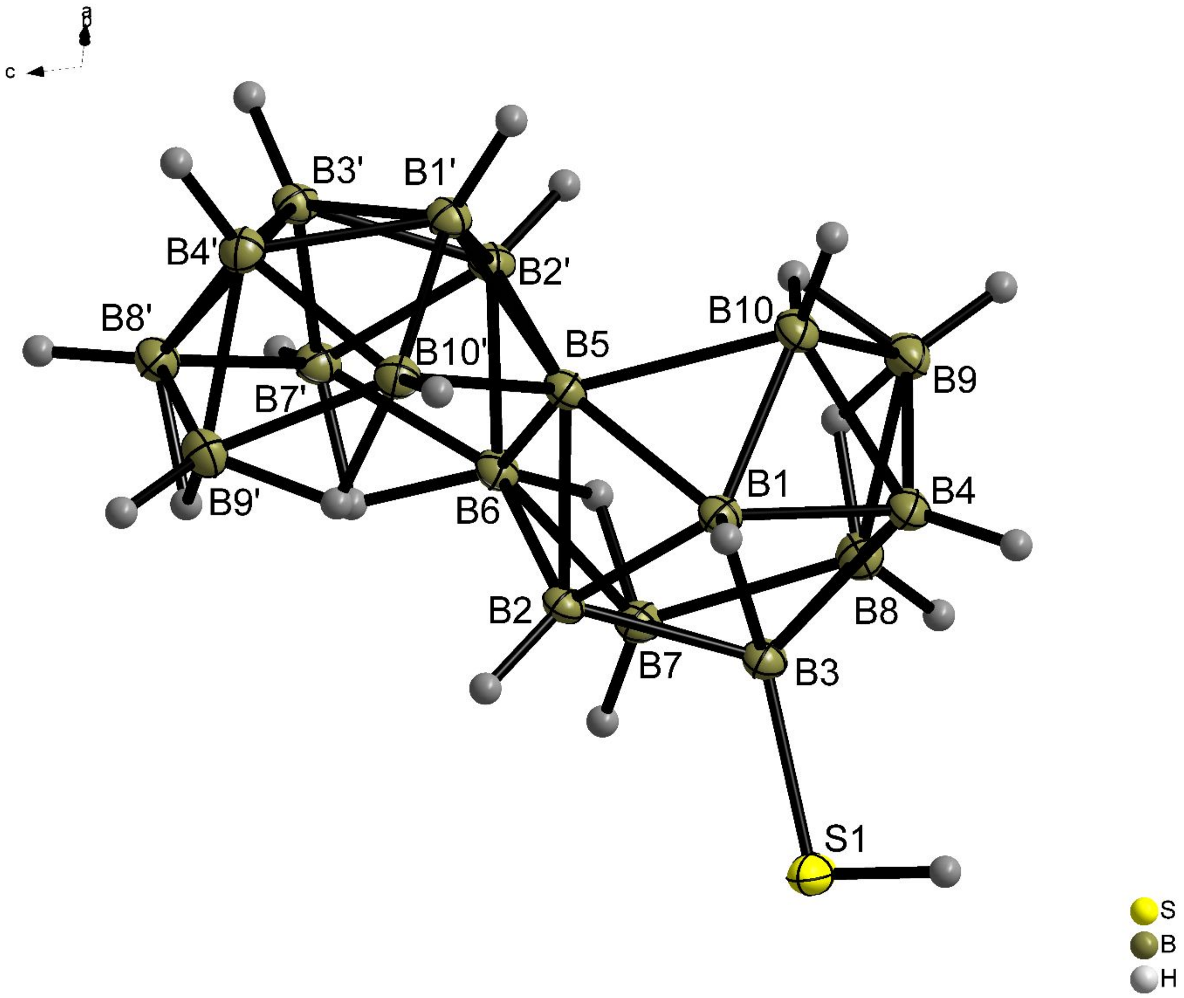

Figure S44. ORTEP structure of 3-HS-*syn*-$B_{18}H_{21}$ isomer having 50% thermal ellipsoid probability.

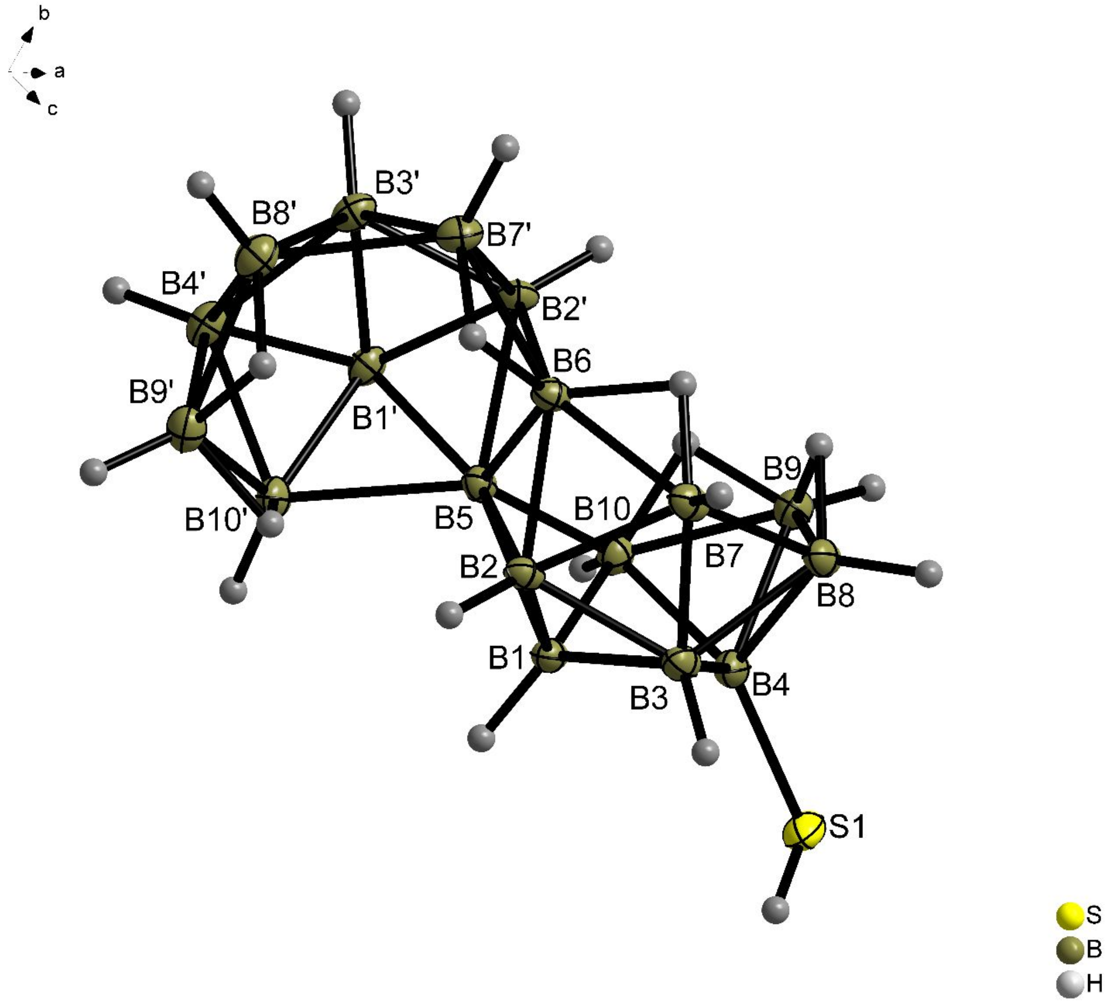

Figure S45. ORTEP structure of 4-HS-*syn*-$B_{18}H_{21}$ PM4a isomer having 50% thermal ellipsoid probability.

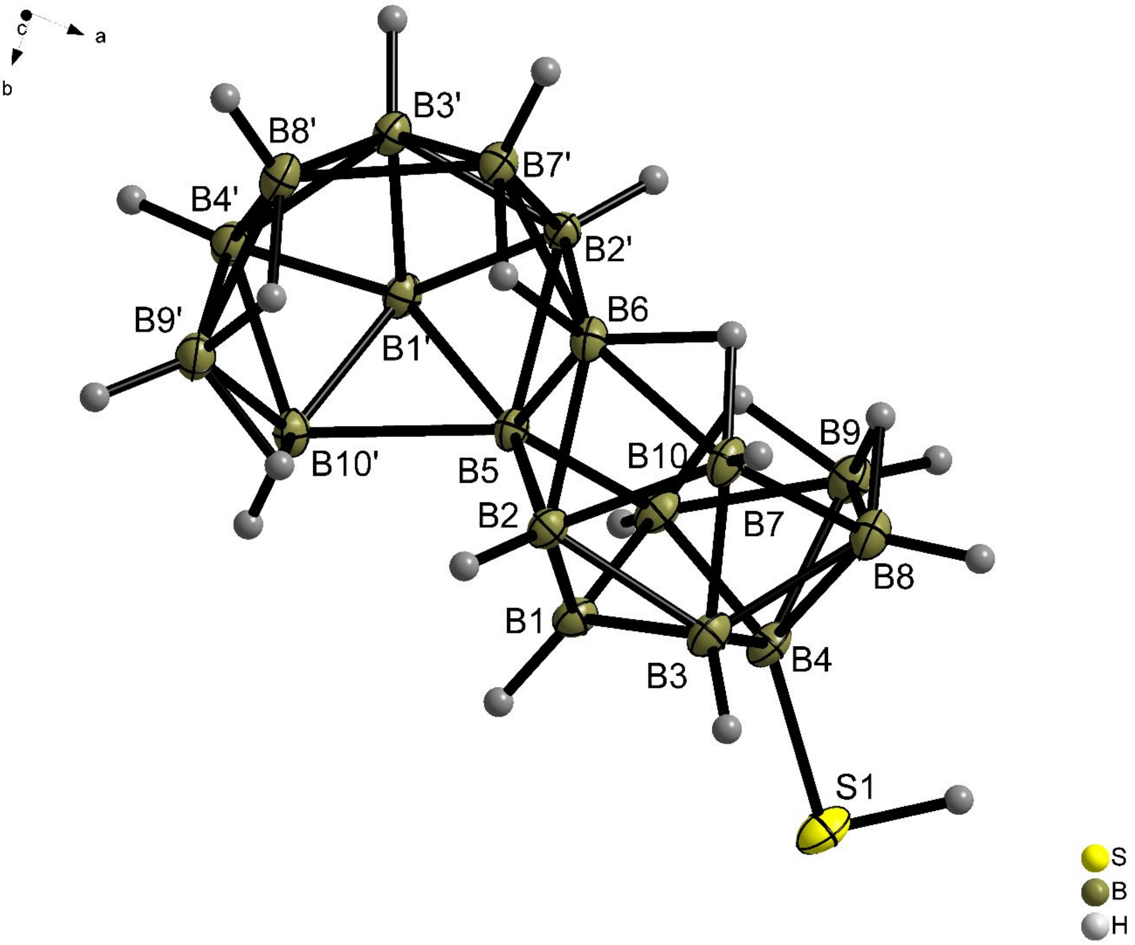

Figure S46. ORTEP structure of 4-HS-*syn*-$B_{18}H_{21}$ PM4b isomer having 50% thermal ellipsoid probability.

Table S6. **Crystallographic collection and refinement data for *syn*-$B_{18}H_{22}$ measured at 95 K**.

	syn-$B_{18}H_{22}$ – at 95 K.
CCDC	2262308
Empirical formula	B_{18} H_{22}
Diffractometer	four-cycle diffractometer<br>Cryostream, AtlasS2, Rigaku OD Supernova
M_r /g mol^{-1}	216.8 (formula weight)

	syn-$B_{18}H_{22}$ – at 95 K.
T/K	95
Wavelength / Å	1.54184
Crystal system	monoclinic
Space group	$P2_1/c$
a/Å	9.108(2)
b/Å	13.050(3)
c/Å	12.432(2)
α/deg	90
β/deg	109.94(3)
γ/deg	90
V/$Å^3$	1389.1(6)
Z	4
Calc. density/g cm^{-3}	1.0364
μ mm^{-1}	0.219
F(000)	448
Crystal size /mm^3	0.090 x 0.080 x 0.040
θ range /°	5.1 – 73.0
Index ranges /*hkl*	-10, 11, -16, 16, -15, 8
Reflections collected (R_{int})	7784 (0.042)
Independent reflections	2740

	syn-$B_{18}H_{22}$ – at 95 K.
Completeness /% to θ /°	98, 73.53
Absorption correction	multiscan
Max. and min. transmission	0. 76, 1
Data / restraints / constraints / parameters	2740/ 0 / 22 / 229
Goodness-of-fit on F^2	1.51 [(1)]
$R1$, $wR2$ [I>3σ(I)]	0.0397, 0.0857
$R1$, $wR2$ (all data)	0.0573, 0.0920
Largest diff. peak and hole, eÅ^{-3}	0.15, -0.15

Table S7. Crystallographic collection and refinement data for *syn*-$B_{18}H_{22}$ measured at room temperature.

	syn-$B_{18}H_{22}$ – at RT.
CCDC	2265132
Empirical formula	B_{18} H_{22}
Diffractometer	four-cycle diffractometer<br>Cryostream, AtlasS2, Rigaku OD Supernova
M_r /g mol^{-1}	216.8 (formula weight)
T/K	300
Wavelength / Å	0.71073

	syn-$B_{18}H_{22}$ – at RT.
Crystal system	monoclinic
Space group	$P2_1/c$
a/Å	9.1929(11)
b/Å	13.1716(14)
c/Å	12.5191(14)
α/deg	90
β/deg	109.759(11)
γ/deg	90
V/Å^3	1426.63
Z	4
Calc. density/g cm^{-3}	1.009
μ mm^{-1}	0.04
F(000)	448
Crystal size /mm^3	0.21 × 0.12 × 0.07
θ range /°	2.8 – 25.5
Index ranges /*hkl*	-9, 12, -18, 13, -16, 14
Reflections collected (R_{int})	6140 (0.035)
Independent reflections	3293
Completeness /% to θ /°	98, 26.86
Absorption correction	multiscan

	syn-$B_{18}H_{22}$ – at RT.
Max. and min. transmission	0. 69, 1
Data / restraints / constraints / parameters	3293/ 0 / 22 / 229
Goodness-of-fit on F^2	1.31 (1)
$R1$, $wR2$ [I>3σ(I)]	0.0585, 0.1231
$R1$, $wR2$ (all data)	0.1146, 0.1472
Largest diff. peak and hole, eÅ^{-3}	0.13, -0.12

Table S8. Crystallographic collection and refinement data for 1-HS-*syn*-$B_{18}H_{21}$ (PM1a).

**POLYMORPH**	1-HS-*syn*-$B_{18}H_{21}$ (PM1a)
CCDC	2262296
Empirical formula	B_{18} H_{22} S_1
Diffractometer	four-cycle diffractometer Cryostream, AtlasS2, Rigaku OD Supernova
M_r /g mol^{-1}	248.8 (formula weight)
T/K	95
Wavelength / Å	1.54184
Crystal system	monoclinic
Space group	$P2_1/c$
a/Å	10.0437 (6)

POLYMORPH	1-HS-*syn*-$B_{18}H_{21}$ (PM1a)
b/Å	11.3556 (6)
c/Å	13.8050 (8)
α/deg	90
β/deg	108.575 (5)
γ/deg	90
V/$Å^3$	1492.47 (15)
Z	4
Calc. density/g cm^{-3}	1.1073
μ mm^{-1}	1.536
F(000)	512
Crystal size /mm^3	0.050 x 0.042 x 0.020
θ range /°	4.6 – 73.3
Index ranges /*hkl*	-12, 12, -11, 14, -16, 16
Reflections collected (R_{int})	10154 (0.072)
Independent reflections	2952
Completeness /% to θ /°	99, 66.97
Absorption correction	multiscan
Max. and min. transmission	0. 164, 1
Data / restraints / constraints / parameters	2891 / 0 / 22 / 238
Goodness-of-fit on F^2	1.26 [(1)]

POLYMORPH	1-HS-*syn*-$B_{18}H_{21}$ (PM1a)
$R1$, $wR2$ [I>3σ(I)]	0.0452, 0.0944
$R1$, $wR2$ (all data)	0.0744, 0.1057
Largest diff. peak and hole, eÅ^{-3}	0.25, -0.31

Table S9. Crystallographic collection and refinement data for 1-HS-*syn*-$B_{18}H_{21}$ (PM1b).

	1-HS-*syn*-$B_{18}H_{21}$ (PM1b)
CCDC	2262299
Empirical formula	B_{18} H_{22} S_1
Diffractometer	four-cycle diffractometer<br>Cryostream, AtlasS2, Rigaku OD Supernova
M_r /g mol^{-1}	248.8 (formula weight)
T/K	95
Wavelength / Å	1.54184
Crystal system	monoclinic
Space group	$P2_1/n$
a/Å	10.0415(7)
b/Å	11.6578(8)
c/Å	13.4184(9)
α/deg	90

	1-HS-*syn*-$B_{18}H_{21}$ (PM1b)
β/deg	110.018(6)
γ/deg	90
V/Å^3	1475.88(18)
Z	4
Calc. density/g cm^{-3}	1.1198
μ mm^{-1}	1.553
F(000)	512
Crystal size /mm^3	0.068 x 0.047 x 0.032
θ range /°	4.80 – 72.87
Index ranges /*hkl*	-12, 11, -8, 13, -14, 16
Reflections collected (R_{int})	8326 (0.0651)
Independent reflections	2867
Completeness /% to θ /°	99, 66.49
Absorption correction	multiscan
Max. and min. transmission	0.621, 1
Data / restraints / constraints / parameters	2867 / 0 / 22 / 238
Goodness-of-fit on F^2	1.54 (1)
$R1$, $wR2$ [I>3σ(I)]	0.0451, 0.1115
$R1$, $wR2$ (all data)	0.0674, 0.1189

	1-HS-*syn*-$B_{18}H_{21}$ (PM1b)
Largest diff. peak and hole, eÅ^{-3}	0.28, -0.35

Table S10. Crystallographic collection and refinement data for 3-HS-*syn*-$B_{18}H_{21}$.

	3-HS-*syn*-$B_{18}H_{21}$
CCDC	2262294
Empirical formula	B_{18} H_{22} S_1
Diffractometer	four-cycle diffractometer Cryostream, AtlasS2, Rigaku OD Supernova
M_r /g mol^{-1}	248.8 (formula weight)
T/K	95
Wavelength / Å	1.54184
Crystal system	monoclinic
Space group	$P2_1/c$
a/Å	12.264(2)
b/Å	6.659(2)
c/Å	19.098(4)
α/deg	90
β/deg	107.06(3)

	3-HS-*syn*-$B_{18}H_{21}$
γ/deg	90
V/Å^3	1491.0(6)
Z	4
Calc. density/g cm^{-3}	1.1084
μ mm^{-1}	1.538
F(000)	512
Crystal size /mm^3	0.070 x 0.046 x 0.027
θ range /°	7.1 – 72.5
Index ranges /*hkl*	-13, 15, -8, 8, -23, 23
Reflections collected (R_{int})	8518 (0.038)
Independent reflections	2891
Completeness /% to θ /°	99, 66.49
Absorption correction	multiscan
Max. and min. transmission	0. 471, 1
Data / restraints / constraints / parameters	2891 / 0 / 22 / 238
Goodness-of-fit on F^2	1.52 (1)
R1, wR2 [I>3σ(I)]	0.0356, 0.0804
R1, wR2 (all data)	0.0469, 0.0846
Largest diff. peak and hole, eÅ^{-3}	0.24, -0.24

Table S11. Crystallographic collection and refinement data for 4-HS-*syn*-$B_{18}H_{21}$ (PM 4a).

**POLYMORPH**	4-HS-*syn*-$B_{18}H_{21}$ (PM 4a)
CCDC	2262307
Empirical formula	B_{18} H_{22} S_1
Diffractometer	four-cycle diffractometer Cryostream, AtlasS2, Rigaku OD Supernova
M_r /g mol^{-1}	248.8 (formula weight)
T/K	95
Wavelength / Å	1.54184
Crystal system	orthorhombic
Space group	Pbca
a/Å	14.4249 (5)
b/Å	11.9778 (4)
c/Å	17.8595 (6)
α/deg	90
β/deg	90
γ/deg	90
V/Å^3	3085.74 (18)
Z	8
Calc. density/g cm^{-3}	1.0712

**POLYMORPH**	4-HS-*syn*-$B_{18}H_{21}$ (PM 4a)
μ mm^{-1}	1.486
F(000)	1024
Crystal size /mm^3	0.074 x 0.044 x 0.019
θ range /°	4.9 – 72.9
Index ranges /*hkl*	-15, 17, -7, 14, -21, 18
Reflections collected (R_{int})	6528 (0.022)
Independent reflections	2994
Completeness /% to θ /°	99, 66.97
Absorption correction	multiscan
Max. and min. transmission	0. 81, 1
Data / restraints / constraints / parameters	2994 / 0 / 22 / 238
Goodness-of-fit on F^2	1.69 [(1)]
*R*1, *wR*2 [I>3σ(I)]	0.0300, 0.0720
*R*1, *wR*2 (all data)	0.0361, 0.0743
Largest diff. peak and hole, eÅ^{-3}	0.18, -0.20

Table S12. Crystallographic collection and refinement data for PM4a measured at room temperature.

	PM4a – at RT.
CCDC	2265136
Empirical formula	$SB_{18} H_{22}$
Diffractometer	four-cycle diffractometer<br>Cryostream, AtlasS2, Rigaku OD Supernova
M_r /g mol^{-1}	248.8 (formula weight)
T/K	300
Wavelength / Å	1.54184
Crystal system	orthorombic
Space group	Pbca
a/Å	17.9237 (10)
b/Å	14.6363 (8)
c/Å	12.0552 (7)
α/deg	90
β/deg	90
γ/deg	90
V/Å^3	3162.5 (3)
Z	8
Calc. density/g cm^{-3}	1.045
μ mm^{-1}	1.45
F(000)	1024

	PM4a – at RT.
Crystal size /mm^3	0.14 × 0.09 × 0.05
θ range /°	4.9 – 72.9
Index ranges /*hkl*	-21, 1, -17, 17, -4, 14
Reflections collected (R_{int})	6766 (0.028)
Independent reflections	3053
Completeness /% to θ /°	98, 71.21
Absorption correction	multiscan
Max. and min. transmission	0. 79, 1
Data / restraints / constraints / parameters	3053/ 0 / 22 / 238
Goodness-of-fit on F^2	1.75 (1)
*R*1, *wR*2 [I>3σ(I)]	0.0486, 0.1271
*R*1, *wR*2 (all data)	0.0686, 0.1388
Largest diff. peak and hole, eÅ^{-3}	0.18, -0.16

Table S13. Crystallographic collection and refinement data for 4-HS-*syn*-$B_{18}H_{21}$ (PM4b).

	4-HS-*syn*-$B_{18}H_{21}$ (PM4b)
CCDC	2262306

	4-HS-*syn*-$B_{18}H_{21}$ (PM4b)
Empirical formula	B_{18} H_{22} S_1
Diffractometer	four-cycle diffractometer Cryostream, AtlasS2, Rigaku OD Supernova
M_r /g mol^{-1}	248.8 (formula weight)
T/K	95
Wavelength / Å	1.54184
Crystal system	monoclinic
Space group	$P2_1/n$
a/Å	10.453 (2)
b/Å	12.585 (3)
c/Å	11.985 (2)
α/deg	90
β/deg	91.29 (3)
γ/deg	90
V/Å^3	1576.2(5)
Z	4
Calc. density/g cm^{-3}	1.0485
μ mm^{-1}	1.454
F(000)	512
Crystal size /mm^3	0.130 x 0.068 x 0.037

	4-HS-*syn*-$B_{18}H_{21}$ (PM4b)
θ range /°	5.1 – 73.4
Index ranges /*hkl*	-11, 12, -15, 15, -14, 11
Reflections collected (R_{int})	9348 (0.0486)
Independent reflections	3109
Completeness /% to θ /°	99, 66.97
Absorption correction	multiscan
Max. and min. transmission	0.235, 1
Data / restraints / constraints / parameters	3109 / 0 / 22 / 238
Goodness-of-fit on F^2	1.35 [(1)]
*R*1, *wR*2 [I>3σ(I)]	0.0355, 0.0781
*R*1, *wR*2 (all data)	0.0515, 0.0843
Largest diff. peak and hole, eÅ^{-3}	0.19, -0.20

(1) The refinement program Jana2020 does not refine the weighting scheme. Therefore, GOF is usually fairly above one, especially for strongly diffracting crystals.

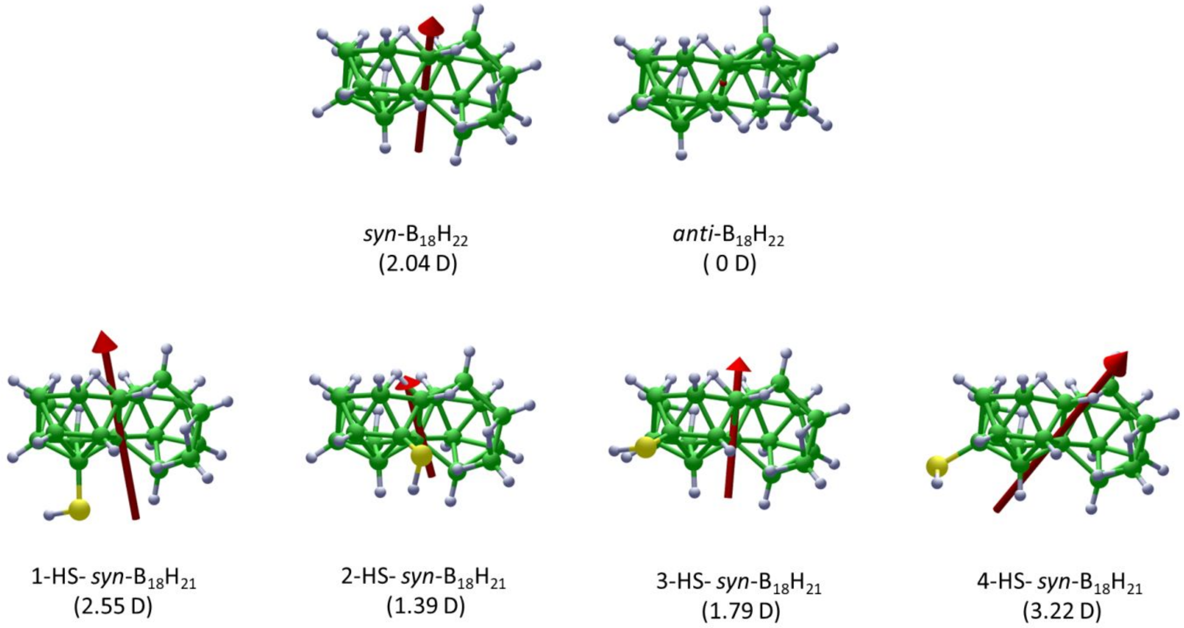

Figure S47. Graphical projection of the dipole moment vectors in the SH-$B_{18}H_{21}$ isomers as well as in the parent *syn*-$B_{18}H_{22}$ and *anti*-$B_{18}H_{22}$.

Table S14. Table of the computationally obtained dipole moment values of all isomers and the parent *syn*-$B_{18}H_{22}$.

Isomer	Dipole moment (Debye)
syn-$B_{18}H_{22}$	2.0361
anti-$B_{18}H_{22}$	0
1-HS-*syn*-$B_{18}H_{21}$	2.5538
2-HS-*syn*-$B_{18}H_{21}$	1.3852
3-HS-*syn*-$B_{18}H_{21}$	1.7931
4-HS- *syn*-$B_{18}H_{21}$	3.2160

TD-DFT

The results of the Time Dependent Density Functional Theory (TD-DFT) calculations are summarised in the Table S. Simulated spectra and calculated excited states energies of *anti*- and *syn*-$B_{18}H_{22}$ are compared in the Figure S48, those of 1-, 2-, 3-, and 4-HS-*syn*-$B_{18}H_{21}$ follow in Figure S49 to Figure S52.

Table S15: **Excitation energies (E_{exc}) and the associated wavelengths (λ) of the first ((S1) and second (S2) excited states in the ground state geometry, and that of the first singlet excited state in its optimised geometry (S1/S1)**

	E_{exc}(S1)/eV	λ(S1)/nm	E_{exc}(S2)/eV	λ(S2)/nm	E_{exc}(S1/S1)/eV	λ(S1/S1)/nm
anti-$B_{18}H_{22}$	4.2328	292.91	5.1265	241.85	3.3196	373.49
syn-$B_{18}H_{22}$	4.5570	272.07	4.7794	259.41	3.6805	336.87
1-HS-*syn*-$B_{18}H_{21}$	4.0143	308.85	4.6626	265.91	2.7267	454.70
2-HS-*syn*-$B_{18}H_{21}$	4.1112	301.58	4.6108	268.90	2.7203	455.77
3-HS-*syn*-$B_{18}H_{21}$	4.2357	292.71	4.5551	272.19	2.7193	455.94
4-HS-*syn*-$B_{18}H_{21}$	3.6743	337.44	4.5487	272.57	2.5432	487.52

Upon optimisation of the first excited singlet geometry on the TD-DFT level of theory, the calculated energy of the first excited singlet of *syn*-$B_{18}H_{22}$ above the ground state lowers from 4.56 eV only to 3.68 eV, the S1 thus remains in its relaxed geometry even a bit higher above the ground state than that of *anti*-$B_{18}H_{22}$. The differences between the optimized geometry of the ground state, and that of the first excited singlet, are for both *anti*-$B_{18}H_{22}$ (Figure S53) and *syn*-$B_{18}H_{22}$ (Figure S54) almost imperceptible. There is no indication in our TD-DFT results pointing to the existence of an easily accessible conical intersection of the first excited singlet with the ground state, claimed as responsible for the observed absence of luminescence in the solution of *syn*-$B_{18}H_{22}$ at the CASPT2/CASSCF level of theory[i]. For a correct description of the excited states of *syn*-$B_{18}H_{22}$, extensive theoretical studies at higher levels of theory are probably necessary. Also, the calculated luminescence wavelengths λ(S1/S1) of *syn*-$B_{18}H_{22}$ and all its thiol derivatives investigated are too short compared to those measured experimentally in the solid state. Similarly, the longest calculated absorption wavelength, the weak line at 337 nm of 4-HS-*syn*-$B_{18}H_{21}$ (Table S15, Figure S52), lies still in the ultraviolet range, although the species forms intensively pink crystals. These observations clearly suggest that the spectroscopic properties of these species in solid state are predominantly determined by their supramolecular crystalline structure, and their further theoretical analysis is beyond the scope of the basic DFT calculations of isolated molecules.

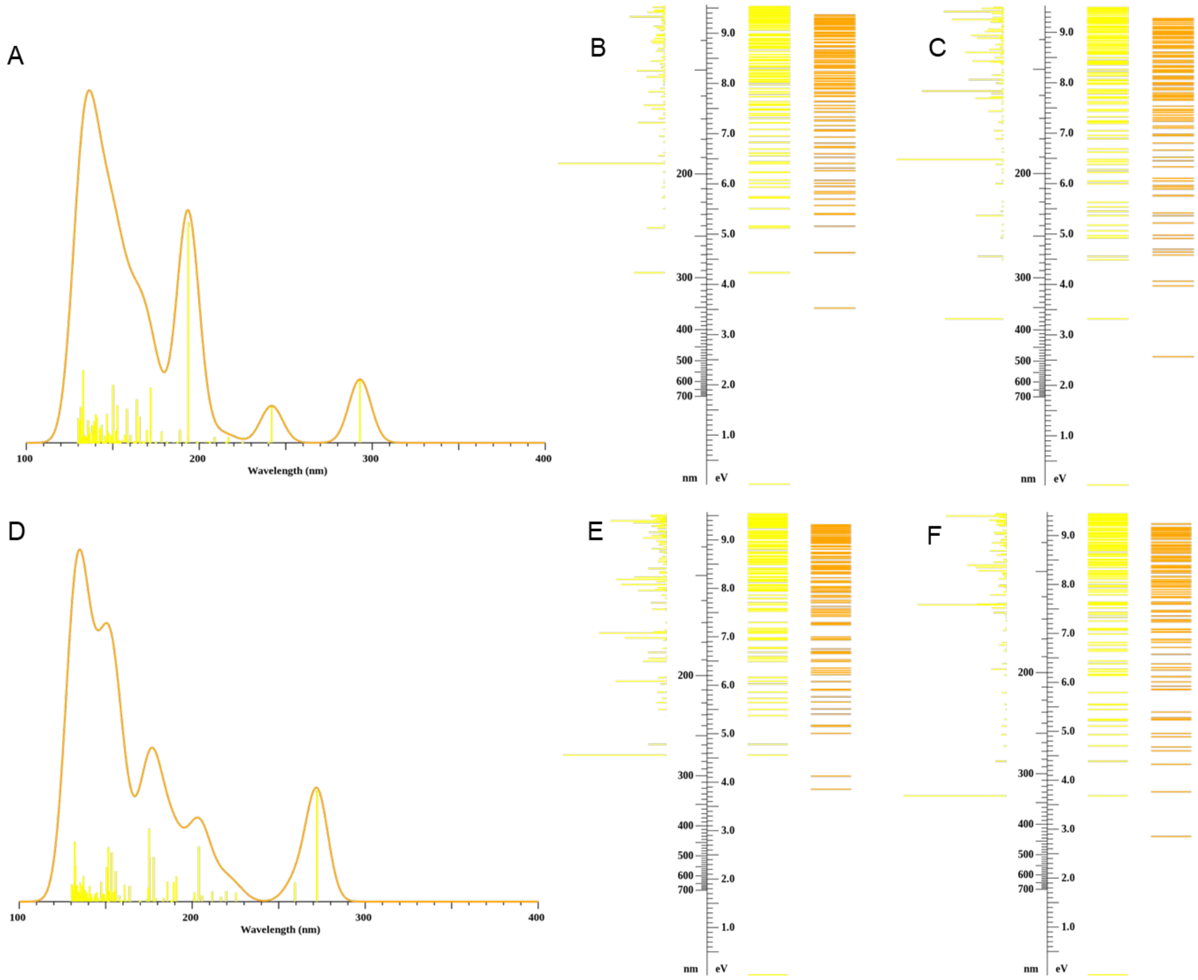

Figure S48: Simulated spectra (A, D), excited states (B, E) and excited states in the relaxed geometry of S1 (C, F) of *anti* (A-C) and *syn* (D-F) $B_{18}H_{22}$; in the excited states diagrams, singlets are depicted yellow, triplets orange, and the length of the yellow lines on the left side, with scale labels in nm, represents the relative oscillator strengths of the singlet excited states.

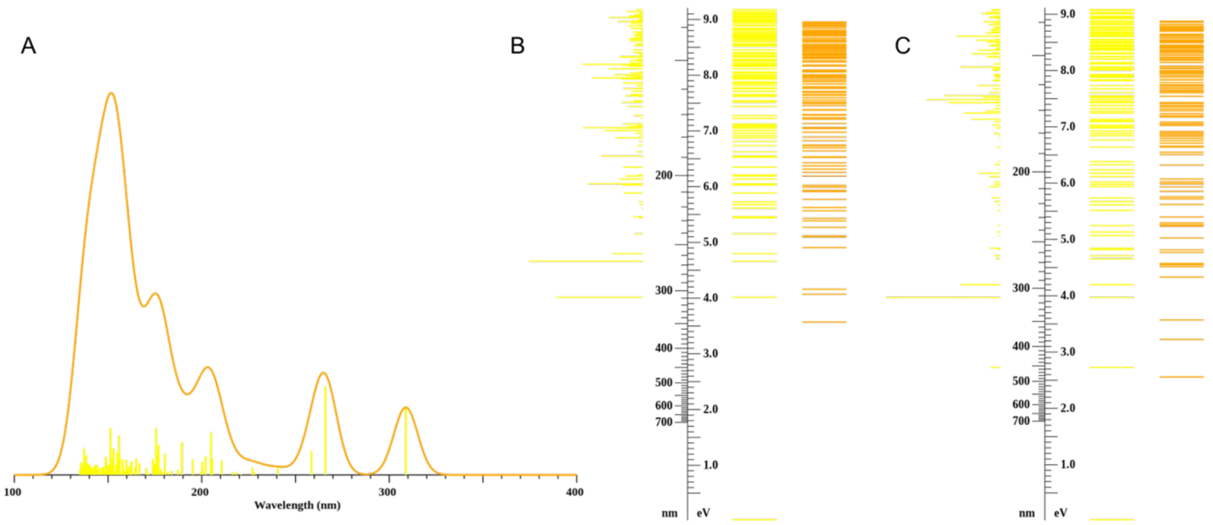

Figure S49: Simulated spectra (A), excited states (B) and excited states in the relaxed geometry of S1 (C) of 1-HS-*syn*-$B_{18}H_{21}$.

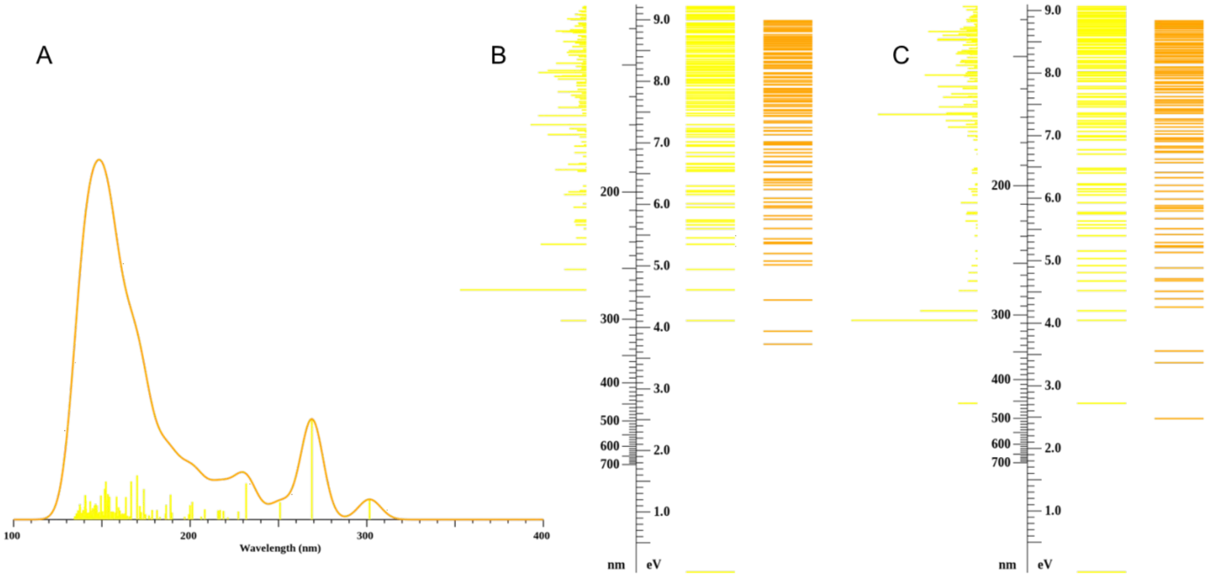

Figure S50: Simulated spectra (A), excited states (B) and excited states in the relaxed geometry of S1 (C) of 2-HS-*syn*-$B_{18}H_{21}$.

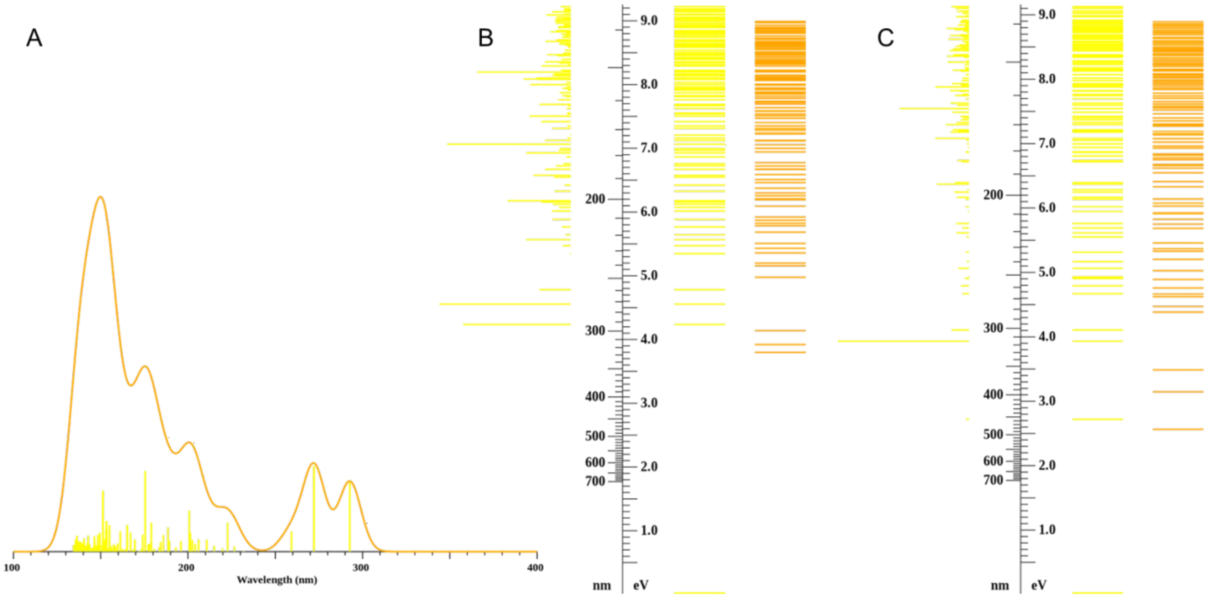

Figure S51: Simulated spectra (A), excited states (B) and excited states in the relaxed geometry of S1 (C) of 3-HS-*syn*-$B_{18}H_{21}$.

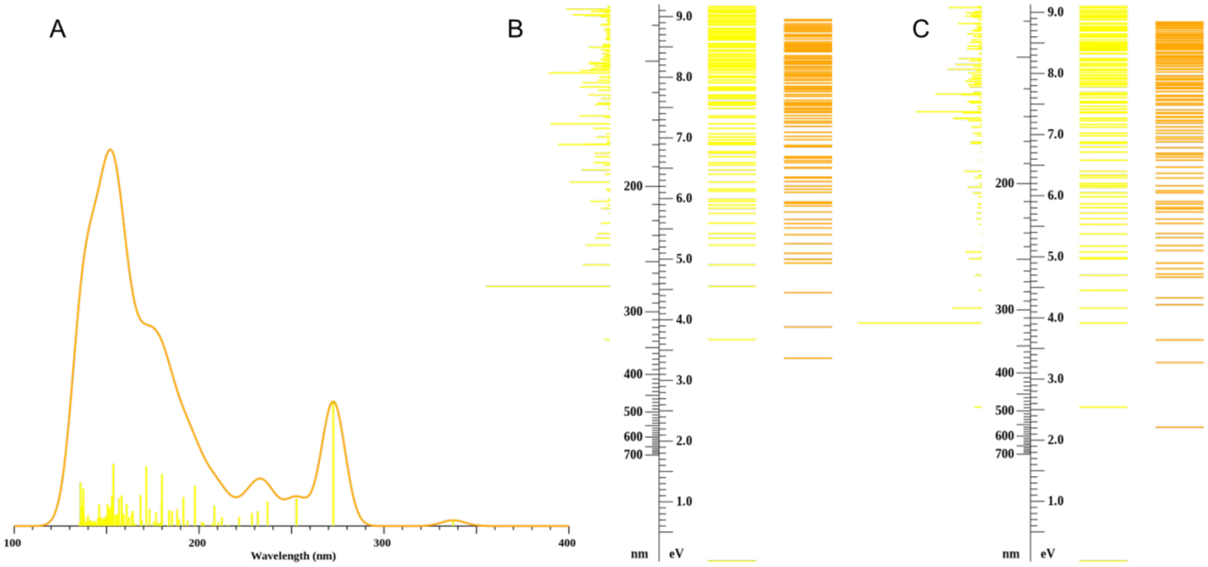

Figure S52: Simulated spectra (A), excited states (B) and excited states in the relaxed geometry of S1 (C) of 4-HS-*syn*-$B_{18}H_{21}$.

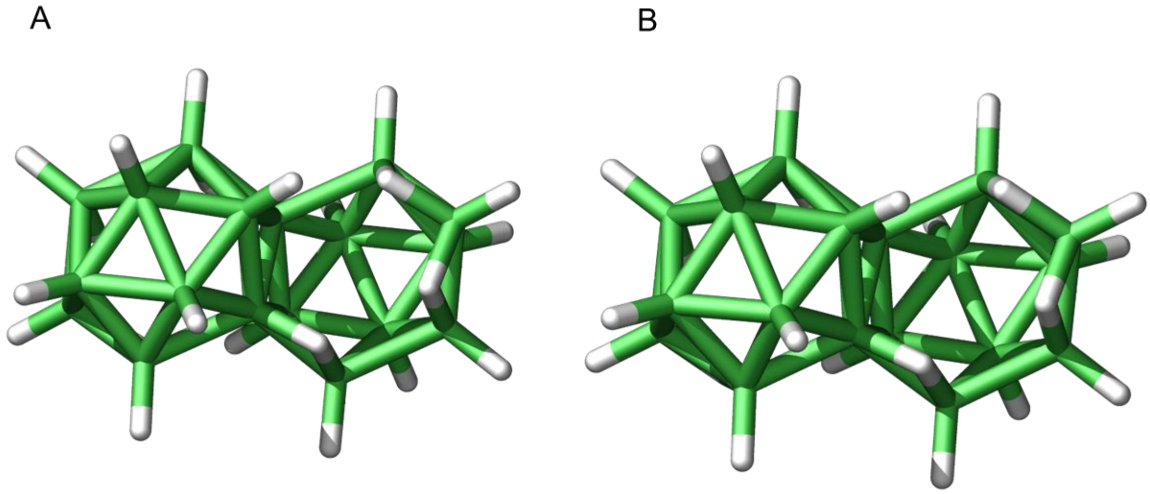

Figure S53: Optimized geometries of *anti*-$B_{18}H_{22}$: the ground state (A) and the first excited singlet (B).

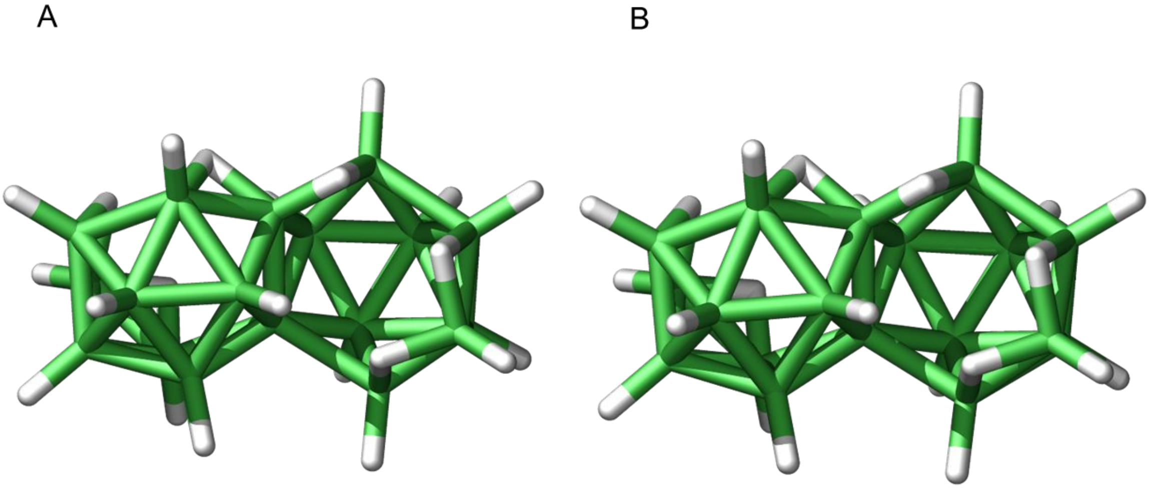

Figure S54: Optimized geometries of *syn*-$B_{18}H_{22}$: the ground state (A) and the first excited singlet (B).

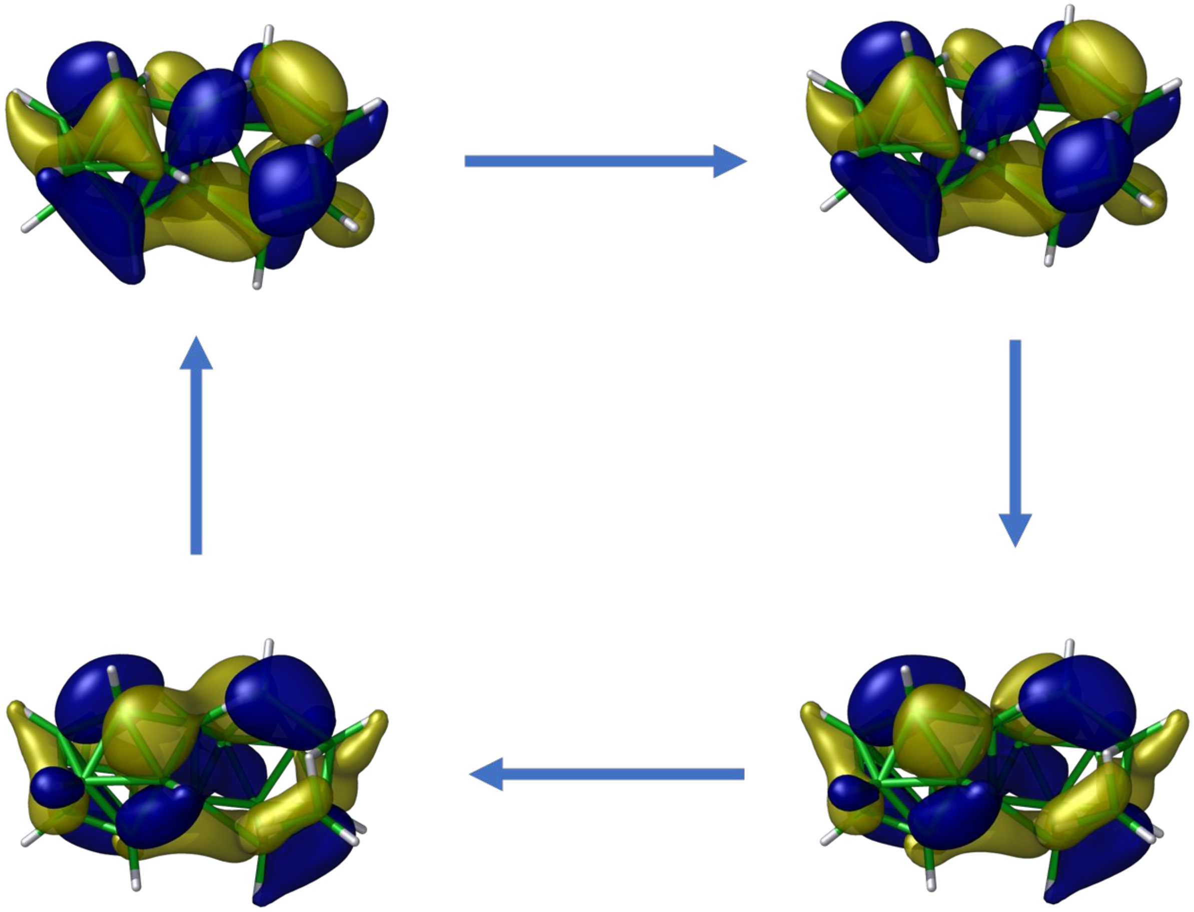

Figure S55: Natural Transition Orbitals (bottom hole, top electron) for the absorption of *syn*-$B_{18}H_{22}$ to the first excited singlet in the ground state geometry (left) and its luminescence from the relaxed geometry of S1 (right).

i Londesborough, M. G. S.; Hnyk, D.; Bould, J.; Serrano-Andrés, L.; Sauri, V.; Oliva, J. M.; Kubát, P.; Polívka, T.; Lang, K.: Distinct Photophysics of the Isomers of $B_{18}H_{22}$ Explained, *Inorg. Chem.* **2012**, *51*, 1471–1479. dx.doi.org/10.1021/ic201726k
